# Intracerebral Hemorrhage and Ischemic Stroke of Different Etiologies Have Distinct Alternatively Spliced mRNA Profiles in the Blood: a Pilot RNA-seq Study

**DOI:** 10.1007/s12975-015-0407-9

**Published:** 2015-05-22

**Authors:** Cheryl Dykstra-Aiello, Glen C. Jickling, Bradley P. Ander, Xinhua Zhan, DaZhi Liu, Heather Hull, Miles Orantia, Carolyn Ho, Boryana Stamova

**Affiliations:** Department of Neurology, University of California at Davis, Sacramento, CA USA; Department of Neurology, MIND Institute Wet Labs, 2805 50th Street, Room 2417, Sacramento, CA 95817 USA

**Keywords:** Ischemic stroke, Intracerebral hemorrhage, Alternative splicing, Biomarkers, RNA-seq, Exon

## Abstract

Whole transcriptome studies have used 3′-biased expression microarrays to study genes regulated in the blood of stroke patients. However, alternatively spliced messenger RNA isoforms have not been investigated for ischemic stroke or intracerebral hemorrhage (ICH) in animals or humans. Alternative splicing is the mechanism whereby different combinations of exons of a single gene produce distinct mRNA and protein isoforms. Here, we used RNA sequencing (RNA-seq) to determine if alternative splicing differs for ICH and cardioembolic, large vessel and lacunar causes of ischemic stroke compared to controls. RNA libraries from 20 whole blood samples were sequenced to 200 M 2 × 100 bp reads using Illumina sequencing-by-synthesis technology. Differential alternative splicing was assessed using one-way analysis of variance (ANOVA), and differential exon usage was calculated. Four hundred twelve genes displayed differential alternative splicing among the groups (false discovery rate, FDR; *p* < 0.05). They were involved in cellular immune response, cell death, and cell survival pathways. Distinct expression signatures based on usage of 308 exons (292 genes) differentiated the groups (*p* < 0.0005; fold change >|1.2|). This pilot study demonstrates that alternatively spliced genes from whole blood differ in ICH compared to ischemic stroke and differ between different ischemic stroke etiologies. These results require validation in a separate cohort.

**Electronic supplementary material:**

The online version of this article (doi:10.1007/s12975-015-0407-9) contains supplementary material, which is available to authorized users.

## Introduction

Stroke is diagnosed based on patient history, neurological exam, and brain imaging. Differential diagnosis can be difficult particularly for distinguishing ischemic stroke (IS) from intracerebral hemorrhage (ICH) when imaging is unavailable in the acute setting. Thus, an accurate, inexpensive, and rapid blood test would be useful.

Blood transcriptomes show promise as diagnostic biomarkers and have provided insight into understanding the nature of the immune response following human stroke [1–6]. However, these studies have investigated only a portion of the protein coding transcriptome, since they have used 3′-biased microarrays to measure blood mRNA expression [1–6]. Though these studies demonstrated proof-of-principle, most of the stroke transcriptome which is comprised of all alternatively spliced isoforms remains unstudied in stroke. The importance of alternative splicing is supported by evidence implicating it in the pathogenesis of many diseases [7, 8] .

Alternative splicing is the process whereby exons from a single gene are included or excluded in the final mRNA transcript (Supplementary Figure 1). A single gene can produce several alternatively spliced isoforms which have specific functions in different cells, tissues, developmental stages, and disease states. Thus, the ~20,000 known genes code for >250,000 different mRNAs and proteins. Differential alternative splicing (DAS) is alternative splicing that differs between groups. We hypothesized that DAS would vary for different causes of IS (cardioembolic, large vessel, and lacunar) and for ICH when compared to each other and to controls.

RNA-seq is a new technology that allows for estimation of expression of each splice variant (Supplementary Figure 1), a significant advance over previous technologies. Because there have been no studies of alternative splicing related to stroke etiology, or for IS versus ICH either in humans or in animal models, we performed this pilot RNA-seq study to examine DAS in whole blood following IS and ICH in humans.

## Methods

Stroke patients and control subjects were randomly selected from those recruited at the University of California Davis Medical Center between 2008 and 2012. Stroke patients were chosen to represent the major IS etiologies (cardioembolic, large vessel atherosclerotic, lacunar) or had ICH. IS diagnosis and causes were assessed as described previously [5, 9]. ICH patients had deep ICH (basal ganglia, deep white matter, or thalamus) confirmed by CT and/or MRI brain scans and were associated with hypertension without evidence of vascular malformation, tumor, or aneurysm. Control subjects were selected to match stroke subjects for age, race, sex, and vascular risk factors and had no history of previous stroke or cardiovascular events. Blood from all subjects was drawn into PAXgene tubes between 5.8 and 101.2 h following IS or ICH. RNA from whole blood was isolated as previously described [3]. The UCD Institutional Review Board approved this study and all subjects provided informed consent.

Whole blood RNA was used to prepare mRNA libraries using the TruSeq RNA Sample Prep v2 kit and protocol (Illumina). Two hundred million PE 100-bp RNA-seq reads were obtained from each mRNA library using Illumina Solexa sequencing by synthesis on the Illumina HiSeq 2000. TopHat v2.0.7 (Bowtie v2.0.6) was used with default parameters to map reads to a reference genome (Hg19) and generate *bam* files for analysis [10]. RNA transcript quantification was performed using Hg19 AceView transcripts in the Partek Genomics Suite 6.6 RNA-seq workflow.

The raw reads for genes displaying DAS are shown in Supplementary Table 2 and the raw reads for genes displaying differential exon usage are shown in Supplementary Table 6. They were generated from aligned *bam* files using *featureCounts* against AceView (NCBI 37) [11] with options allowing for any and multiple overlaps [12]. However, they were not used directly for the statistical analysis. Instead, raw aligned reads were normalized, and differential alternatively spliced transcript expression and exon expression quantification were performed using the expectation/maximization (E/M) algorithm (briefly described below) as implemented in Partek Genomics Suite [13]. DAS was determined with one-way ANOVA on Group (Benjamini-Hochberg false discovery rate, FDR; *p* < 0.05), and differential exon usage was assessed between each two groups (*p* < 0.0005, fold change > |1.2|).

Principal components analysis (PCA) and hierarchical clustering were performed in Partek Genomics Suite. Ingenuity Pathway Analysis (IPA®) and DAVID identified regulated pathways and processes as described previously [6].

## Results

### Subject Demographics

Subject demographics and clinical characteristics are presented in Table 1. Only Caucasian males were studied because of the small group sizes. Age, time since event for IS or ICH, and vascular risk factors were not significantly different between groups. Coverage of a wide range of post-stroke biology was obtained by selecting patients with early (5.8 h) through late (101.2 h) blood draw times after IS and ICH. However, the means were similar between the stroke groups. Cardioembolic IS post-event blood draw times were, on average, 33.7 h; large vessel averaged 47.4 h; lacunar averaged 34.6 h; and ICH averaged 29.4 h (Table 1).

**Table 1:** Subject demographics and clinical characteristics

	Ischemic stroke			Intracerebral hemorrhage	Controls
	Cardioembolic	Large vessel	Lacunar		
Subjects (total *n* = 20)	4	4	4	4	4
Age, years (mean ± SD)	62.3 ± 9.6	61.0 ± 8.2	58.9 ± 9.0	60.1 ± 2.3	60.8 ± 9.2
Time since event, h (mean ± SD)	33.7 ± 18.9	47.4 ± 47.8	34.6 ± 23.7	29.4 ± 15.5	N/A
Hypertension	4	3	2	3	3
Diabetes	2	2	0	0	1
Hyperlipidemia	3	2	2	0	2

### RNA Sequencing Alignments

RNA sequencing alignment statistics for all samples among the five groups are presented in Supplementary Table 1. Cardioembolic stroke samples had on average, 1.60E+08 alignments; large vessel had 1.65E+08 alignments; lacunar stroke had 1.64E+08 alignments; and ICH and control groups each averaged 1.59E+08 alignments. These data show that there is no bias in the numbers of alignments for any of the five groups.

### Distinct Alternative Splicing of Genes in Whole Blood of Stroke Patients and Controls

A total of 412 genes displayed differential alternative splicing (DAS) in the whole blood transcriptomes of the five groups of patients with ischemic stroke (cardioembolic, large vessel, and lacunar), intracerebral hemorrhage (ICH) and controls (FDR *p* < 0.05; Supplementary Table 2, raw reads; Supplementary Table 3, ANOVA results). These 412 genes are those predicted using the E/M algorithm [13] as implemented in Partek, to have DAS for IS (cardioembolic, large vessel, lacunar) versus ICH versus controls. The E/M algorithm probabilistically assigns reads to known isoforms/exons of a gene [13]. Partek then uses a log-likelihood ratio test to identify genes with DAS across samples [13, 14]. The 412 significant genes displaying DAS across IS, ICH, and controls are involved in cellular immunity, cytokine signaling, and cell death and survival pathways (Supplementary Table 4).

Pathways highly over-represented with differentially alternative spliced genes between the five groups included CD28 signaling in T helper cells, CDC42 signaling, Nur77 signaling in T lymphocytes, fMLP signaling in neutrophils, and interferon signaling (Supplementary Table 4). Molecular and cellular functions most highly associated with the differentially alternatively spliced genes were cell death and survival of immune cells, cell-cell signaling, activation and recruitment of leukocytes, antigen-presenting cells, activation of T lymphocytes, adhesion of vascular endothelial cells, and immune response of neutrophils (Supplementary Table 5).

### Specific Exon-Usage Profiles for ICH and Different Ischemic Stroke Etiologies

**Fig. 1:**
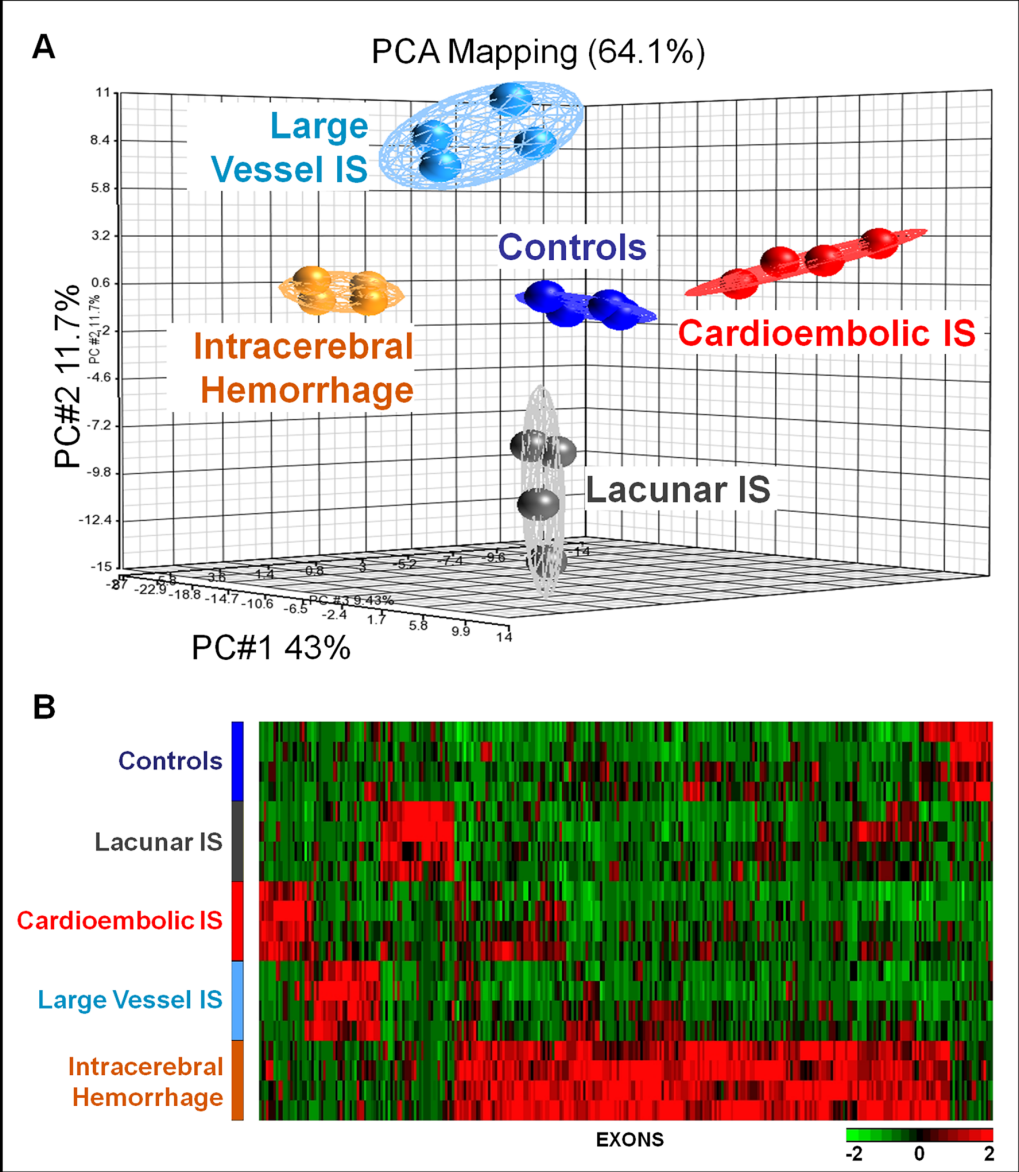
Principal components analysis (PCA) (Fig. 1a) and unsupervised hierarchical clustering (Fig. 1b) of the 308 exons (292 genes) with differential exon usage among intracerebral hemorrhage (*n* = 4), ischemic strokes (IS) (cardioembolic, large vessel, and lacunar) (*n* = 12), and control subjects (*n* = 4). In Fig. 1a, the expression of the 308 exons is compressed on to three axes in the PCA plot. The three principal components on the PCA plot account for 64.1 % of the variance. In Fig. 1b, exon expression is shown on the *X*-axis and subjects are shown on the *Y*-axis. *Each row on the Y-axis* represents a single individual, with five individuals per group. The dendrograms were removed from this figure. *Red* indicates increased expression. *Green* indicates decreased expression

A total of 308 exons from 292 genes were differentially expressed for the three causes of IS (cardioembolic, large vessel, lacunar), ICH, and controls (*p* < 0.0005, fold change >|1.2|; Supplementary Table 6, raw reads; Supplementary Table 7, ANOVA results). These exons separated the five groups, including the three causes of IS (cardioembolic, large vessel, lacunar), ICH, and controls on principal components analysis (PCA) plots (Fig. 1a) and using unsupervised hierarchical clustering (Fig. 1b). Given that the E/M algorithm uses counts of the numbers of reads on each exon [13, 14], the differential expression of exons across the five groups represents differential exon usage across the five groups. These results are relevant to DAS because DAS results from differential exon usage.

Biological functions and networks represented by genes with differentially expressed exons in each group (Fig. 1b) are summarized in Supplementary Table 8. Cardioembolic stroke genes with differential exon usage were involved in ion binding/transport and cellular assembly/organization. Large-vessel stroke genes were associated with cell death, transcription, and chromatin remodeling. Lacunar stroke genes were associated with cellular compromise, cell cycle, cell death and survival. ICH genes were involved with protein transport and localization (Supplementary Table 8).

## Discussion

Although differential alternative splicing (DAS) is implicated in many human diseases, this is the first study to show that DAS differs between intracerebral hemorrhage (ICH), ischemic stroke, and control subjects. In addition, it is the first study to show that DAS differs between different etiologies of ischemic stroke including cardioembolic, large vessel, and lacunar causes. Identification of DAS in RNA from whole blood for specific stroke etiologies and ICH suggests the immune response varies for each condition. This will be important for understanding the pathogenesis of each condition and will be important for developing biomarkers to differentiate ischemic stroke from ICH and for developing biomarkers to differentiate the different causes of ischemic stroke.

This study identified several pathways, molecular functions, and genes previously reported in human ischemic stroke using 3′-biased microarrays [6, 15]. These included actin cytoskeleton signaling, CCR5 signaling in macrophages, NF-κB activation, α-adrenergic signaling, cellular growth and proliferation, cell death and survival, cell morphology, hematopoiesis, hematological system development, and inflammatory response [4, 5, 16, 17]. Moreover, a number of the pathways implicated in different etiologies of ischemic stroke in our previous microarray studies were confirmed in these RNA-seq studies [4, 5, 16, 17].

This study is the first to describe genes with DAS and pathways unique for ICH. Among the genes that differentiated ICH from IS were INPP5D (inositol polyphosphate-5-phosphatase) and ITA4 (integrin alpha 4). The INPP5D enzyme regulates myeloid cell proliferation and programming, and its expression correlates with hemorrhagic transformation of ischemic stroke [18]. ITA4 is involved in leukocyte recruitment after intracerebral hemorrhage [19], and leukocytes are intimately associated with ICH. For example, leukocytes are involved in clotting and interact with injured vessels and brain following ICH [15].

Other genes with DAS associated with ICH in this study included NAV1 (neuron navigator 1), PDGFC (platelet derived growth factor C), and CCM2 (cerebral cavernous malformation 2) which participate in vascular endothelial growth factor (VEGF) signaling, which predisposes the brain to hemorrhage because of new vessel formation [20]. Of interest, mutations of CCM2 cause cerebral cavernous malformations which can lead to intracerebral hemorrhage [21]. Other genes with DAS associated with ICH included EXOSC1 (exosome component 1) and EXOSC9 (exosome component 9) which code for core components of the exosome complex [22]. Although exosomes have been implicated in neuroinflammation, neurodegeneration, and cancer, they have not previously been associated with ICH [23, 24]. Lastly, another gene with DAS associated with ICH included DGCR8 (DiGeorge syndrome critical region 8, a microprocessor complex subunit) which is involved in the biogenesis of microRNAs [25], which could suggest that miRNAs are involved with differential alternative splicing following ICH.

## Study Limitations

Sample sizes in this pilot study were small. Thus, we cannot rule out splicing changes due to vascular risk factors. However, hierarchical clustering of the differentially expressed exons demonstrates separation on diagnosis and not on vascular risk factors (Supplementary Figure 2). Validation of these findings in a separate cohort is needed to confirm the present results. These results are important because they provide evidence for differential alternative splicing in the pathophysiology of the immune response to ischemic stroke and intracerebral hemorrhage and also might provide novel biomarkers for ICH and different causes of IS.

## Electronic Supplementary Material

Supplementary Figure 1 Schematic of Alternative Splicing. The 5′ and 3′ untranslated regions in mRNA are not depicted. In this example the primary mRNA transcript is transcribed into three different mRNA (mRNA1, mRNA2, mRNA3) which are translated into three different proteins (protein1, protein 2, protein 3) which are all derived from a single gene. (GIF 42 kb)

High Resolution (TIFF 317 kb)

Supplementary Figure 2 Unsupervised Hierarchical Clustering of 308 exons (292 genes) with differential exon usage among Intracerebral Hemorrhage (*n* = 4), Ischemic Strokes (Cardioembolic, Large Vessel, and Lacunar) (*n* = 12) and Control Subjects (*n* = 4). Dendrograms are not displayed. This is similar to Fig. 1, with the addition of information on age, time since event, diabetes, hypertension and hyperlipidemia. Exon expression is on the *X*-axis. Subjects are on the Y axis. Red indicates increased expression and green indicates decreased expression. (GIF 145 kb)

High Resolution (TIFF 2305 kb)

Supplementary Table 1 Over all details of the RNA sequencing reads and quality. (PDF 36 kb)

Supplementary Table 2 Raw sequencing count for the 412 genes displaying Differential Alternative Splicing (DAS). IS, Ischemic Stroke; CE, Cardioembolic IS; LV, Large Vessel IS; ICH, Intracerebral Hemorrhage. (PDF 60 kb)

Supplementary Table 3 The 412 genes with DAS among Large Vessel Ischemic Stroke (IS), Cardioembolic IS, Lacunar IS and ICH and Controls (ANOVA, FDR corrected *p* < 0.05). (PDF 72 kb)

Supplementary Table 4 Canonical pathways of the 412 genes with DAS among Large Vessel Ischemic Stroke (IS), Cardioembolic IS, Lacunar IS, Intracerebral Hemorrhage (ICH) and Control subjects. Benjamini-Hochberg corrected *P* values for multiple comparison corrections. (PDF 51 kb)

Supplementary Table 5 Functions associated with the top 2 molecular and cellular functions over-represented in the 412 differentially alternatively spliced genes. (PDF 113 kb)

Supplementary Table 6 Raw sequencing count for the 308 exons displaying differential exon usage for Ischemic Stroke (Cardioembolic, Large Vessel, Lacunar), ICH and Controls. (PDF 53 kb)

Supplementary Table 7 Differential exon usage for the 308 exons for Large Vessel IS, Cardioembolic IS, Lacunar IS, ICH and Controls (*p* < 0.0005, FC > |1.2|). (PDF 84 kb)

Supplementary Table 8 Over-represented pathways and gene ontology for the 308 exons that showed differential exon usage between the five groups (Large Vessel IS, Cardioembolic IS, Lacunar IS, ICH, Controls). (PDF 65 kb)
