# Supplementary figures and images for: Intracerebral Hemorrhage and Ischemic Stroke of Different Etiologies Have Distinct Alternatively Spliced mRNA Profiles in the Blood: a Pilot RNA-seq Study
Source: Transl Stroke Res. 2015 May 22;6(4):284–9. doi: 10.1007/s12975-015-0407-9 (PMC4485700; doi:10.1007/s12975-015-0407-9)

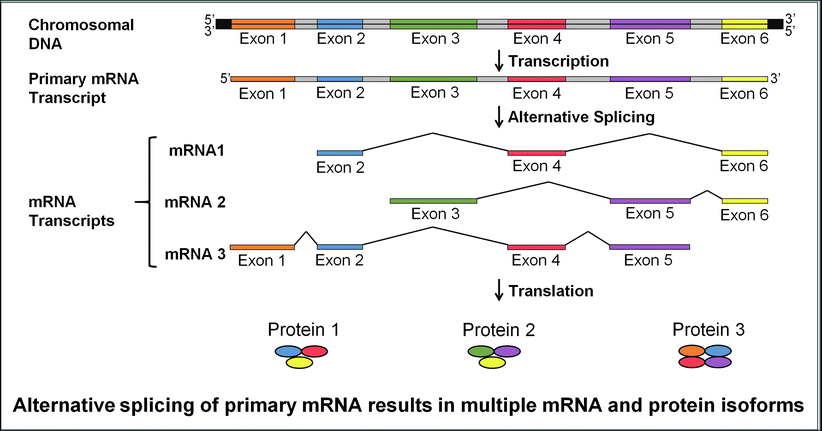

Supplement: Supplementary file 1 — Schematic of Alternative Splicing. The 5′ and 3′ untranslated regions in mRNA are not depicted. In this example the primary mRNA transcript is transcribed into three different mRNA (mRNA1, mRNA2, mRNA3) which are translated into three different proteins (protein1, protein 2, protein 3) which are all derived from a single gene. (GIF 42 kb) [file 12975_2015_407_Fig2_ESM.gif]

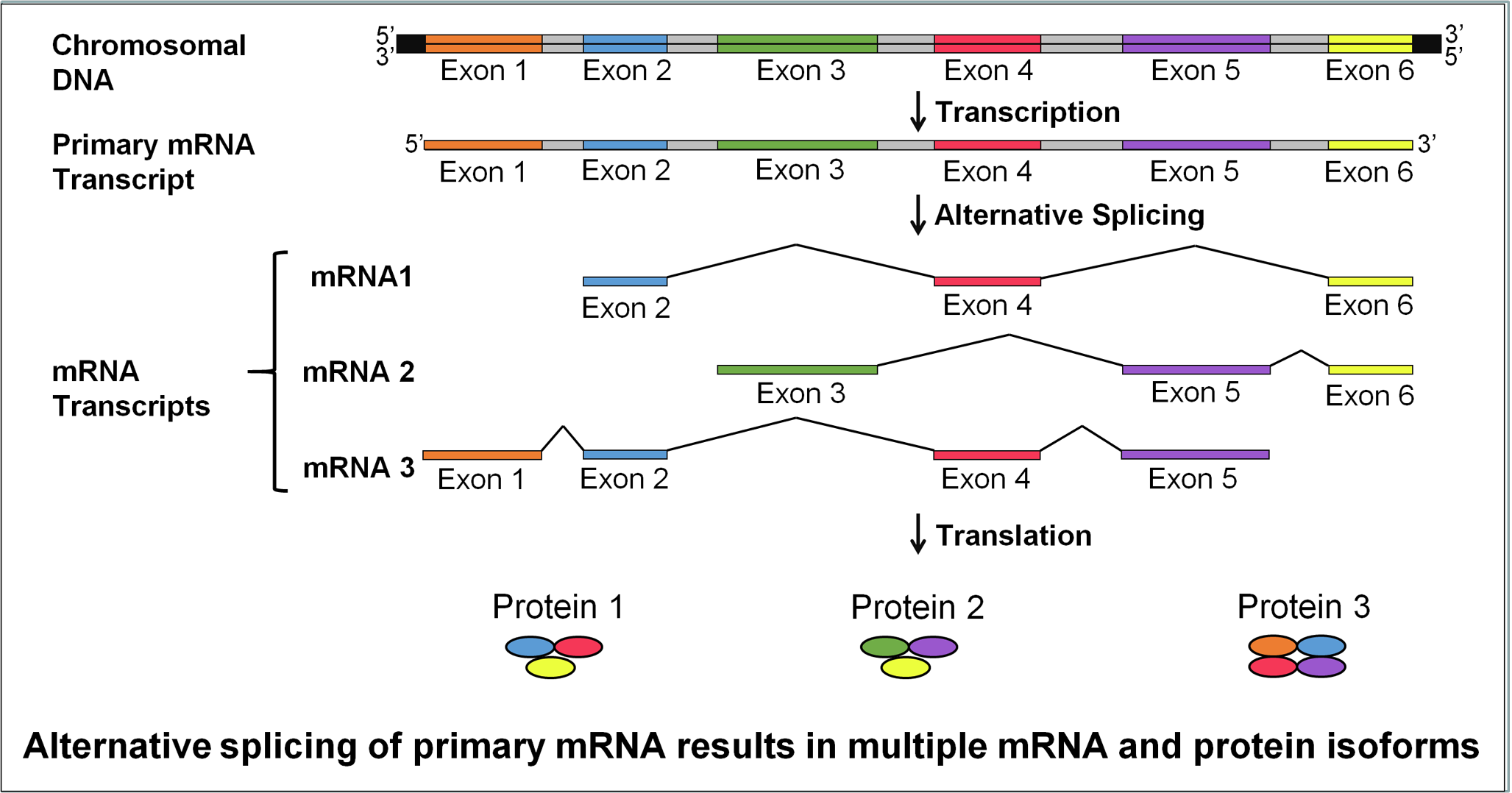

Supplement: Supplementary file 2 — High Resolution (TIFF 317 kb) [file 12975_2015_407_MOESM9_ESM.tif]

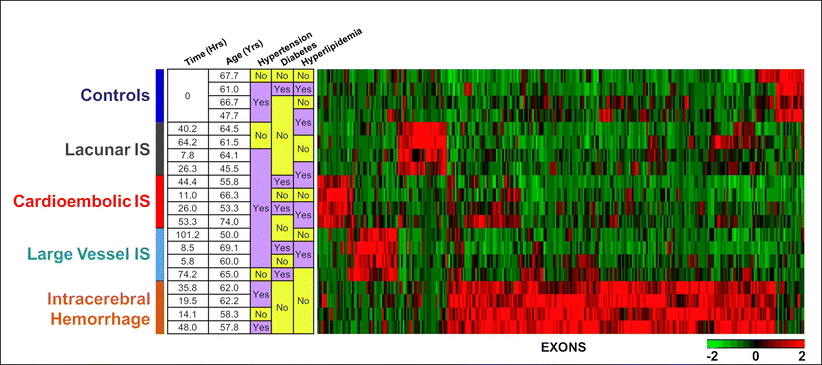

Supplement: Supplementary file 3 — Unsupervised Hierarchical Clustering of 308 exons (292 genes) with differential exon usage among Intracerebral Hemorrhage (n = 4), Ischemic Strokes (Cardioembolic, Large Vessel, and Lacunar) (n = 12) and Control Subjects (n = 4). Dendrograms are not displayed. This is similar to Fig. 1, with the addition of information on age, time since event, diabetes, hypertension and hyperlipidemia. Exon expression is on the X-axis. Subjects are on the Y axis. Red indicates increased expression and green indicates decreased expression. (GIF 145 kb) [file 12975_2015_407_Fig3_ESM.gif]

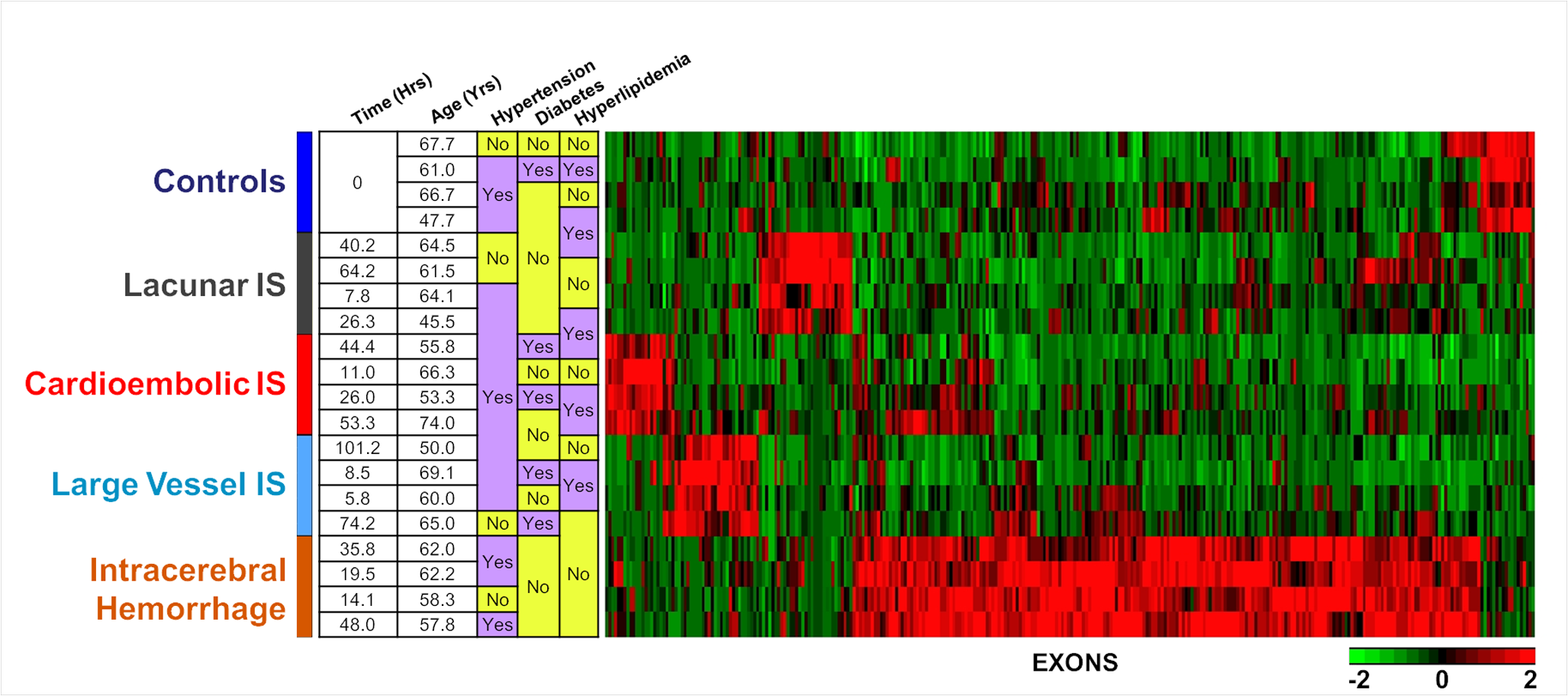

Supplement: Supplementary file 4 — High Resolution (TIFF 2305 kb) [file 12975_2015_407_MOESM10_ESM.tif]
