# Supplementary material for: Intracerebral Hemorrhage and Ischemic Stroke of Different Etiologies Have Distinct Alternatively Spliced mRNA Profiles in the Blood: a Pilot RNA-seq Study
Source: Transl Stroke Res. 2015 May 22;6(4):284–9. doi: 10.1007/s12975-015-0407-9 (PMC4485700; doi:10.1007/s12975-015-0407-9)
Supplement: Supplementary file 5 — Over all details of the RNA sequencing reads and quality. (PDF 36 kb) [file 12975_2015_407_MOESM1_ESM.pdf]

Supplementary Table 1. Details of RNA-sequencing reads and quality

All Samples	
**Sequence Length**	100
**Average Quality per Read PHRED score**	37
**Total Sequence Reads Mean ± SD**	1.95E+08 ± 1.30E+07
**Total Alignments Mean ± SD**	1.61E+08 ± 1.01E+07
**%GC Alignments Mean ± SD**	54.20 ± 1.91
**Unmapped Sequences Mean ± SD**	3.33E+07 ± 4.44E+06
**%GC Unmapped Sequences Mean ± SD**	61.60 ± 3.45

Total Sequence Reads	Ischemic Stroke			Intracerebral Hemorrhage	Controls
	Cardioembolic	Large Vessel	Lacunar		
Mean ± SD	1.93E+08 ± 1.07E+07	1.99E+08 ± 1.14E+07	1.99E+08 ± 1.18E+07	1.91E+08 ± 1.53E+08	1.92E+08 ± 1.94E+09
**Total Sequence Reads by Sample**					
1	177,117,828	203,837,628	203,564,103	191,131,791	185,714,780
2	195,150,937	182,750,587	213,336,146	199,832,252	218,687,770
3	198,658,650	209,078,152	188,188,692	169,147,880	172,555,000
4	200,504,201	198,375,293	190,420,476	202,961,105	192,521,761

Number of Alignments	Ischemic Stroke			Intracerebral Hemorrhage	Controls
	Cardioembolic	Large Vessel	Lacunar		
Mean ± SD	1.60E+08 ± 9.73E+06	1.65E+08 ± 1.25E+07	1.64E+08 ± 8.34E+06	1.59E+08 ± 1.18E+07	1.59E+08 ± 1.18E+07
CV (%)	6.073	7.61352	5.09486	7.42897	7.46071
Mean %GC ± SD	54.75 ± 2.36	55.75 ± 0.96	53.75 ± 1.89	52.00 ± 0.82	54.75 ± 1.26
**Alignments by Sample**					
1	164,675,690	171,375,517	166,034,796	160,755,434	157,275,379
2	164,012,069	146,190,367	174,444,217	168,409,440	174,121,248
3	145,627,854	172,988,643	157,269,390	142,396,412	145,330,668
4	166,272,000	168,785,456	156,892,385	166,405,048	157,539,078

Number of Unmapped Sequences	Ischemic Stroke			Intracerebral Hemorrhage	Controls
	Cardioembolic	Large Vessel	Lacunar		
Mean ± SD	3.27E+07 ± 3.14E+06	3.37E+07 ± 3.28E+06	3.52E+07 ±3.66E+06	3.13E+07 ± 4.05E+06	3.38E+07 ± 7.94E+06
Mean %GC ± SD	63.25 ± 2.63	62.25 ± 1.26	61.75 ± 1.89	57.25 ± 4.86	63.5 ± 2.38
**Unmapped Sequences by Sample**					
1	35,828,511	32,462,111	37,529,307	30,376,357	28,439,401
2	34,646,581	36,560,220	38,891,929	31,422,812	44,566,522
3	31,489,974	36,089,509	30,919,302	26,751,468	27,224,332
4	28,878,937	29,589,837	33,528,091	36,556,057	34,982,683
