# Supplementary material for: Intracerebral Hemorrhage and Ischemic Stroke of Different Etiologies Have Distinct Alternatively Spliced mRNA Profiles in the Blood: a Pilot RNA-seq Study
Source: Transl Stroke Res. 2015 May 22;6(4):284–9. doi: 10.1007/s12975-015-0407-9 (PMC4485700; doi:10.1007/s12975-015-0407-9)
Supplement: Supplementary file 6 — Raw sequencing count for the 412 genes displaying Differential Alternative Splicing (DAS). IS, Ischemic Stroke; CE, Cardioembolic IS; LV, Large Vessel IS; ICH, Intracerebral Hemorrhage. (PDF 60 kb) [file 12975_2015_407_MOESM2_ESM.pdf]

Supplementary Table 2. Raw Sequencing Count for the 412 Genes Displaying Differential Alternative Splicing. IS, Ischemic Stroke; CE, Cardioembolic; LV, Large Vessel; ICH, Intracerebral Hemorrhage

	IS_CE		IS_LV		IS_Lacunar		ICH		Controls	
	Ave	SD	Ave	SD	Ave	SD	Ave	SD	Ave	SD
ABCA7	16403	5770	12377	3225	18234	3693	19933	2498	16039	4233
ACSL4andKCNE1L	6272	3264	4595	1360	4628	730	9098	1490	4827	1841
ACTR2	28739	12256	26706	2227	30065	3565	48845	2321	28908	9358
ACTR3	19114	6293	18595	3173	18587	2316	29677	1463	17746	4849
ADCK2andNDUFB2	2119	648	2472	921	2695	702	3272	665	2170	537
ADCY7	12469	3986	11237	1876	12065	895	18247	3323	11507	2875
ADD3	9532	2315	9985	2419	10765	3958	15717	2238	9425	2159
ADSSandTGIF2P1	2348	943	2768	678	2870	368	4384	857	2695	630
AKAP8	1844	703	2070	399	2712	1281	2757	516	2018	369
ANAPC13	1374	590	1519	465	1631	144	2239	264	1412	369
ANKRD12	3670	1233	3409	1138	4141	1712	6112	732	3161	1092
ANKRD13A	12187	3824	12422	2146	15044	3991	17670	1833	11880	2161
ANXA1	13211	5310	10788	6389	8183	3672	17739	2842	6928	1076
ANXA7	4711	2477	4840	899	5558	749	7272	1389	4706	932
AP1S2	5355	1380	6032	1888	6258	2282	9031	1494	4486	317
APAF1	7159	3100	5155	1284	6900	1605	9873	1001	5654	1388
APH1A	5867	2463	7153	1450	8319	1543	9348	1780	6882	1616
APIP	317	80	533	148	687	446	762	173	510	150
APOBEC3AandAPOBEC3B	33001	17450	26057	10164	21374	11642	31795	8434	19480	4717
ARCN1	5766	2350	6389	999	7888	931	9225	1444	6955	1799
ARFIP1andFHDC1	2466	1093	1814	465	2147	492	3413	328	1948	444
ARID4BandRBM34	4830	1506	4490	1222	5205	2126	7082	456	4009	808
ARL6IP5	8648	2045	10963	3073	9913	1795	15219	1897	8910	1307
ARNTL	3184	1085	2727	705	2527	636	4147	595	2688	651
ARPC3andANAPC7	18829	5607	19036	4871	20260	5545	25612	1857	17264	3193
ARPC4andTTLL3	23650	7622	23895	3539	25514	4438	31752	3727	24837	5887
ARPC5L	1229	350	1636	241	1792	726	1957	555	1403	585
ATM	8313	2728	6946	2083	9147	3732	12223	2561	7325	2961
ATP2B4	7555	3314	6608	1708	8302	2160	10958	312	7716	2864
ATP5BandSNORD59AandSNORD59B	13855	5093	17261	4044	17332	3346	21811	4456	15419	4136
ATP6V1G2andBAT1andSNORD84	23245	6624	26641	7472	31857	13402	36453	6256	23479	5228
ATXN1LandKIAA0174	12013	4578	12239	1629	14980	2596	17972	1765	13518	3833
AZIN1	4031	1538	3338	355	4796	1597	5633	480	3764	1274
baboy	270	112	205	59	332	76	321	85	264	92
BAZ1A	7713	2400	6492	1941	7165	1487	10861	2112	5945	1143
BAZ2B	5327	2158	3992	1459	4783	1604	6859	1856	4434	1675
BTN2A2andBTN3A1	16116	4207	17885	5753	16331	4334	24720	6318	15341	4741
C1orf59	672	269	726	134	688	267	1097	226	652	186
C1orf63	15960	6039	12931	4648	15073	5086	21172	2002	11355	3017
C5orf15	1445	480	1657	450	1813	393	2496	437	1505	370
C6orf62	14572	5065	13715	4983	14069	3218	21582	1560	12323	4523
C7orf27	3177	1187	3723	1252	4203	751	4575	1131	4003	539
C9orf72	2935	415	3001	1634	2672	326	5328	780	2227	777
C9orf114	1774	524	2111	721	2899	1160	2532	843	2202	528
C11orf73	432	114	608	236	677	301	801	178	440	132
C15orf29	2112	548	1868	754	2435	783	4101	1486	1709	634
CAB39	10410	3634	8699	1429	9957	1450	14383	1474	9757	2706
CALM1	15350	6917	18863	3466	19782	2920	25933	5933	18667	5625
CALM2andC2orf61	15479	6516	14663	4393	16275	1160	26210	3036	13448	3915
CAPZA2	5544	1890	4938	2718	4393	952	8937	1790	3697	1070
CARD8	16552	6571	11578	3320	12396	1242	22249	2382	12484	4163
CBARA1	2963	1293	3481	676	3339	308	4784	816	3169	643
CCAR1	1732	422	2044	618	2428	1176	2925	594	1644	437
CCNDBP1	8467	856	7506	1801	7641	1103	10334	399	6875	1945
CCNY	6529	3154	7093	1311	7729	1703	10694	1162	7570	2006
CCT8	3356	1194	4697	1053	4875	1307	6036	1217	4007	1121
CD36	7954	1387	9710	2338	7659	4520	15417	1591	7001	1479
CD46	21708	8416	18675	8018	17812	3201	33673	4748	17804	7898
CD47	7345	2437	8764	2606	9940	5563	13406	2631	7591	1952
CD53	42068	12928	39273	8328	44675	2729	57289	3122	40645	11486
CD58	4209	2040	3251	967	3020	725	4616	283	2810	561
CD74	112018	42143	209063	81639	331003	229417	177070	87421	184352	59800
CD86	2047	619	2816	608	2829	808	3483	608	2345	615
CD164	12390	3381	11758	4777	12071	1050	21527	837	10244	3257
CD244	2066	886	2464	307	2040	787	3671	1396	2642	851
CD300E	7631	1615	11689	2852	8484	4703	14330	1670	8297	1909

CDC42SE1	53805	18532	43575	8750	50742	4383	67343	2849	49090	14021
CDC42SE2	8402	3902	10903	2619	10808	2631	14775	2813	9519	2049
CDKL3andPPP2CA	5362	1788	5760	890	6441	1116	8106	739	5381	1170
CDKN1C	475	258	793	414	636	513	980	677	1694	1119
CECR1	13296	6623	17941	3372	18100	4871	22876	7354	15490	2912
CELF2	15444	6497	11348	2899	12286	1284	18614	1426	11696	3468
CFLARandRNU7-45P	45638	20822	29598	10088	36671	5858	51915	11467	34262	10964
CGGBP1	4747	1260	4742	1664	5899	2522	7825	894	4422	1324
CHMP2B	1585	516	1665	413	1780	267	2589	135	1539	311
CLDND1	2275	597	3150	969	2654	673	4045	680	1973	510
CLEC7A	16330	7234	14100	5400	10363	3669	24725	4734	13111	4509
CLTC	9841	5008	9212	703	10965	1297	15757	1860	10074	3512
CNIH	1269	293	1393	422	1525	402	1988	114	1144	337
CNOT6L	3553	1766	3933	797	4164	941	6067	922	3869	1188
CNOT7	2961	986	3239	775	4228	2043	4706	952	3071	664
CNOT8	4484	1366	4596	927	4754	588	7258	679	4212	1070
COMMD2	831	236	940	245	1074	371	1382	302	795	161
CRKL	4800	1996	4701	385	6424	689	7280	655	5687	1825
CSGALNACT2	4666	2003	3319	700	3196	477	6621	446	3181	825
CTDSP2	23850	10326	21350	4179	27080	2291	33393	5402	26496	7398
CTSS	99290	31616	97874	25481	85337	24635	148955	14296	84027	18791
CYBB	25714	8231	34085	10285	25079	12875	42388	12992	25060	6402
CYBRD1	2790	1092	1984	890	2450	514	4998	1404	2306	705
CYLD	8303	2744	10031	3597	9191	2010	13389	1704	8426	2562
DAP3	3101	696	3779	973	4248	1356	4508	806	3322	686
DCP2	5982	2195	4905	1487	5189	733	10062	1270	5043	2036
DDX3X	23139	9617	17061	3719	21633	2050	32826	1988	19833	6783
DDX19BandDDX19A	2679	1119	3778	931	4309	1480	4307	1453	3713	973
DDX60L	6912	2530	9242	5198	5270	2022	11091	3992	5928	2756
DEGS1	3026	931	3217	682	2978	475	4397	285	3088	843
DENND5A	15913	4626	13022	2548	15064	4570	21882	3972	15389	3892
DHX40	1683	728	1651	120	2022	424	2677	254	1783	556
DMXL2	6338	2435	4960	1513	4926	1774	10167	1770	4424	854
DNAJB6	6980	1936	6578	1614	7572	1457	9161	1668	6975	1132
DNTTIP1	2449	672	2399	685	2663	818	3154	165	2510	374
DPEP2andDPEP3	14656	5382	11680	2484	17915	4106	19913	3984	15596	3425
DPY30andMEMO1	1517	366	1734	563	1831	558	2350	152	1318	244
DPYD	5956	2685	5701	1090	5237	2686	10758	610	5543	2114
DTX3L	6856	1730	9000	4346	6423	719	11032	2847	6763	2132
DUSP22	3932	1439	3650	574	5234	1179	6050	941	4022	1083
DYNC1LI1	2911	1272	2295	377	3031	446	4372	446	2738	751
DYNC1LI2	3451	1121	2988	825	3559	935	5366	695	2851	1032
DYX1C1andCCPG1	10670	2984	8865	1396	9284	3011	14600	1542	8440	1659
EAPP	1265	384	1434	397	1759	874	1895	107	1249	278
ECHDC1	1765	503	2005	969	1665	480	3132	531	1397	335
ECHDC2	2252	570	3014	1348	2997	1377	3350	1279	2764	887
EGLN1	8944	3457	6538	1635	7818	2295	13555	3221	8512	3161
EIF2AK2	5404	3824	5212	2379	3316	279	6095	1453	2869	1037
EIF2S1	1169	317	1581	543	1644	519	2029	465	1169	262
ELP2	1483	634	2065	573	2592	1514	2845	1109	1855	750
EMB	9601	2544	9840	2082	8611	626	14636	1004	8355	2235
EPHB4	2223	697	1377	675	2129	963	1731	448	2220	557
ERAP1	8713	2973	9957	1780	12030	1740	16373	2948	9543	1759
ERBB2IP	6815	2425	5738	961	6622	886	10090	747	6016	1537
ERN1	4382	1714	3787	1159	4965	1728	7309	1840	4844	1721
ETNK1	2042	753	2406	1432	2407	901	4007	817	1835	719
FAM45A	3412	1020	3250	453	3391	743	4884	808	2901	805
FAM111BandFAM111A	4627	1440	5204	2735	5584	2568	7335	1453	3893	1186
FAM118A	1404	474	3619	4483	3284	2922	11737	9061	1373	189
FAM198B	3287	931	2792	702	2275	924	5879	2203	2376	790
FBXL3	3334	1181	3062	641	3845	1187	5401	233	3078	818
FBXL5	21770	7075	17989	4663	17281	5139	30766	4975	16645	5932
FCER1A	659	362	789	540	660	439	2643	1657	519	191
FKBP1AandSDCBP2	15848	5269	16454	3166	16541	5886	22179	2942	18347	4985
FNTA	2573	892	2778	801	3254	1138	4333	822	2566	671
GALNT1	2711	731	2678	437	2899	235	4368	408	2480	480
GBP4andGBP7andGBP2	19307	1526	36610	21937	21192	7897	38993	18687	23579	4658
GCA	28956	13098	18438	5801	18391	7008	41971	15709	22966	14383
GGNBP2	3886	1239	3834	820	4634	894	5702	759	3918	1131
GHITM	7474	2165	7955	1525	8442	1292	11420	1152	7830	1865

GIT2	10394	3096	10201	2884	11512	2071	13652	612	10191	1469
GLRX	4326	2086	4236	2629	3112	1523	6009	660	2597	612
GLUD1	4225	1624	5157	930	5349	674	7531	1594	4645	1124
GMCL1	1709	680	1490	213	1751	287	2623	270	1638	368
GPX1	25910	8142	26312	5486	20138	6592	18492	5004	17160	1967
GSTK1	10735	2613	16442	5890	13080	4798	16685	2878	12857	2264
GSTO1	1905	630	2451	1523	2109	592	3339	254	1690	543
GTF2I	22811	9525	19516	3865	24787	1670	30502	1580	21308	4106
GZMA	1428	617	1547	795	1271	996	2937	1502	1341	810
H2AFZ	4191	1912	4127	1367	4287	626	6882	830	3941	1736
H3F3B	45026	15747	39713	6389	45989	12777	62670	7759	47120	17210
HBP1	7229	2864	6225	954	7768	1385	11122	485	6879	2067
HDC	614	503	239	176	302	216	1764	1764	512	442
HERC3	6097	2015	5621	1398	5807	308	9502	407	5537	1683
HERC5	1263	504	2804	1990	943	266	2549	1396	812	205
hetira	440	191	356	127	421	139	519	98	342	85
HEXB	4403	1556	4856	893	4672	1579	6884	740	3898	679
HIPK3	10559	4494	7184	1405	9652	1000	16238	3023	8418	2909
HLA-DMAandHLA-DMB	10847	5024	17619	3656	30701	24733	19045	9143	13244	4586
HLA-DQA1	2035	1739	6535	3069	5228	3448	3523	2326	4392	1705
HLA-DRB1	15565	12071	32491	5312	35195	14171	26720	11329	22321	10115
HMGB1	8794	2608	10135	4442	11212	5369	14457	2137	7295	1388
HMGCLandGALE	1244	533	1641	289	1781	365	2186	483	1569	236
HMGN4	3809	1251	4579	1573	4919	2042	6220	591	3281	651
HNRNPA2B1	23087	7149	26293	5936	30117	8683	35822	7446	23342	5973
HNRNPAB	2416	895	3146	736	3293	748	3643	946	2973	784
HNRNPH3	8205	2813	7286	1114	9637	2994	11361	1264	7074	2226
HP1BP3	9067	2995	9108	1461	11178	3504	13755	3532	9155	2295
HSD17B4andFAM170A	2886	1175	3245	619	4374	1545	4706	1285	3122	1048
HSD17B11andHSD17B13	11482	4371	9698	2433	9972	2427	15396	2073	10013	4339
HSPC157andCDC42	18664	5250	19322	2821	22369	3268	29622	2747	19973	3806
IDH1	1962	565	2285	667	2270	515	3527	448	1902	452
IFIH1	1579	495	2928	1943	1802	296	3149	1070	1388	401
IFNAR1	7051	2187	6421	2004	7847	374	13900	9175	7881	3572
IFNGR1	13233	5731	11001	3414	10557	3090	19391	2723	9607	3061
IFRD1andC7orf53	6848	3958	3649	995	4199	1070	7254	1335	3849	1244
IGFBP7	890	154	1088	180	1024	324	1464	161	1073	192
IKZF1	15488	5249	16952	3347	19972	3889	22420	3410	18264	4411
ING4	1558	477	1794	442	1877	657	2364	292	1539	306
IPMK	1096	420	728	194	1034	258	2010	791	898	294
IQGAP2	5359	2087	4799	779	5177	1850	9694	642	5150	2378
ITGA4	7230	2523	9755	2452	7895	2840	14878	4886	8546	2066
JAK2	3122	1457	3974	2110	3731	890	6215	1111	2592	607
JMJD1C	8720	2885	7092	1945	10897	4177	14207	2528	8591	3102
KIAA1033	5652	1828	4782	1738	4983	1192	8245	547	4096	1219
kihire	9478	5748	33285	20787	21896	13674	6453	2899	8470	9197
KLF13	17300	6669	16841	2472	20010	2230	24899	4211	19779	4266
LACTB	1209	449	1629	357	1270	382	2434	453	1306	219
LAPTM4A	4501	1388	4670	1136	4668	628	7062	269	3981	1159
LEMD3	1250	460	1281	276	1961	1355	2136	298	1343	397
LILRA3	4183	3643	3514	1071	4204	1906	3249	1429	6917	1446
LMBRD1	3853	1491	3250	486	4152	1117	5414	299	3623	1115
LMO4	1314	408	1467	271	1672	582	2112	360	1368	247
LOC146880	5278	2488	3378	701	3552	714	6145	448	3356	785
LOC728054	25032	11984	18851	8800	14635	5504	13819	3575	15800	5738
LOC100093631	24151	9679	19453	3927	25055	1334	30627	1522	22154	3882
LOC100132062	141377	79504	101358	69983	79349	38237	75394	15143	93510	15674
LOC100288778	8047	2136	8735	1839	10678	3737	11515	4154	8206	1107
LPCAT2andCAPNS2	5868	1937	5455	2276	3913	1196	8651	1594	5295	1872
LRMP	7466	2393	6577	1129	10651	6962	10681	1173	6603	1243
LRRFIP2	2227	392	2379	441	2360	279	3639	587	2356	412
LRRK2	24315	8105	20896	9649	17449	5076	36530	10282	20858	8559
LTA4H	18859	6050	17874	5823	19529	4858	24418	5488	16568	2475
LY75andCD302	14692	4675	13676	2967	13893	3305	24072	2753	13545	3153
MALAT1	15323	8482	11183	3708	15088	8741	17775	2983	10497	3584
MAN1A1	4096	1084	3299	734	3791	562	6939	3097	3322	1081
MARCH7	17352	5176	15222	3641	15498	2545	26467	2749	14277	2756
MAT2B	5903	2591	6812	2501	7300	1709	10816	1458	6523	2234
MCL1	79895	28222	63357	13186	76728	15212	110346	22876	71239	23454
MED4	1373	463	1466	378	1836	423	2308	285	1340	329

MEGF9	25285	11161	15341	4520	18661	7552	29727	8804	18854	6372
METTL9	8743	2525	8135	2077	7478	1673	10038	1271	6570	1172
MFSD1	4075	1572	4504	914	4081	918	6686	898	3785	785
MGST1	649	147	667	96	663	339	1014	60	514	186
MIAT	5163	1684	8840	2583	4921	1595	16520	8211	6179	3201
MICAandHCP5	8082	2723	9607	2543	8301	1714	12296	1574	8762	2216
MLX	4226	1089	4397	1049	4676	1252	5604	349	4245	601
MMADHC	2186	804	2213	485	2275	138	3391	111	2094	843
MOBKL1B	12107	3566	13221	2725	13568	1863	19267	1301	12147	2549
MPPE1	5011	1836	4934	792	5893	1262	7876	773	5371	1956
MRPL15	353	127	520	242	540	217	626	127	359	80
MS4A6EandMS4A7andMS4A14	3961	930	5291	2260	3274	1278	7972	1801	4624	888
MSMBandNCOA4	48739	8860	40073	5549	46877	16326	65055	5944	45401	10370
MTCH1	7923	2960	8051	1753	9777	1325	12040	1633	8287	1445
MTMR1	2212	791	2543	319	3332	1865	3918	600	2576	863
MTMR6	2653	886	2293	552	2516	117	4323	275	2455	611
MTO1	930	288	1369	450	1539	850	1643	339	1033	273
MTPNandLUZP6	12802	5694	11640	2392	14953	2261	21259	325	13147	4865
MX1	5358	1875	13859	10256	5447	1453	9965	5264	4212	1803
MYL12A	18805	5427	21660	5544	19370	4014	26766	2534	17570	5160
MYLIP	3354	1537	2962	739	3849	547	5635	329	3171	886
NAB1	1265	401	1156	347	1379	402	2315	285	1194	368
NAP1L1	9513	2403	14127	4612	15418	6563	15868	4410	11423	2312
NAPSB	3405	1074	3529	2122	10859	8597	5374	1885	3263	2056
NARS	3459	1670	4014	1049	4508	613	6460	1663	3726	783
NBPF9andNOTCH2NLandNBPF10	99175	37901	101449	18947	111791	27427	150191	19596	100802	33869
NBR2andNBR1	10130	3747	9323	1112	11479	1441	14553	1358	10898	2853
NCRNA00189andGAPDHP14andBACH1	10533	4268	9981	2564	10229	2403	17511	2756	10317	3865
NDFIP1	3639	1468	3912	1072	4292	632	6194	1048	3890	1229
NEK9	4136	1464	4665	898	5731	1725	6773	1484	4710	1606
NFE2L2	11297	3877	12498	2314	14208	2942	17583	2805	11640	3502
NR3C1	6086	2329	5507	811	6938	1134	9337	624	5892	1006
NSUN2	2556	888	3361	825	3781	1466	4323	1343	3059	904
OAS2	4327	1752	8339	4025	5288	784	7894	2297	4804	1445
OAS3	4184	1341	9847	9297	3080	1188	7335	3046	2941	978
OAZ2	11846	5076	10417	2901	12706	3722	16935	2068	12968	3716
OGFRL1	15868	6588	12645	2141	14322	4607	24840	648	15059	4658
PAFAH1B1	6250	2624	6231	680	7567	1149	9635	1562	6827	2157
PAN2andCNPY2andCS	8164	3052	10748	2369	12606	4530	13176	4102	10623	2812
PAPOLA	10057	2973	9445	1850	10444	1965	14709	633	9282	2271
PARP9	8983	2666	12169	8044	6434	921	13178	5295	6847	2425
PARP14	11039	2468	21723	15755	11221	1345	21752	7783	11354	2922
PCMTD2	5141	2069	2993	275	4754	2720	5616	706	3510	1112
PDPR	3116	692	4293	1179	6157	2152	5797	1678	3786	979
PELI1	9146	3957	5929	1313	8866	1884	12489	2927	7851	3800
PGK1	23323	6752	24548	4632	24235	6808	35386	2643	24045	4984
PHB2andSCARNA12	4453	1188	6123	2100	7503	3454	6582	1296	5324	1132
PHIPandTRNAF13P	3634	1331	2961	1197	3508	1351	5448	259	2822	1208
PJA2	8773	3201	7121	1070	8453	1940	13036	1066	7703	2551
PLCL2	3473	914	4062	645	5031	2536	5315	660	3866	954
PLDNandSQRDL	7446	2070	8285	2043	7857	1617	11060	1262	7355	1689
PLEK	21928	7524	21849	4427	20660	6819	33457	3747	24292	5022
PLEKHB2	6821	2776	7279	900	7931	1319	11087	1636	7629	1633
PLEKHM1P	8211	3102	6343	1682	6902	943	9105	931	6326	1287
PNRC1	13527	3491	13817	5304	16935	6652	20475	2456	15648	1966
PPIL3andCLK1	9712	4995	7318	3343	8969	5034	12712	2100	6056	2103
PPP1CBandSPDYA	8134	2701	7732	2356	7835	990	13027	1054	7096	2174
PPP1CC	4583	1800	5532	1625	6988	3359	7839	1185	4738	1121
PPP1R15B	5052	1647	4294	803	5077	929	6937	300	4477	1070
PPP2R5A	6754	2249	5167	552	6195	1004	10297	740	6588	3009
PPP3CB	2072	612	2360	417	2673	717	3541	759	2293	619
PPP3R1andWDR92	5446	2203	5224	802	6762	589	8572	195	6105	1748
PPP4R1	9248	4294	6365	2859	7769	2683	12019	2639	7670	2273
PPP6C	3857	1294	3989	593	4838	1114	5855	267	4299	1028
PPTC7	3632	1638	3083	474	4500	994	6092	684	3700	1449
PRCP	6268	2691	6332	911	6449	1692	9939	336	6696	2248
PRMT2	8359	2134	7877	1819	9182	1875	11832	1913	8210	1794
PRNP	2340	655	2878	690	3001	681	4372	1141	2668	643
PRPF38B	4507	1722	4140	1001	4759	1641	6475	1081	3874	1108
PSMA1andCOPB1	6476	1834	7784	2215	8483	1546	11411	1647	6793	1670

PSMB3	4836	1604	5199	1004	5033	1511	6722	902	5074	1271
PSMB8	8794	2737	11082	4278	9830	2348	14342	2842	9455	2367
PSMD6	3288	1027	3434	1049	3241	820	4686	278	2855	655
PSMD13	4331	1429	5159	933	5695	930	6820	1542	4970	973
PTGER4	2324	832	2596	808	2869	542	3788	700	2439	478
PTPRC	79846	28313	71198	13033	76236	8675	133614	18640	74929	25656
PXK	4398	2267	3420	987	4693	1861	5624	569	3558	708
RAB1A	4416	1184	4299	799	4669	422	6661	147	4174	1163
RAB6A	4101	906	4350	551	5058	1152	6260	902	4467	746
RAB8B	6342	1863	6090	1568	6022	874	10152	789	5466	1324
RAB10	6459	1598	6705	1219	6859	771	9847	372	6058	1023
RAB32	1815	551	1620	245	1714	854	2802	316	1606	650
RAD21	9760	4392	8309	577	10480	902	15420	555	9908	3302
RAF1	24508	9353	16364	3587	21433	5307	29171	2731	19990	5921
RAP1A	7164	2329	7054	1262	7608	1254	11504	920	6876	1697
RAP1B	8952	2183	9384	2018	9975	2856	14057	1422	8956	1879
RASSF3	8612	3392	6936	1680	8700	2027	12453	1656	8183	2461
RBBP4	4887	1732	6099	1288	7191	2970	8119	2039	5613	1255
RBL2	14321	4401	13123	1819	15930	3528	20583	3242	13083	3128
RECQL	2079	761	2504	1108	2519	1050	3924	736	2000	512
RFWD2	3986	1310	3634	623	3720	989	5783	679	3683	1036
RGS18	6497	3737	4306	897	4240	984	9388	2786	4218	823
RICTOR	5539	2058	4505	1338	4941	739	8852	1327	4461	2027
RILPL2	4265	968	4751	1307	5346	1185	6218	1047	4650	465
RIT1	3034	1053	2586	421	3100	576	4509	602	2771	900
RNF5	2669	902	2798	698	3456	1036	3141	556	2766	542
RNF6	1561	358	1714	313	1996	930	2403	237	1477	270
RNF13	7358	2780	6644	1869	6148	1478	10260	735	5831	1896
RNF31andIRF9	15043	3399	19783	5953	17565	4960	20346	4467	15780	2169
RNF103andVPS24	6557	2083	6548	1015	7223	942	9870	452	6309	1422
RNF141	4158	1718	3437	647	4590	987	6180	463	4149	1229
RNF145	5375	1966	4627	957	5081	572	8164	245	4768	1607
RNF213	28523	8903	39739	19789	26121	2171	44591	9838	26759	8939
ROCK1	7411	2554	5859	1102	7028	1378	10784	1517	5952	1585
RPL14.1	14682	2852	22491	9031	25788	11410	21651	5234	16898	5774
S100A6	28702	7047	26369	4541	30022	10452	35612	4259	26495	6397
SACM1L	2883	1035	3032	957	2875	315	4932	699	2660	777
SAR1AandTYSND1andAIFM2	5223	2059	6064	1225	7380	2364	8680	2014	5924	1399
SCP2	1963	959	2546	803	2431	261	3799	668	2354	701
SCPEP1	5807	1611	6327	898	5558	2395	9551	2734	5828	1545
SDHD_	3053	958	3358	858	3776	415	5147	582	3148	865
SEC22B	3814	1120	3453	726	4030	643	5745	343	3480	979
SEC61B	1527	406	1792	538	1927	365	2535	425	1545	368
SELEandSELL	116676	37605	101619	34941	98153	12081	143937	38341	101494	37106
SENP6	3690	1282	3914	1034	4630	2221	6411	1029	3585	471
SEP15	4817	1492	5300	1972	5691	852	8315	685	4870	1669
SEPT5andGP1BB	3040	1005	4414	2440	3324	1422	2197	863	3199	695
SERINC1	11918	4607	10030	2055	10612	1597	19395	1147	10002	4005
SERINC3	8791	3184	8008	1348	8653	1176	11619	433	8397	1444
SKAP2	8270	4299	7288	1101	8906	2809	13096	2550	7085	2829
SKIV2L2	1707	839	2128	621	2449	1151	3164	839	1788	419
SLA	25697	11828	19283	5564	21370	5833	30681	4487	21891	5605
SLBP	2027	869	2288	515	2586	503	3133	565	2293	379
SLC12A7	2294	1002	4791	1437	2759	1687	4488	884	5090	2172
SLC25A37	106975	30928	81688	20693	64458	18318	92739	35386	77234	32825
SLK	3653	1099	3449	604	3468	485	6077	679	3524	423
SLU7	3240	1071	2918	885	3472	1472	4334	336	2541	453
SMAP2	58584	26805	40423	13732	57032	12360	68561	7199	52014	9653
SMARCA5	3513	994	4101	1024	5174	2543	6024	1112	3692	659
SMCHD1	28074	9653	24829	9248	28299	5630	42826	6396	23204	8040
SNX2	3172	718	3509	1136	3987	1494	5201	530	2730	848
SNX6	3806	1229	4428	1344	4250	437	6648	516	4103	1431
SNX10	5057	1938	4358	1453	4831	498	8250	1910	4609	2101
SNX14	1965	535	2204	798	2342	1079	3302	530	1759	325
SP140LandSP100andHMGB1L3	13448	3624	16570	4792	19947	13622	19578	2029	13529	4069
SPATA13andC1QTNF9	7350	2599	6004	886	5413	965	9301	840	6186	1387
SPCS3	5596	1967	6340	1656	6644	1180	9793	1618	5615	1419
SPOPL	4318	1866	3080	902	3825	413	6948	1440	3307	1224
SPPL2A	1617	459	1693	924	1507	80	2826	370	1640	467
SRI	1566	428	1968	524	1966	516	2725	340	1778	348

SRP9andEPHX1	4189	1057	4792	1792	5348	1938	6656	950	3906	773
SSFA2	4310	1438	3956	781	3985	701	7148	684	3622	891
ST8SIA4	6923	2747	5684	1577	6099	1796	10680	1792	7006	2480
STAT1	14079	4139	28391	24214	14618	3641	27446	12803	14859	5136
STOM	3860	1661	4982	2019	3453	1107	6713	1406	4055	1760
STXBP3	3179	973	2816	831	2764	365	5264	444	2643	752
SURF4	6564	2032	8030	1912	8733	2315	9181	1821	7810	1603
SYTL2	609	448	625	273	691	291	1350	698	888	588
TAF1	2338	823	2314	288	3317	1570	3732	692	2516	686
TAGAP	20844	6933	21716	7188	31079	10471	32384	5403	25095	5949
TAX1BP1	7026	939	6941	2378	6866	1614	10389	927	5621	758
TBC1D2B	5428	1990	6360	1548	5834	1254	9303	1434	6002	1419
TCF25andMC1RandTUBB3	8031	2862	9584	2581	11110	2786	12115	2108	10228	2325
TCP11L2	3240	757	1773	295	2781	1019	3742	794	2204	733
TDG	914	260	957	307	1155	333	1560	268	922	210
TDP2	4293	1192	4129	841	4387	1084	6346	1121	3918	1149
TES	4484	1451	5157	935	5048	744	7587	1540	5163	1603
TGFBR2	17692	5534	15928	3112	18687	3297	24962	2296	17863	4811
TM9SF2	11278	4249	10773	1630	10781	1094	16972	1026	11298	2977
TM9SF3	4204	1434	4531	1101	4820	766	7311	987	4052	1122
TMCC1	4206	1963	3310	1283	4780	1989	6074	662	4177	793
TMCO3	1925	512	1807	418	2170	460	2824	515	1776	83
TMEM49	31262	11125	24509	5918	29890	10594	44329	9214	26738	8176
TMEM59	6838	2180	6495	2329	6587	575	10074	319	5849	1985
TMEM167B	3499	1125	3564	794	3652	557	6126	348	3572	1295
TMEM222	2329	769	2732	799	3356	1053	3229	692	2803	392
TMSB4X	109323	55418	130781	38031	122966	32173	198667	28828	118531	53001
TNFSF13B	4531	1179	5254	2916	3331	778	7693	1852	3842	1547
TNKS2	3812	1423	3967	794	4599	1180	6546	704	3921	1071
TNPO3	3995	1599	3518	742	4470	985	5275	482	3962	1079
TOPORSandDDX58	6213	1464	6545	2680	5578	1220	8766	1413	5088	1084
TOR1A	2945	823	2977	735	2897	509	3710	389	2533	544
TOR1AIP1	5643	1657	5762	992	6186	943	8205	399	5644	922
TPM3	29984	10641	32163	4713	35544	6831	45822	7140	34000	7263
TRAM1	5588	1889	7256	2122	7440	2901	9838	2005	6125	1255
TRPC4AP	9278	3247	8869	1198	9697	789	12722	1672	9584	1724
TSNAXandDISC1	2826	789	3036	672	2688	283	4743	355	2635	490
TSPAN14	11668	3615	11949	2881	11423	4454	14690	2101	12615	1783
TXNRD1	3832	1869	3892	808	4666	1485	7311	306	4355	1364
U2AF1	5909	1918	5727	1106	6521	1828	8096	499	5148	1272
UBE2B	7191	2286	4961	894	5929	1250	9303	1619	5831	2440
UBE2E3	1336	507	1506	214	1700	407	2196	323	1517	289
UBL7	3018	687	3639	533	4161	525	3463	553	3793	713
UBR2	10942	4579	8360	1530	10231	2453	15525	1701	9784	3797
UHMK1	4438	1916	4821	1932	5586	2174	8083	1458	4329	1434
USP1	1598	426	1878	584	2110	1083	2675	528	1472	354
USP15	18656	7899	13866	6773	12346	1708	24307	2637	11429	4710
USP33	2500	854	2637	735	2749	775	4212	399	2301	609
UTRN	4536	1886	5410	868	5649	956	8619	2006	5527	1328
VAMP3	4621	1444	4870	806	5529	965	7501	665	5175	1216
VCP	8700	4338	10566	2138	12279	1047	14305	2791	11791	2752
VNN2	59279	22497	34656	9655	39901	13333	67788	33581	38935	15486
VPS13C	3828	1090	4549	1250	6430	4463	7166	1850	4079	1205
WARS	9576	3978	18976	8485	12048	5314	18128	7387	14862	1832
WDFY2	4089	1050	3979	642	4342	403	6411	496	3679	951
WSB1	9054	3109	8412	3903	7715	1709	13136	1495	7088	1573
yakeme	4771	1547	4805	800	6216	2055	7763	542	4804	1176
YIPF4	1724	594	1477	482	1490	220	2598	210	1528	623
YWHAE	5410	1600	6877	1540	7849	2199	8366	1090	5983	1302
ZBED5andEIF4G2andSNORD97	28345	9302	30706	4366	38153	11908	44612	3849	30313	8058
ZCCHC6	10222	3188	8649	1840	10028	2335	15060	3150	9526	2727
ZEB2andGTDC1	8125	2429	8536	1109	9234	763	14406	1901	8379	1088
ZFAND5	7941	1776	7687	1457	7853	1080	12997	1421	7825	995
ZFP91-CNTF	2951	1163	3479	429	4048	1215	5063	774	3422	780
ZNF516	6382	2319	4534	1373	4956	1804	7568	854	5085	685
ZNF592	7674	2309	7398	2533	9293	1305	9523	1702	8615	1644
