# Supplementary material for: Intracerebral Hemorrhage and Ischemic Stroke of Different Etiologies Have Distinct Alternatively Spliced mRNA Profiles in the Blood: a Pilot RNA-seq Study
Source: Transl Stroke Res. 2015 May 22;6(4):284–9. doi: 10.1007/s12975-015-0407-9 (PMC4485700; doi:10.1007/s12975-015-0407-9)
Supplement: Supplementary file 7 — The 412 genes with DAS among Large Vessel Ischemic Stroke (IS), Cardioembolic IS, Lacunar IS and ICH and Controls (ANOVA, FDR corrected p < 0.05). (PDF 72 kb) [file 12975_2015_407_MOESM3_ESM.pdf]

SUPPLEMENTARY TABLE 3. Genes (412) with Differential Alternative Splicing Among Large Vessel Ischemic Stroke (IS), Cardioembolic IS, Lacunar IS, Intracerebral Hemorrhage and Control Subjects			
**Gene Symbol**	**Gene Name**	**alt-splicing (Dx) p-Value**	**FDR p-Value**
MARCH7	membrane-associated ring finger (C3HC4) 7	1.18E-05	9.07E-04
SEPT15	septin 5	3.38E-05	2.16E-03
ABCA7	ATP-binding cassette, sub-family A (ABC1), member 7	3.37E-08	7.73E-06
ACSL4/KCNE1L	acyl-CoA synthetase long-chain family member 4/KCNE1-like	6.77E-05	3.66E-03
ACTR2	ARP2 actin-related protein 2 homolog (yeast)	5.76E-20	2.57E-16
ACTR3	ARP3 actin-related protein 3 homolog (yeast)	1.29E-03	3.34E-02
ADCK2/NDUFB2	aarF domain containing kinase 2/NADH dehydrogenase (ubiquinone) 1 beta subcomplex, 2, 8kDa	6.16E-05	3.42E-03
ADCY7	adenylate cyclase 7	6.29E-09	1.76E-06
ADD3	adducin 3 (gamma)	1.14E-05	8.90E-04
ADSS/TGIF2P1	adenylosuccinate synthase/TGFB-induced factor homeobox 2 pseudogene 1	4.63E-05	2.67E-03
AKAP8	A kinase (PRKA) anchor protein 8	1.29E-03	3.34E-02
ANAPC13	anaphase promoting complex subunit 13	1.50E-03	3.70E-02
ANKRD12	ankyrin repeat domain 12	3.96E-06	3.97E-04
ANKRD13A	ankyrin repeat domain 13A	7.49E-05	3.95E-03
ANXA1	annexin A1	8.66E-08	1.65E-05
ANXA7	annexin A7	8.70E-06	7.20E-04
AP1S2	adaptor-related protein complex 1, sigma 2 subunit pseudogene; adaptor-related protein complex 1, sigma 2 subunit	1.11E-03	2.93E-02
APAF1	apoptotic peptidase activating factor 1	1.80E-03	4.20E-02
APH1A	anterior pharynx defective 1 homolog A (C. elegans)	1.32E-03	3.40E-02
APIP	APAF1 interacting protein; similar to APAF1 interacting protein	1.35E-03	3.45E-02
APOBEC3A/APOBEC3B	apolipoprotein B mRNA editing enzyme, catalytic polypeptide-like 3A/apolipoprotein B mRNA editing enzyme, catalytic polypeptide-like 3B	5.87E-04	1.81E-02
ARCN1	archain 1	2.53E-04	9.85E-03
ARFIP1/FHDC1	ADP-ribosylation factor interacting protein 1/FH2 domain containing 1	1.69E-05	1.24E-03

ARID4B/RBM34	AT rich interactive domain 4B (RBP1-like)/RNA binding motif protein 34	4.36E-05	2.56E-03
ARL6IP5	ADP-ribosylation-like factor 6 interacting protein 5	2.89E-05	1.91E-03
ARNTL	aryl hydrocarbon receptor nuclear translocator-like	1.83E-05	1.32E-03
ARPC3/ANAPC7	actin related protein 2/3 complex, subunit 3, 21kDa/anaphase promoting complex subunit 7	5.80E-11	3.45E-08
ARPC4/TTLL3	actin related protein 2/3 complex, subunit 4, 20kDa/tubulin tyrosine ligase-like family member 3	3.14E-11	2.16E-08
ARPC5L	actin related protein 2/3 complex, subunit 5-like	2.44E-04	9.56E-03
ATM	similar to Serine-protein kinase ATM (Ataxia telangiectasia mutated) (A-T, mutated); ataxia telangiectasia mutated	6.24E-05	3.42E-03
ATP2B4	ATPase, Ca++ transporting, plasma membrane 4	2.42E-04	9.54E-03
ATP5B/SNORD59A/SNORD59B	ATP synthase, H+ transporting, mitochondrial F1 complex, beta polypeptide/small nucleolar RNA, C/D box 59A/small nucleolar RNA, C/D box 59B	3.76E-10	1.46E-07
ATP6V1G2/BAT1(DDX39B)/SNORD84	ATPase, H+ transporting, lysosomal 13kDa, V1 subunit G2/DEAD (Asp-Glu-Ala-Asp) box polypeptide 39B/ small nucleolar RNA, C/D box 84	2.39E-06	2.51E-04
ATXN1L/KIAA0174(IST1)	ataxin 1-like/increased sodium tolerance 1 homolog (yeast)	8.77E-05	4.38E-03
AZIN1	antizyme inhibitor 1	8.54E-04	2.38E-02
baboy		5.62E-04	1.76E-02
BAZ1A	bromodomain adjacent to zinc finger domain, 1A	7.63E-04	2.21E-02
BAZ2B	bromodomain adjacent to zinc finger domain, 2B	4.42E-04	1.49E-02
BTN2A2/BTN3A1	butyrophilin, subfamily 2, member A2/butyrophilin, subfamily 3, member A1	3.13E-06	3.21E-04
C11orf73	chromosome 11 open reading frame 73	1.71E-03	4.08E-02
C15orf29	chromosome 15 open reading frame 29	5.37E-08	1.17E-05
C1orf59	chromosome 1 open reading frame 59	5.42E-05	3.03E-03
C1orf63	chromosome 1 open reading frame 63	1.10E-12	1.40E-09
C5orf15	chromosome 5 open reading frame 15	5.26E-04	1.67E-02
C6orf62	chromosome 6 open reading frame 62	1.51E-04	6.62E-03
C7orf27	chromosome 7 open reading frame 27	7.25E-04	2.11E-02
C9orf114	chromosome 9 open reading frame 114	2.31E-04	9.15E-03
C9orf72	chromosome 9 open reading frame 72	5.43E-04	1.71E-02
CAB39	calcium binding protein 39	9.34E-05	4.56E-03

CALM1	calmodulin 3 (phosphorylase kinase, delta); calmodulin 2 (phosphorylase kinase, delta); calmodulin 1 (phosphorylase kinase, delta)	2.24E-07	3.77E-05
CALM2/C2orf61	calmodulin 2 (phosphorylase kinase, delta)/chromosome 2 open reading frame 61	3.70E-12	3.00E-09
CAPZA2	capping protein (actin filament) muscle Z-line, alpha 2	6.35E-04	1.91E-02
CARD8	caspase recruitment domain family, member 8	5.51E-08	1.17E-05
CBARA1	calcium binding atopy-related autoantigen 1	2.87E-04	1.08E-02
CCAR1	cell division cycle and apoptosis regulator 1	1.61E-05	1.19E-03
CCNDBP1	cyclin D-type binding-protein 1	3.17E-10	1.29E-07
CCNY	cyclin Y	1.68E-04	7.13E-03
CCT8	similar to chaperonin containing TCP1, subunit 8 (theta); chaperonin containing TCP1, subunit 8 (theta)	2.19E-03	4.80E-02
CD164	CD164 molecule, sialomucin	1.82E-05	1.32E-03
CD244	CD244 molecule, natural killer cell receptor 2B4	6.82E-04	2.03E-02
CD300E	CD300e molecule	1.05E-04	4.97E-03
CD36	CD36 molecule (thrombospondin receptor)	2.83E-07	4.60E-05
CD46	CD46 molecule, complement regulatory protein	4.46E-06	4.29E-04
CD47	CD47 molecule	4.97E-15	9.66E-12
CD53	CD53 molecule	1.52E-04	6.62E-03
CD58	CD58 molecule	2.07E-04	8.41E-03
CD74	CD74 molecule, major histocompatibility complex, class II invariant chain	6.89E-04	2.03E-02
CD86	CD86 molecule	1.60E-03	3.88E-02
CDC42SE1	CDC42 small effector 1	1.70E-06	1.97E-04
CDC42SE2	CDC42 small effector 2	3.58E-04	1.26E-02
CDKL3/PPP2CA	cyclin-dependent kinase-like 3/protein phosphatase 2, catalytic subunit, alpha isozyme	3.66E-05	2.26E-03
CDKN1C	cyclin-dependent kinase inhibitor 1C (p57, Kip2)	2.33E-05	1.59E-03
CECR1	cat eye syndrome chromosome region, candidate 1	6.88E-06	5.97E-04
CELF2	CUG triplet repeat, RNA binding protein 2	3.17E-04	1.14E-02
CFLAR/RNU7-45P	CASP8 and FADD-like apoptosis regulator/RNA, U7 small nuclear 45 pseudogene	1.42E-08	3.64E-06
CGGBP1	CGG triplet repeat binding protein 1	7.87E-05	4.06E-03
CHMP2B	chromatin modifying protein 2B	1.42E-03	3.57E-02
CLDND1	claudin domain containing 1	2.13E-06	2.32E-04
CLEC7A	C-type lectin domain family 7, member A	8.25E-10	3.02E-07

CLTC	clathrin, heavy chain (Hc)	2.74E-04	1.04E-02
CNIH	cornichon homolog (Drosophila)	1.84E-04	7.55E-03
CNOT6L	CCR4-NOT transcription complex, subunit 6-like	6.04E-04	1.85E-02
CNOT7	CCR4-NOT transcription complex, subunit 7	2.15E-03	4.75E-02
CNOT8	CCR4-NOT transcription complex, subunit 8	3.00E-04	1.10E-02
COMMD2	canopy 2 homolog (zebrafish)	1.41E-03	3.55E-02
CRKL	COMM domain containing 2	1.14E-03	3.00E-02
CSGALNACT2	chondroitin sulfate N-acetylgalactosaminyltransferase 2; novel protein similar to chondroitin sulfate GalNAcT-2 (GALNACT-2)	3.60E-05	2.26E-03
CTDSP2	similar to hCG2013701; CTD (carboxy-terminal domain, RNA polymerase II, polypeptide A) small phosphatase 2	5.61E-04	1.76E-02
CTSS	cathepsin S	1.46E-06	1.76E-04
CYBB	cytochrome b-245, beta polypeptide	7.15E-09	1.94E-06
CYBRD1	cytochrome b reductase 1	2.31E-05	1.59E-03
CYLD	cylindromatosis (turban tumor syndrome)	1.37E-03	3.47E-02
DAP3	death associated protein 3	2.13E-03	4.74E-02
DCP2	DCP2 decapping enzyme homolog (S. cerevisiae)	3.88E-07	5.69E-05
DDX19B/DDX19A	DEAD (Asp-Glu-Ala-Asp) box polypeptide 19B/DEAD (Asp-Glu-Ala-Asp) box polypeptide 19A	3.10E-04	1.13E-02
DDX3X	DEAD (Asp-Glu-Ala-Asp) box polypeptide 3, X-linked	1.37E-12	1.53E-09
DDX60L	DEAD (Asp-Glu-Ala-Asp) box polypeptide 60-like	4.35E-06	4.27E-04
DEGS1	degenerative spermatocyte homolog 1, lipid desaturase (Drosophila)	1.25E-04	5.67E-03
DENND5A	DENN/MADD domain containing 5A	3.64E-07	5.45E-05
DHX40	similar to DEAH (Asp-Glu-Ala-His) box polypeptide 40; DEAH (Asp-Glu-Ala-His) box polypeptide 40	7.11E-04	2.08E-02
DMXL2	Dmx-like 2	7.29E-12	5.43E-09
DNAJB6	DnaJ (Hsp40) homolog, subfamily B, member 6	1.20E-04	5.52E-03
DNTTIP1	deoxynucleotidyltransferase, terminal, interacting protein 1	1.88E-03	4.29E-02
DPEP2/DPEP3	dipeptidase 2/dipeptidase 3	5.43E-05	3.03E-03
DPY30/MEMO1	dpy-30 homolog (C. elegans)/mediator of cell motility 1	2.26E-03	4.91E-02
DPYD	dihydropyrimidine dehydrogenase	8.13E-08	1.61E-05
DTX3L	deltex 3-like (Drosophila)	3.95E-04	1.37E-02

DUSP22	similar to mitogen-activated protein kinase phosphatase x; dual specificity phosphatase 22	7.74E-04	2.21E-02
DYNC1LI1	dynein, cytoplasmic 1, light intermediate chain 1	7.00E-04	2.06E-02
DYNC1LI2	dynein, cytoplasmic 1, light intermediate chain 2	3.68E-05	2.26E-03
DYX1C1/CCPG1	dyslexia susceptibility 1 candidate 1/cell cycle progression 1	7.61E-05	3.95E-03
EAPP	E2F-associated phosphoprotein	5.17E-04	1.67E-02
ECHDC1	enoyl Coenzyme A hydratase domain containing 1	7.45E-06	6.34E-04
ECHDC2	enoyl Coenzyme A hydratase domain containing 2	9.29E-04	2.55E-02
EGLN1	egl nine homolog 1 (C. elegans)	2.12E-04	8.51E-03
EIF2AK2	eukaryotic translation initiation factor 2-alpha kinase 2	1.63E-03	3.91E-02
EIF2S1	eukaryotic translation initiation factor 2, subunit 1 alpha, 35kDa	4.13E-04	1.42E-02
ELP2	elongation protein 2 homolog (S. cerevisiae)	1.01E-05	8.06E-04
EMB	embigin homolog (mouse)	1.03E-10	5.41E-08
EPHB4	EPH receptor B4	1.48E-03	3.67E-02
ERAP1	endoplasmic reticulum aminopeptidase 1	8.18E-05	4.13E-03
ERBB2IP	erbb2 interacting protein	6.97E-05	3.73E-03
ERN1	endoplasmic reticulum to nucleus signaling 1	1.70E-03	4.07E-02
ETNK1	ethanolamine kinase 1	1.83E-03	4.23E-02
FAM111B/FAM111A	family with sequence similarity 111, member B/family with sequence similarity 111, member A	1.78E-03	4.16E-02
FAM118A	family with sequence similarity 118, member A	9.23E-10	3.13E-07
FAM198B	chromosome 4 open reading frame 18	2.90E-04	1.08E-02
FAM45A	family with sequence similarity 45, member A	2.11E-04	8.51E-03
FBXL3	F-box and leucine-rich repeat protein 3	2.01E-04	8.22E-03
FBXL5	F-box and leucine-rich repeat protein 5	1.40E-06	1.71E-04
FCER1A	Fc fragment of IgE, high affinity I, receptor for; alpha polypeptide	6.21E-07	8.53E-05
FKBP1A/SDCBP2	FK506 binding protein 1A, 12kDa/syndecan binding protein (syntenin) 2	1.09E-03	2.89E-02
FNTA	farnesyltransferase, CAAX box, alpha	2.84E-08	6.86E-06
GALNT1	UDP-N-acetyl-alpha-D-galactosamine:polypeptide N-acetylgalactosaminyltransferase 13 (GalNAc-T13); UDP-N-acetyl-alpha-D-galactosamine:polypeptide N-acetylgalactosaminyltransferase 1 (GalNAc-T1)	3.80E-09	1.13E-06

GBP4/GBP7/GBP2	guanylate binding protein 4/guanylate binding protein 7/guanylate binding protein 2, interferon-inducible	3.67E-05	2.26E-03
GCA	grancalcin, EF-hand calcium binding protein	2.92E-07	4.66E-05
GGNBP2	gametogenetin binding protein 2	1.12E-04	5.26E-03
GHITM	growth hormone inducible transmembrane protein	9.44E-07	1.22E-04
GIT2	G protein-coupled receptor kinase interacting ArfGAP 2	5.73E-04	1.78E-02
GLRX	glutaredoxin (thioltransferase)	5.22E-04	1.67E-02
GLUD1	glutamate dehydrogenase 1	4.22E-05	2.52E-03
GMCL1	germ cell-less homolog 1 (Drosophila)-like; germ cell-less homolog 1 (Drosophila)	4.91E-04	1.62E-02
GPX1	glutathione peroxidase 1	1.77E-03	4.15E-02
GSTK1	glutathione S-transferase kappa 1	1.77E-04	7.34E-03
GSTO1	glutathione S-transferase omega 1	4.25E-04	1.44E-02
GTF2I	general transcription factor II, i; general transcription factor II, i, pseudogene	1.39E-05	1.04E-03
GZMA	granzyme A (granzyme 1, cytotoxic T-lymphocyte-associated serine esterase 3)	1.27E-03	3.31E-02
H2AFZ	H2A histone family, member Z	8.03E-04	2.26E-02
H3F3B	H3 histone, family 3B (H3.3B); H3 histone, family 3A pseudogene; H3 histone, family 3A; similar to H3 histone, family 3B; similar to histone H3.3B	1.90E-03	4.32E-02
HBP1	HMG-box transcription factor 1	7.69E-04	2.21E-02
HDC	histidine decarboxylase	1.26E-03	3.28E-02
HERC3	hect domain and RLD 3	4.70E-07	6.67E-05
HERC5	hect domain and RLD 5	1.03E-05	8.14E-04
hetira		7.84E-04	2.22E-02
HEXB	hexosaminidase B (beta polypeptide)	3.00E-04	1.10E-02
HIPK3	homeodomain interacting protein kinase 3	2.01E-10	9.45E-08
HLA-DMA/HLA-DMB	major histocompatibility complex, class II, DM alpha/major histocompatibility complex, class II, DM beta	5.32E-05	3.01E-03
HLA-DQA1	similar to hCG2042724; similar to HLA class II histocompatibility antigen, DQ(1) alpha chain precursor (DC-4 alpha chain); major histocompatibility complex, class II, DQ alpha 1	1.89E-03	4.31E-02
HLA-DRB1	major histocompatibility complex, class II, DR beta 4; major histocompatibility complex, class II, DR beta 1	2.35E-06	2.49E-04
HMGB1	high-mobility group box 1; high-mobility group box 1-like 10	1.45E-03	3.64E-02

HMGCL/GALE	3-hydroxymethyl-3-methylglutaryl-CoA lyase/UDP-galactose-4-epimerase	1.02E-03	2.73E-02
HMGN4	high mobility group nucleosomal binding domain 4	7.64E-06	6.44E-04
HNRNPA2B1	heterogeneous nuclear ribonucleoprotein A2/B1	5.52E-06	4.93E-04
HNRNPAB	heterogeneous nuclear ribonucleoprotein A/B	3.04E-04	1.11E-02
HNRNPH3	heterogeneous nuclear ribonucleoprotein H3 (2H9)	2.16E-06	2.32E-04
HP1BP3	heterochromatin protein 1, binding protein 3	1.62E-03	3.90E-02
HSD17B11/HSD17B13	hydroxysteroid (17-beta) dehydrogenase 11/hydroxysteroid (17-beta) dehydrogenase 13	6.11E-04	1.86E-02
HSD17B4/FAM170A	hydroxysteroid (17-beta) dehydrogenase 4/family with sequence similarity 170, member A	2.19E-05	1.52E-03
HSPC157 (LINC00339)/CDC42	long intergenic non-protein coding RNA 339 /cell division cycle 42	1.01E-03	2.72E-02
IDH1	isocitrate dehydrogenase 1 (NADP+), soluble	7.39E-06	6.34E-04
IFIH1	interferon induced with helicase C domain 1	5.00E-04	1.65E-02
IFNAR1	interferon (alpha, beta and omega) receptor 1	3.13E-04	1.13E-02
IFNGR1	interferon gamma receptor 1	3.75E-11	2.39E-08
IFRD1/C7orf53 (LSMEM1)	interferon-related developmental regulator 1/leucine-rich single-pass membrane protein 1	8.87E-04	2.44E-02
IGFBP7	insulin-like growth factor binding protein 7	3.17E-04	1.14E-02
IKZF1	IKAROS family zinc finger 1 (Ikaros)	1.72E-03	4.10E-02
ING4	inhibitor of growth family, member 4	1.09E-06	1.39E-04
IPMK	inositol polyphosphate multikinase	5.25E-04	1.67E-02
IQGAP2	IQ motif containing GTPase activating protein 2	8.51E-04	2.38E-02
ITGA4	integrin, alpha 4 (antigen CD49D, alpha 4 subunit of VLA-4 receptor)	5.23E-06	4.77E-04
JAK2	Janus kinase 2	5.67E-07	7.92E-05
JMJD1C	jumonji domain containing 1C	1.42E-05	1.05E-03
KIAA1033	KIAA1033	7.58E-05	3.95E-03
kihire		2.02E-03	4.56E-02
KLF13	Kruppel-like factor 13	1.75E-03	4.14E-02
LACTB	lactamase, beta	2.22E-07	3.77E-05
LAPTM4A	lysosomal protein transmembrane 4 alpha	1.81E-03	4.20E-02
LEMD3	LEM domain containing 3	4.59E-04	1.54E-02
LILRA3	leukocyte immunoglobulin-like receptor, subfamily A (without TM domain), member 3	6.01E-04	1.84E-02
LMBRD1	LMBR1 domain containing 1	1.82E-03	4.21E-02

LMO4	LIM domain only 4	1.07E-04	5.08E-03
LOC100093631	general transcription factor II, i; general transcription factor II, i, pseudogene	1.74E-04	7.26E-03
LOC100132062	hypothetical LOC100132062	2.07E-03	4.63E-02
LOC100288778	similar to WAS protein family homolog 1	1.53E-04	6.62E-03
LOC146880	hypothetical LOC146880	5.27E-04	1.67E-02
LOC728054	hypothetical LOC728054	1.76E-06	2.01E-04
LPCAT2/CAPNS2	lysophosphatidylcholine acyltransferase 2/calpain, small subunit 2	1.01E-04	4.87E-03
LRMP	lymphoid-restricted membrane protein	8.68E-04	2.42E-02
LRRFIP2	leucine rich repeat (in FLII) interacting protein 2	6.70E-07	9.07E-05
LRRK2	leucine-rich repeat kinase 2	2.43E-05	1.64E-03
LTA4H	leukotriene A4 hydrolase	8.88E-05	4.41E-03
LY75/CD302	lymphocyte antigen 75/CD302 molecule	6.74E-18	2.01E-14
MALAT1	metastasis associated lung adenocarcinoma transcript 1 (non-protein coding)	1.73E-04	7.26E-03
MAN1A1	mannosidase, alpha, class 1A, member 1	6.62E-05	3.61E-03
MAT2B	methionine adenosyltransferase II, beta	1.16E-04	5.41E-03
MCL1	myeloid cell leukemia sequence 1 (BCL2-related)	2.05E-03	4.60E-02
MED4	mediator complex subunit 4	6.30E-06	5.52E-04
MEGF9	multiple EGF-like-domains 9	1.54E-03	3.77E-02
METTL9	methyltransferase like 9	7.05E-08	1.46E-05
MFSD1	major facilitator superfamily domain containing 1	2.99E-05	1.95E-03
MGST1	microsomal glutathione S-transferase 1	3.86E-05	2.33E-03
MIAT	myocardial infarction associated transcript (non-protein coding)	9.16E-08	1.70E-05
MICA/HCP5	MHC class I polypeptide-related sequence A/HLA complex P5 (non-protein coding)	2.15E-05	1.50E-03
MLX	MAX-like protein X	2.24E-03	4.89E-02
MMADHC	methylmalonic aciduria (cobalamin deficiency) cblD type, with homocystinuria	1.65E-04	7.08E-03
MOBKL1B	MOB1, Mps One Binder kinase activator-like 1B (yeast)	7.62E-11	4.25E-08
MPPE1	metallophosphoesterase 1	4.29E-13	6.39E-10
MRPL15	mitochondrial ribosomal protein L15	6.55E-04	1.96E-02
MS4A6E/MS4A7/MS4A14	membrane-spanning 4-domains, subfamily A, member 6E/membrane-spanning 4-domains, subfamily A, member 7/membrane-spanning 4-domains, subfamily A, member 14	1.84E-04	7.55E-03
MSMB/NCOA4	microseminoprotein, beta-/nuclear receptor coactivator 4	9.13E-06	7.48E-04

MTCH1	mitochondrial carrier homolog 1 (C. elegans)	1.04E-03	2.76E-02
MTMR1	myotubularin related protein 1	7.73E-04	2.21E-02
MTMR6	myotubularin related protein 6	5.13E-04	1.67E-02
MTO1	mitochondrial translation optimization 1 homolog (S. cerevisiae)	3.83E-05	2.33E-03
MTPN/LUZP6	myotrophin/leucine zipper protein 6	5.15E-04	1.67E-02
MX1	myxovirus (influenza virus) resistance 1, interferon-inducible protein p78 (mouse)	3.36E-12	3.00E-09
MYL12A	myosin, light chain 12A, regulatory, non-sarcomeric	1.66E-06	1.95E-04
MYLIP	myosin regulatory light chain interacting protein	1.61E-03	3.88E-02
NAB1	NGFI-A binding protein 1 (EGR1 binding protein 1)	7.51E-05	3.95E-03
NAP1L1	nucleosome assembly protein 1-like 1	1.58E-03	3.84E-02
NAPSB	napsin B aspartic peptidase pseudogene	5.81E-06	5.14E-04
NARS	asparaginyl-tRNA synthetase	1.26E-04	5.70E-03
NBPF9/NOTCH2NL/NBPF10	neuroblastoma breakpoint family, member 9/notch 2 N-terminal like/neuroblastoma breakpoint family, member 10	7.79E-04	2.21E-02
NBR2/NBR1	neighbor of BRCA1 gene 2 (non-protein coding)/neighbor of BRCA1 gene 1	1.77E-03	4.15E-02
NCRNA00189 (LINC00189)/GAPDHP14/BACH1	long intergenic non-protein coding RNA 189/glyceraldehyde-3-phosphate dehydrogenase pseudogene 14/BTB and CNC homology 1, basic leucine zipper transcription factor 1	4.89E-06	4.50E-04
NDFIP1	Nedd4 family interacting protein 1	2.26E-03	4.91E-02
NEK9	NIMA (never in mitosis gene a)- related kinase 9	2.94E-04	1.09E-02
NFE2L2	nuclear factor (erythroid-derived 2)-like 2	3.18E-04	1.14E-02
NR3C1	nuclear receptor subfamily 3, group C, member 1 (glucocorticoid receptor)	3.02E-06	3.14E-04
NSUN2	NOL1/NOP2/Sun domain family, member 2	8.40E-06	7.02E-04
OAS2	2'-5'-oligoadenylate synthetase 2, 69/71kDa	2.12E-04	8.51E-03
OAS3	2'-5'-oligoadenylate synthetase 3, 100kDa	1.07E-03	2.84E-02
OAZ2	ornithine decarboxylase antizyme 2	7.72E-04	2.21E-02
OGFRL1	opioid growth factor receptor-like 1	4.09E-04	1.42E-02
PAFAH1B1	platelet-activating factor acetylhydrolase, isoform Ib, subunit 1 (45kDa)	4.60E-05	2.67E-03
PAN2/CNPY2/CS	PAN2 poly(A) specific ribonuclease subunit/canopy FGF signaling regulator 2/ citrate synthase	2.94E-05	1.93E-03
PAPOLA	poly(A) polymerase alpha	1.41E-03	3.55E-02

PARP14	poly (ADP-ribose) polymerase family, member 14	3.54E-08	7.91E-06
PARP9	poly (ADP-ribose) polymerase family, member 9	1.25E-04	5.67E-03
PCMTD2	protein-L-isoaspartate (D-aspartate) O-methyltransferase domain containing 2	9.19E-05	4.51E-03
PDPR	pyruvate dehydrogenase phosphatase regulatory subunit	3.87E-04	1.35E-02
PELI1	pellino homolog 1 (Drosophila)	2.46E-07	4.07E-05
PGK1	phosphoglycerate kinase 1	2.08E-05	1.48E-03
PHB2/SCARNA12	prohibitin 2/SCARNA12	6.25E-04	1.89E-02
PHIP/TRNAF13P (TRF-GAA8-1)	pleckstrin homology domain interacting protein/ transfer RNA-Phe (GAA) 8-1	2.70E-04	1.04E-02
PJA2	praja ring finger 2	4.33E-06	4.27E-04
PLCL2	phospholipase C-like 2	1.94E-03	4.40E-02
PLDN (BLOC1S6)/SQRDL	biogenesis of lysosomal organelles complex-1, subunit 6, pallidin/ sulfide quinone reductase-like (yeast)	2.21E-03	4.84E-02
PLEK	pleckstrin	1.86E-09	5.93E-07
PLEKHB2	pleckstrin homology domain containing, family B (evectins) member 2	3.65E-07	5.45E-05
PLEKHM1P	pleckstrin homology domain containing, family M (with RUN domain) member 1 pseudogene	4.23E-04	1.44E-02
PNRC1	proline-rich nuclear receptor coactivator 1	1.57E-03	3.82E-02
PPIL3/CLK1	peptidylprolyl isomerase (cyclophilin)-like 3/CDC-like kinase 1	5.88E-04	1.81E-02
PPP1CB/SPDYA	protein phosphatase 1, catalytic subunit, beta isozyme/speedy/RINGO cell cycle regulator family member A	4.45E-06	4.29E-04
PPP1CC	protein phosphatase 1, catalytic subunit, gamma isoform	8.17E-05	4.13E-03
PPP1R15B	protein phosphatase 1, regulatory (inhibitor) subunit 15B	1.38E-03	3.50E-02
PPP2R5A	protein phosphatase 2, regulatory subunit B', alpha isoform	1.25E-07	2.24E-05
PPP3CB	protein phosphatase 3 (formerly 2B), catalytic subunit, beta isoform	2.19E-04	8.68E-03
PPP3R1/WDR92	protein phosphatase 3, regulatory subunit B, alpha/WD repeat domain 92	1.50E-06	1.79E-04
PPP4R1	protein phosphatase 4, regulatory subunit 1	5.17E-04	1.67E-02
PPP6C	protein phosphatase 6, catalytic subunit	1.84E-04	7.55E-03
PPTC7	PTC7 protein phosphatase homolog (S. cerevisiae)	4.64E-05	2.67E-03
PRCP	prolylcarboxypeptidase (angiotensinase C)	1.01E-03	2.72E-02
PRMT2	protein arginine methyltransferase 2	1.75E-03	4.14E-02
PRNP	prion protein	5.70E-04	1.77E-02

PRPF38B	PRP38 pre-mRNA processing factor 38 (yeast) domain containing B	7.61E-04	2.21E-02
PSMA1/COPB1	proteasome (prosome, macropain) subunit, alpha type, 1/coatomer protein complex, subunit beta 1	2.16E-03	4.76E-02
PSMB3	proteasome (prosome, macropain) subunit, beta type, 3	2.03E-03	4.56E-02
PSMB8	proteasome (prosome, macropain) subunit, beta type, 8 (large multifunctional peptidase 7)	6.21E-05	3.42E-03
PSMD13	proteasome (prosome, macropain) 26S subunit, non-ATPase, 13	8.66E-07	1.14E-04
PSMD6	proteasome (prosome, macropain) 26S subunit, non-ATPase, 6	3.66E-07	5.45E-05
PTGER4	prostaglandin E receptor 4 (subtype EP4)	6.30E-04	1.90E-02
PTPRC	protein tyrosine phosphatase, receptor type, C	1.77E-23	1.59E-19
PXK	PX domain containing serine/threonine kinase	2.74E-04	1.04E-02
RAB10	RAB10, member RAS oncogene family	8.67E-07	1.14E-04
RAB1A	RAB1A, member RAS oncogene family	1.18E-04	5.47E-03
RAB32	RAB32, member RAS oncogene family	1.48E-03	3.67E-02
RAB6A	RAB6C, member RAS oncogene family; RAB6A, member RAS oncogene family; hypothetical LOC100130819; RAB6C-like	1.20E-04	5.51E-03
RAB8B	RAB8B, member RAS oncogene family	2.86E-04	1.08E-02
RAD21	RAD21 homolog (S. pombe)	2.14E-03	4.74E-02
RAF1	v-raf-1 murine leukemia viral oncogene homolog 1	4.83E-04	1.61E-02
RAP1A	RAP1A, member of RAS oncogene family	4.21E-09	1.21E-06
RAP1B	RAP1B, member of RAS oncogene family	3.01E-12	2.99E-09
RASSF3	Ras association (RalGDS/AF-6) domain family member 3	1.51E-03	3.70E-02
RBBP4	hypothetical LOC642954; retinoblastoma binding protein 4	9.69E-04	2.64E-02
RBL2	retinoblastoma-like 2 (p130)	4.62E-06	4.30E-04
RECQL	RecQ protein-like (DNA helicase Q1-like)	4.93E-05	2.82E-03
RFWD2	ring finger and WD repeat domain 2	9.23E-06	7.50E-04
RGS18	regulator of G-protein signaling 18	8.81E-04	2.43E-02
RICTOR	RPTOR independent companion of MTOR, complex 2	3.07E-08	7.22E-06
RILPL2	Rab interacting lysosomal protein-like 2	4.59E-06	4.30E-04
RIT1	Ras-like without CAAX 1	1.18E-04	5.47E-03
RNF103/VPS24 (CHMP3)	ring finger protein 103/charged multivesicular body protein 3	5.29E-04	1.67E-02
RNF13	ring finger protein 13	1.73E-08	4.30E-06

RNF141	ring finger protein 141	1.20E-05	9.15E-04
RNF145	ring finger protein 145	7.82E-09	2.06E-06
RNF213	ring finger protein 213	1.13E-10	5.60E-08
RNF31/IRF9	ring finger protein 31/interferon regulatory factor 9	2.70E-04	1.04E-02
RNF5	ring finger protein 5; ring finger protein 5 pseudogene 1	1.97E-03	4.45E-02
RNF6	ring finger protein (C3H2C3 type) 6	4.21E-04	1.44E-02
ROCK1	similar to Rho-associated, coiled-coil containing protein kinase 1; Rho-associated, coiled-coil containing protein kinase 1	1.46E-03	3.65E-02
RPL14.1	ribosomal protein L14	1.80E-03	4.19E-02
S100A6	S100 calcium binding protein A6	1.51E-03	3.70E-02
SACM1L	SAC1 suppressor of actin mutations 1-like (yeast)	5.04E-04	1.66E-02
SAR1A/TYSND1/AIFM2	secretion associated, Ras related GTPase 1A/trypsin domain containing 1/apoptosis-inducing factor, mitochondrion-associated, 2	3.26E-07	5.12E-05
SCP2	sterol carrier protein 2	4.97E-05	2.83E-03
SCPEP1	serine carboxypeptidase 1	8.05E-05	4.11E-03
SDHD	succinate dehydrogenase complex, subunit D, integral membrane protein	2.18E-03	4.80E-02
SEC22B	SEC22 vesicle trafficking protein homolog B (S. cerevisiae)	4.13E-04	1.42E-02
SEC61B	Sec61 beta subunit	8.05E-05	4.11E-03
SELE/SELL	selectin E/selectin L	2.96E-04	1.10E-02
SENP6	SUMO1/sentrin specific peptidase 6	1.73E-03	4.10E-02
SEPT5/GP1BB	septin 5/glycoprotein Ib (platelet), beta polypeptide	1.31E-03	3.38E-02
SERINC1	serine incorporator 1	1.36E-07	2.39E-05
SERINC3	serine incorporator 3	1.02E-04	4.88E-03
SKAP2	src kinase associated phosphoprotein 2	1.03E-03	2.76E-02
SKIV2L2	superkiller viralicidic activity 2-like 2 (S. cerevisiae)	1.61E-04	6.94E-03
SLA	Src-like-adaptor	3.32E-04	1.18E-02
SLBP	stem-loop binding protein	1.87E-03	4.29E-02
SLC12A7	solute carrier family 12 (potassium/chloride transporters), member 7	2.26E-03	4.91E-02
SLC25A37	solute carrier family 25, member 37	9.13E-05	4.51E-03
SLK	STE20-like kinase (yeast)	1.49E-04	6.55E-03
SLU7	SLU7 splicing factor homolog (S. cerevisiae)	8.40E-04	2.36E-02
SMAP2	small ArfGAP2	1.92E-03	4.35E-02

SMARCA5	SWI/SNF related, matrix associated, actin dependent regulator of chromatin, subfamily a, member 5	9.51E-04	2.60E-02
SMCHD1	structural maintenance of chromosomes flexible hinge domain containing 1	2.21E-10	9.86E-08
SNX10	sorting nexin 10	1.40E-06	1.71E-04
SNX14	sorting nexin 14	2.09E-06	2.30E-04
SNX2	sorting nexin 2	1.60E-03	3.88E-02
SNX6	sorting nexin 6	3.38E-05	2.16E-03
SP140L/SP100/HMGB1L3	SP140 nuclear body protein-like/SP100 nuclear antigen/high mobility group box 1 pseudogene 3	1.69E-04	7.17E-03
SPATA13/C1QTNF9	spermatogenesis associated 13/C1q and tumor necrosis factor related protein 9	1.80E-06	2.01E-04
SPCS3	signal peptidase complex subunit 3 homolog (S. cerevisiae)	4.54E-06	4.30E-04
SPOPL	speckle-type POZ protein-like	8.45E-10	3.02E-07
SPPL2A	signal peptide peptidase-like 2A	3.22E-05	2.08E-03
SRI	sorcin	3.49E-06	3.54E-04
SRP9/EPHX1	signal recognition particle 9kDa/epoxide hydrolase 1, microsomal (xenobiotic)	7.37E-08	1.50E-05
SSFA2	sperm specific antigen 2	9.73E-04	2.64E-02
ST8SIA4	ST8 alpha-N-acetyl-neuraminide alpha-2,8-sialyltransferase 4	1.14E-03	3.00E-02
STAT1	signal transducer and activator of transcription 1, 91kDa	8.77E-04	2.43E-02
STOM	stomatin	4.35E-05	2.56E-03
STXBP3	syntaxin binding protein 3	1.24E-06	1.56E-04
SURF4	surfeit 4	1.20E-03	3.15E-02
SYTL2	synaptotagmin-like 2	9.74E-04	2.64E-02
TAF1	TAF1 RNA polymerase II, TATA box binding protein (TBP)-associated factor, 250kDa	4.42E-04	1.49E-02
TAGAP	T-cell activation RhoGTPase activating protein	1.01E-04	4.87E-03
TAX1BP1	Tax1 (human T-cell leukemia virus type I) binding protein 1	1.91E-05	1.36E-03
TBC1D2B	TBC1 domain family, member 2B	6.79E-05	3.66E-03
TCF25/MC1R/TUBB3	transcription factor 25 (basic helix-loop-helix)/melanocortin 1 receptor (alpha melanocyte stimulating hormone receptor)/tubulin, beta 3 class III	2.12E-09	6.52E-07
TCP11L2	t-complex 11 (mouse)-like 2	3.73E-04	1.31E-02
TDG	similar to G/T mismatch-specific thymine DNA glycosylase; thymine-DNA glycosylase	1.87E-03	4.29E-02
TDP2	tyrosyl-DNA phosphodiesterase 2	1.70E-03	4.07E-02
TES	testis derived transcript (3 LIM domains)	2.74E-04	1.04E-02

TGFBR2	transforming growth factor, beta receptor II (70/80kDa)	1.05E-04	4.97E-03
TM9SF2	transmembrane 9 superfamily member 2	2.95E-10	1.25E-07
TM9SF3	transmembrane 9 superfamily member 3	1.74E-04	7.26E-03
TMCC1	transmembrane and coiled-coil domain family 1	2.13E-04	8.52E-03
TMCO3	transmembrane and coiled-coil domains 3	7.11E-05	3.78E-03
TMEM167B	transmembrane protein 167B	2.78E-05	1.85E-03
TMEM222	transmembrane protein 222	1.41E-04	6.27E-03
TMEM49	transmembrane protein 49	5.38E-06	4.86E-04
TMEM59	transmembrane protein 59	8.49E-05	4.26E-03
TMSB4X	thymosin-like 2 (pseudogene); thymosin-like 1 (pseudogene); thymosin beta 4, X-linked	6.06E-04	1.85E-02
TNFSF13B	tumor necrosis factor (ligand) superfamily, member 13b	3.47E-04	1.23E-02
TNKS2	tankyrase, TRF1-interacting ankyrin-related ADP-ribose polymerase 2	8.73E-04	2.42E-02
TNPO3	transportin 3	4.02E-05	2.41E-03
TOPORS/DDX58	topoisomerase I binding, arginine/serine-rich, E3 ubiquitin protein ligase/DEAD (Asp-Glu-Ala-Asp) box polypeptide 58	6.89E-04	2.03E-02
TOR1A	torsin family 1, member A (torsin A)	1.11E-07	2.02E-05
TOR1AIP1	torsin A interacting protein 1	5.49E-04	1.73E-02
TPM3	tropomyosin 3	4.32E-07	6.23E-05
TRAM1	translocation associated membrane protein 1	1.79E-06	2.01E-04
TRPC4AP	transient receptor potential cation channel, subfamily C, member 4 associated protein	1.30E-05	9.81E-04
TSNAX/DISC1	translin-associated factor X/disrupted in schizophrenia 1	4.89E-04	1.62E-02
TSPAN14	tetraspanin 14	1.16E-05	8.98E-04
TXNRD1	thioredoxin reductase 1; hypothetical LOC100130902	8.52E-08	1.65E-05
U2AF1	U2 small nuclear RNA auxiliary factor 1	3.82E-05	2.33E-03
UBE2B	ubiquitin-conjugating enzyme E2B (RAD6 homolog)	2.65E-04	1.02E-02
UBE2E3	ubiquitin-conjugating enzyme E2E 3 (UBC4/5 homolog, yeast)	6.50E-04	1.95E-02
UBL7	ubiquitin-like 7 (bone marrow stromal cell-derived)	2.09E-03	4.66E-02
UBR2	ubiquitin protein ligase E3 component n-recognin 2	7.78E-04	2.21E-02
UHMK1	U2AF homology motif (UHM) kinase 1	1.28E-03	3.32E-02
USP1	ubiquitin specific peptidase 1	1.36E-03	3.47E-02
USP15	ubiquitin specific peptidase 15	2.60E-05	1.75E-03
USP33	ubiquitin specific peptidase 33	7.17E-04	2.09E-02

UTRN	utrophin	5.40E-15	9.66E-12
VAMP3	vesicle-associated membrane protein 3 (cellubrevin)	1.62E-04	6.94E-03
VCP	valosin-containing protein	6.72E-04	2.00E-02
VNN2	vanin 2	1.49E-03	3.69E-02
VPS13C	vacuolar protein sorting 13 homolog C (S. cerevisiae)	3.31E-04	1.18E-02
WARS	tryptophanyl-tRNA synthetase	1.44E-04	6.37E-03
WDFY2	WD repeat and FYVE domain containing 2	1.87E-03	4.29E-02
WSB1	WD repeat and SOCS box-containing 1	1.39E-04	6.20E-03
yakeme		5.07E-04	1.66E-02
YIPF4	Yip1 domain family, member 4	2.65E-04	1.02E-02
YWHAE	similar to 14-3-3 protein epsilon (14-3-3E) (Mitochondrial import stimulation factor L subunit) (MSF L); tyrosine 3-monooxygenase/tryptophan 5-monooxygenase activation protein, epsilon polypeptide	9.45E-10	3.13E-07
ZBED5/EIF4G2/SNORD97	zinc finger, BED-type containing 5/eukaryotic translation initiation factor 4 gamma, 2/small nucleolar RNA, C/D box 97	4.27E-04	1.45E-02
ZCCHC6	zinc finger, CCHC domain containing 6	3.66E-05	2.26E-03
ZEB2/GTDC1	zinc finger E-box binding homeobox 2/glycosyltransferase-like domain containing 1	1.36E-04	6.12E-03
ZFAND5	zinc finger, AN1-type domain 5	2.10E-05	1.48E-03
ZFP91-CNTF	zinc finger protein 91 homolog (mouse); ZFP91-CNTF readthrough transcript; ciliary neurotrophic factor	9.98E-06	8.03E-04
ZNF516	zinc finger protein 516	2.98E-04	1.10E-02
ZNF592	zinc finger protein 592	1.35E-03	3.45E-02
