# Supplementary material for: Intracerebral Hemorrhage and Ischemic Stroke of Different Etiologies Have Distinct Alternatively Spliced mRNA Profiles in the Blood: a Pilot RNA-seq Study
Source: Transl Stroke Res. 2015 May 22;6(4):284–9. doi: 10.1007/s12975-015-0407-9 (PMC4485700; doi:10.1007/s12975-015-0407-9)
Supplement: Supplementary file 8 — Canonical pathways of the 412 genes with DAS among Large Vessel Ischemic Stroke (IS), Cardioembolic IS, Lacunar IS, Intracerebral Hemorrhage (ICH) and Control subjects. Benjamini-Hochberg corrected P values for multiple comparison corrections. (PDF 51 kb) [file 12975_2015_407_MOESM4_ESM.pdf]

SUPPLEMENTARY TABLE 4. Canonical Pathways of the 412 Genes with Differential Alternative Splicing Among Large Vessel Ischemic Stroke (IS), Cardioembolic IS, Lacunar IS, Intracerebral Hemorrhage and Control Subjects. B-H-Benjamini-Hochberg corrected p-value				
**Ingenuity Canonical Pathways**	**-log(p-value)**	**-log(B-H p-value)**	**Ratio**	**Molecules**
CD28 Signaling in T Helper Cells	8.11E00	5.54E00	1.36E-01	CDC42,ACTR2,CALM1 (includes others),ARPC3,HLA-DQA1,ATM,PPP3CB,ARPC5L,ACTR3,HLA-DRB1,PPP3R1,HLA-DMA,ARPC4,HLA-DMB,PTPRC,CD86
Cdc42 Signaling	5.99E00	3.72E00	9.58E-02	CDC42,ACTR2,ARPC3,HLA-DQA1,RAF1,CDC42SE1,MYL12A,ARPC5L,ACTR3,HLA-DRB1,HLA-DMA,ARPC4,HLA-DMB,PPP1CB,ITGA4,IQGAP2
Nur77 Signaling in T Lymphocytes	5.38E00	3.33E00	1.58E-01	HLA-DRB1,CALM1 (includes others),PPP3R1,APAF1,HLA-DMA,HLA-DQA1,HLA-DMB,PPP3CB,CD86
fMLP Signaling in Neutrophils	5.3E00	3.33E00	1.11E-01	CDC42,ACTR2,CALM1 (includes others),PPP3R1,ARPC3,RAF1,ARPC4,ATM,PPP3CB,ARPC5L,CYBB,ACTR3
Interferon Signaling	4.92E00	3.04E00	1.94E-01	IFNGR1,IRF9,JAK2,STAT1,MX1,IFNAR1,PSMB8
Rac Signaling	4.7E00	2.91E00	1.06E-01	CDC42,ACTR2,ARPC3,RAF1,ARPC4,ATM,ITGA4,IQGAP2,ARPC5L,CYBB,ACTR3
Actin Nucleation by ARP-WASP Complex	4.51E00	2.83E00	1.43E-01	CDC42,ACTR2,ARPC3,ARPC4,ROCK1,ITGA4,ARPC5L,ACTR3
Regulation of Actin-based Motility by Rho	4.47E00	2.83E00	1.1E-01	CDC42,ACTR2,ARPC3,ARPC4,MYL12A,PPP1CB,ROCK1,ITGA4,ARPC5L,ACTR3
Ephrin Receptor Signaling	4.45E00	2.83E00	8.05E-02	CDC42,ACTR2,JAK2,ARPC3,EPHB4,CRKL,RAF1,ARPC5L,RAP1A,ACTR3,ARPC4,RAP1B,ROCK1,ITGA4
Integrin Signaling	4.32E00	2.75E00	7.43E-02	CDC42,ACTR2,ARPC3,CRKL,RAF1,ATM,MYL12A,ARPC5L,RAP1A,ACTR3,ARPC4,RAP1B,PPP1CB,ROCK1,ITGA4
Protein Kinase A Signaling	4.28E00	2.75E00	5.73E-02	ANAPC13,PPP1CC,TDP2,CALM1 (includes others),MPPE1,ADCY7,RAF1,AKAP8,MYL12A,H3F3A/H3F3B,PPP3CB,YWHAE,RAP1A,ADD3,TGFBR2,PPP3R1,RAP1B,PPP1CB,ROCK1,PLCL2,PTPRC,ANAPC7
B Cell Development	4.04E00	2.54E00	1.76E-01	HLA-DRB1,HLA-DMA,HLA-DQA1,HLA-DMB,PTPRC,CD86
Actin Cytoskeleton Signaling	3.97E00	2.51E00	6.91E-02	CDC42,ACTR2,ARPC3,CRKL,RAF1,ATM,MYL12A,ARPC5L,ACTR3,ARPC4,PPP1CB,ROCK1,ITGA4,IQGAP2,TMSB10/TMSB4X

Ingenuity Canonical Pathways	-log(p-value)	-log(B-H p-value)	Ratio	Molecules
Antigen Presentation Pathway	3.83E00	2.4E00	1.62E-01	HLA-DRB1,CD74,HLA-DMA,HLA-DQA1,HLA-DMB,PSMB8
Role of JAK1, JAK2 and TYK2 in Interferon Signaling	3.8E00	2.4E00	2.08E-01	IFNGR1,JAK2,STAT1,RAF1,IFNAR1
T Helper Cell Differentiation	3.76E00	2.39E00	1.13E-01	IFNGR1,TGFBR2,HLA-DRB1,HLA-DMA,HLA-DQA1,STAT1,HLA-DMB,CD86
Calcium-induced T Lymphocyte Apoptosis	3.28E00	1.96E00	1.09E-01	HLA-DRB1,CALM1 (includes others),PPP3R1,HLA-DMA,HLA-DQA1,HLA-DMB,PPP3CB
Retinoic acid Mediated Apoptosis Signaling	3.28E00	1.96E00	1.09E-01	CFLAR,APAF1,TNKS2,PARP9,PARP14,DAP3,IFNAR1
iCOS-iCOSL Signaling in T Helper Cells	3.17E00	1.9E00	8.33E-02	HLA-DRB1,CALM1 (includes others),PPP3R1,HLA-DMA,HLA-DQA1,ATM,HLA-DMB,PPP3CB,PTPRC
Dendritic Cell Maturation	3.17E00	1.9E00	6.7E-02	HLA-DRB1,JAK2,HLA-DMA,HLA-DQA1,CD58,STAT1,ATM,HLA-DMB,PLCL2,LY75,CD86,IFNAR1
NRF2-mediated Oxidative Stress Response	3.15E00	1.9E00	6.67E-02	GSTO1,BACH1,RAF1,ATM,UBE2E3,TXNRD1,EPHX1,DNAJB6,VCP,GSTK1,MGST1,NFE2L2
Type I Diabetes Mellitus Signaling	3.11E00	1.88E00	8.18E-02	IFNGR1,HLA-DRB1,APAF1,JAK2,HLA-DMA,HLA-DQA1,STAT1,HLA-DMB,CD86
IL-3 Signaling	3.01E00	1.81E00	9.86E-02	PPP3R1,JAK2,CRKL,STAT1,RAF1,ATM,PPP3CB
ERK/MAPK Signaling	3E00	1.81E00	6.42E-02	PPP1CC,PPP2R5A,CRKL,STAT1,RAF1,ATM,RAP1B,H3F3A/H3F3B,PPP1CB,ITGA4,RAP1A,PPP2CA
Breast Cancer Regulation by Stathmin1	2.92E00	1.75E00	6.28E-02	PPP1CC,CDC42,CALM1 (includes others),UHMK1,PPP2R5A,TUBB3,ADCY7,RAF1,ATM,PPP1CB,ROCK1,PPP2CA
Synaptic Long Term Potentiation	2.87E00	1.71E00	7.56E-02	PPP1CC,CALM1 (includes others),PPP3R1,RAF1,RAP1B,PPP3CB,PPP1CB,PLCL2,RAP1A
Ascorbate Recycling (Cytosolic)	2.84E00	1.71E00	6.67E-01	GSTO1,GLRX
IL-4 Signaling	2.83E00	1.71E00	9.21E-02	HLA-DRB1,JAK2,HLA-DMA,HLA-DQA1,NR3C1,ATM,HLA-DMB
Role of NFAT in Regulation of the Immune Response	2.81E00	1.71E00	6.43E-02	HLA-DRB1,CALM1 (includes others),PPP3R1,HLA-DMA,HLA-DQA1,RAF1,ATM,HLA-DMB,PPP3CB,FCER1A,CD86
Epithelial Adherens Junction Signaling	2.8E00	1.71E00	6.85E-02	TGFBR2,CDC42,ACTR2,ARPC3,TUBB3,ARPC4,RAP1B,ARPC5L,RAP1A,ACTR3

Ingenuity Canonical Pathways	-log(p-value)	-log(B-H p-value)	Ratio	Molecules
RhoA Signaling	2.79E00	1.71E00	7.38E-02	ACTR2,ARPC3,ARPC4,MYL12A,PPP1CB,ROCK1,ARPC5L,ACTR3,SEPT5
Signaling by Rho Family GTPases	2.64E00	1.57E00	5.56E-02	CDC42,ACTR2,ARPC3,RAF1,ATM,MYL12A,ARPC5L,ACTR3,SEPT5,ARPC4,ROCK1,ITGA4,CYBB
Production of Nitric Oxide and Reactive Oxygen Species in Macrophages	2.63E00	1.57E00	6.11E-02	PPP1CC,IFNGR1,JAK2,PPP2R5A,STAT1,ATM,RAP1B,PPP1CB,CYBB,RAP1A,PPP2CA
GM-CSF Signaling	2.61E00	1.56E00	9.68E-02	PPP3R1,JAK2,STAT1,RAF1,ATM,PPP3CB
Leukotriene Biosynthesis	2.48E00	1.47E00	2.14E-01	DPEP3,DPEP2,LTA4H
Vitamin-C Transport	2.48E00	1.47E00	2.14E-01	GSTO1,GLRX,TXNRD1
Mitotic Roles of Polo-Like Kinase	2.47E00	1.47E00	9.09E-02	SLK,ANAPC13,RAD21,PPP2R5A,ANAPC7,PPP2CA
CTLA4 Signaling in Cytotoxic T Lymphocytes	2.47E00	1.47E00	7.95E-02	JAK2,PPP2R5A,CLTC,ATM,AP1S2,CD86,PPP2CA
cAMP-mediated signaling	2.43E00	1.45E00	5.48E-02	RGS18,TDP2,CALM1 (includes others),PPP3R1,ADCY7,MPPE1,RAF1,AKAP8,PPP3CB,PTGER4,RAP1A,MC1R
Axonal Guidance Signaling	2.41E00	1.44E00	4.39E-02	CDC42,ACTR2,ARPC3,EPHB4,TUBB3,CRKL,RAF1,ATM,MYL12A,PPP3CB,ARPC5L,RAP1A,ACTR3,PPP3R1,ARPC4,RAP1B,ROCK1,ITGA4,PLCL2
Remodeling of Epithelial Adherens Junctions	2.4E00	1.44E00	8.82E-02	ACTR2,ARPC3,TUBB3,ARPC4,ARPC5L,ACTR3
Graft-versus-Host Disease Signaling	2.39E00	1.43E00	1.04E-01	HLA-DRB1,HLA-DMA,HLA-DQA1,HLA-DMB,CD86
Autoimmune Thyroid Disease Signaling	2.35E00	1.41E00	1.02E-01	HLA-DRB1,HLA-DMA,HLA-DQA1,HLA-DMB,CD86
Fcγ Receptor-mediated Phagocytosis in Macrophages and Monocytes	2.33E00	1.4E00	7.53E-02	CDC42,ACTR2,VAMP3,ARPC3,ARPC4,ARPC5L,ACTR3
Protein Ubiquitination Pathway	2.32E00	1.4E00	5.1E-02	UBR2,UBE2E3,PSMD6,USP1,USP15,UBE2B,PSMD13,DNAJB6,PSMB3,PAN2,PSMA1,USP33,PSMB8
PKCθ Signaling in T Lymphocytes	2.32E00	1.4E00	6.78E-02	HLA-DRB1,PPP3R1,HLA-DMA,HLA-DQA1,ATM,HLA-DMB,PPP3CB,CD86
Glucocorticoid Receptor Signaling	2.24E00	1.34E00	4.98E-02	JAK2,RAF1,ATM,PPP3CB,SELE,ANXA1,TGFBR2,HMGB1,PPP3R1,NR3C1,STAT1,CDKN1C,TAF1

Ingenuity Canonical Pathways	-log(p-value)	-log(B-H p-value)	Ratio	Molecules
Glutathione Redox Reactions I	2.16E00	1.27E00	1.67E-01	GPX1,GSTK1,MGST1
PDGF Signaling	2.14E00	1.27E00	7.79E-02	EIF2AK2,JAK2,CRKL,STAT1,RAF1,ATM
Role of Pattern Recognition Receptors in Recognition of Bacteria and Viruses	2.13E00	1.27E00	6.3E-02	EIF2AK2,EIF2S1,IFIH1,OAS3,ATM,OAS2,DDX58,CLEC7A
D-myo-inositol (1,4,5,6)-Tetrakisphosphate Biosynthesis	2.13E00	1.27E00	6.3E-02	PPP1CC,PPTC7,PPP2R5A,PPP4R1,SACM1L,IPMK,PTPRC,MTMR6
D-myo-inositol (3,4,5,6)-tetrakisphosphate Biosynthesis	2.13E00	1.27E00	6.3E-02	PPP1CC,PPTC7,PPP2R5A,PPP4R1,SACM1L,IPMK,PTPRC,MTMR6
Clathrin-mediated Endocytosis Signaling	2.07E00	1.22E00	5.41E-02	CDC42,ACTR2,PPP3R1,ARPC3,CLTC,ARPC4,ATM,PPP3CB,ARPC5L,ACTR3
HGF Signaling	2.05E00	1.21E00	6.67E-02	CDC42,CRKL,RAF1,ATM,RAP1B,ITGA4,RAP1A
Cardiac β-adrenergic Signaling	2.01E00	1.18E00	6.02E-02	PPP1CC,TDP2,PPP2R5A,ADCY7,MPPE1,AKAP8,PPP1CB,PPP2CA
Dopamine-DARPP32 Feedback in cAMP Signaling	2E00	1.18E00	5.59E-02	PPP1CC,CALM1 (includes others),PPP3R1,PPP2R5A,ADCY7,PPP3CB,PPP1CB,PLCL2,PPP2CA
Cardiac Hypertrophy Signaling	1.95E00	1.13E00	4.93E-02	TGFBR2,CALM1 (includes others),PPP3R1,ADCY7,RAF1,ATM,MYL12A,PPP3CB,ROCK1,PLCL2,ADSS
Role of PKR in Interferon Induction and Antiviral Response	1.93E00	1.12E00	1E-01	EIF2AK2,EIF2S1,APAF1,STAT1
HIPPO signaling	1.91E00	1.11E00	6.98E-02	PPP1CC,PPP2R5A,MOB1A,PPP1CB,YWHAE,PPP2CA
Salvage Pathways of Pyrimidine Deoxyribonucleotides	1.9E00	1.1E00	2.5E-01	APOBEC3B,APOBEC3A
Altered T Cell and B Cell Signaling in Rheumatoid Arthritis	1.87E00	1.09E00	6.82E-02	TNFSF13B,HLA-DRB1,HLA-DMA,HLA-DQA1,HLA-DMB,CD86
UVA-Induced MAPK Signaling	1.87E00	1.09E00	6.82E-02	STAT1,ATM,TNKS2,PARP9,PLCL2,PARP14
Activation of IRF by Cytosolic Pattern Recognition Receptors	1.86E00	1.09E00	7.81E-02	IRF9,STAT1,IFIH1,DDX58,IFNAR1

Ingenuity Canonical Pathways	-log(p-value)	-log(B-H p-value)	Ratio	Molecules
3-phosphoinositide Degradation	1.82E00	1.05E00	5.56E-02	PPP1CC,MTMR1,PPTC7,PPP2R5A,PPP4R1,SACM1L,PTPRC,MTMR6
RhoGDI Signaling	1.81E00	1.05E00	5.2E-02	CDC42,ACTR2,ARPC3,ARPC4,MYL12A,ROCK1,ITGA4,ARPC5L,ACTR3
iNOS Signaling	1.79E00	1.04E00	9.09E-02	IFNGR1,CALM1 (includes others),JAK2,STAT1
Death Receptor Signaling	1.78E00	1.03E00	6.52E-02	CFLAR,APAF1,TNKS2,ROCK1,PARP9,PARP14
B Cell Receptor Signaling	1.77E00	1.03E00	5.11E-02	CDC42,CALM1 (includes others),PPP3R1,RAF1,ATM,RAP1B,PPP3CB,PTPRC,RAP1A
PI3K/AKT Signaling	1.7E00	9.65E-01	5.69E-02	JAK2,PPP2R5A,MCL1,RAF1,ITGA4,YWHAE,PPP2CA
T Cell Receptor Signaling	1.68E00	9.48E-01	6.19E-02	CALM1 (includes others),PPP3R1,RAF1,ATM,PPP3CB,PTPRC
Histamine Biosynthesis	1.65E00	9.38E-01	1E00	HDC
UDP-N-acetyl-D-galactosamine Biosynthesis I	1.65E00	9.38E-01	1E00	GALE
3-phosphoinositide Biosynthesis	1.65E00	9.38E-01	5.16E-02	PPP1CC,PPTC7,PPP2R5A,PPP4R1,ATM,SACM1L,PTPRC,MTMR6
CDK5 Signaling	1.64E00	9.38E-01	6.06E-02	PPP1CC,PPP2R5A,ADCY7,RAF1,PPP1CB,PPP2CA
Prolactin Signaling	1.64E00	9.38E-01	6.85E-02	JAK2,STAT1,NR3C1,RAF1,ATM
Glutathione-mediated Detoxification	1.63E00	9.34E-01	1.07E-01	GSTO1,GSTK1,MGST1
Superpathway of Inositol Phosphate Compounds	1.55E00	8.69E-01	4.69E-02	PPP1CC,PPTC7,PPP2R5A,PPP4R1,ATM,SACM1L,IPMK,PTPRC,MTMR6
CNTF Signaling	1.55E00	8.69E-01	7.69E-02	JAK2,STAT1,RAF1,ATM
Dopamine Receptor Signaling	1.53E00	8.51E-01	6.41E-02	PPP1CC,PPP2R5A,ADCY7,PPP1CB,PPP2CA
Pancreatic Adenocarcinoma Signaling	1.51E00	8.41E-01	5.66E-02	TGFBR2,CDC42,JAK2,STAT1,RAF1,ATM
Regulation of IL-2 Expression in Activated and Anergic T Lymphocytes	1.51E00	8.41E-01	6.33E-02	TGFBR2,CALM1 (includes others),PPP3R1,RAF1,PPP3CB
NGF Signaling	1.49E00	8.34E-01	5.61E-02	CDC42,RAF1,ATM,RAP1B,ROCK1,RAP1A
Thrombopoietin Signaling	1.47E00	8.18E-01	7.27E-02	JAK2,STAT1,RAF1,ATM
Aryl Hydrocarbon Receptor Signaling	1.43E00	7.84E-01	5E-02	GSTO1,APAF1,RBL2,ATM,GSTK1,MGST1,NFE2L2
Oncostatin M Signaling	1.41E00	7.68E-01	8.82E-02	JAK2,STAT1,RAF1
Inhibition of Angiogenesis by TSP1	1.41E00	7.68E-01	8.82E-02	CD47,TGFBR2,CD36
D-myo-inositol-5-phosphate Metabolism	1.39E00	7.6E-01	4.9E-02	PPP1CC,PPTC7,PPP2R5A,PPP4R1,SACM1L,PTPRC,MTMR6

Ingenuity Canonical Pathways	-log(p-value)	-log(B-H p-value)	Ratio	Molecules
Phospholipase C Signaling	1.38E00	7.6E-01	4.18E-02	CALM1 (includes others),PPP3R1,ADCY7,RAF1,MYL12A,RAP1B,PPP3CB,PPP1CB,ITGA4,RAP1A
Cell Cycle Regulation by BTG Family Proteins	1.38E00	7.6E-01	8.57E-02	PPP2R5A,CNOT7,PPP2CA
Allograft Rejection Signaling	1.37E00	7.6E-01	5.81E-02	HLA-DRB1,HLA-DMA,HLA-DQA1,HLA-DMB,CD86
Telomere Extension by Telomerase	1.37E00	7.6E-01	1.33E-01	TNKS2,HNRNPA2B1
Spliceosomal Cycle	1.36E00	7.6E-01	5E-01	LOC102724594/U2AF1
S-methyl-5-thio-α-D-ribose 1-phosphate Degradation	1.36E00	7.6E-01	5E-01	APIP
Glutamate Biosynthesis II	1.36E00	7.6E-01	5E-01	GLUD1
Glutamate Degradation X	1.36E00	7.6E-01	5E-01	GLUD1
Regulation of eIF4 and p70S6K Signaling	1.35E00	7.59E-01	4.79E-02	EIF2S1,PPP2R5A,RAF1,ATM,ITGA4,EIF4G2,PPP2CA
Calcium Signaling	1.34E00	7.57E-01	4.49E-02	TPM3,ATP2B4,CALM1 (includes others),PPP3R1,RAP1B,PPP3CB,MICU1,RAP1A
Gαq Signaling	1.34E00	7.57E-01	4.76E-02	RGS18,CALM1 (includes others),PPP3R1,RAF1,ATM,PPP3CB,ROCK1
RANK Signaling in Osteoclasts	1.34E00	7.57E-01	5.68E-02	CALM1 (includes others),PPP3R1,RAF1,ATM,PPP3CB
Role of NFAT in Cardiac Hypertrophy	1.33E00	7.56E-01	4.47E-02	TGFBR2,CALM1 (includes others),PPP3R1,ADCY7,RAF1,ATM,PPP3CB,PLCL2
PAK Signaling	1.32E00	7.48E-01	5.62E-02	CDC42,RAF1,ATM,MYL12A,ITGA4
p70S6K Signaling	1.31E00	7.39E-01	5.04E-02	PPP2R5A,RAF1,ATM,PLCL2,YWHAE,PPP2CA
