# Supplementary material for: Intracerebral Hemorrhage and Ischemic Stroke of Different Etiologies Have Distinct Alternatively Spliced mRNA Profiles in the Blood: a Pilot RNA-seq Study
Source: Transl Stroke Res. 2015 May 22;6(4):284–9. doi: 10.1007/s12975-015-0407-9 (PMC4485700; doi:10.1007/s12975-015-0407-9)
Supplement: Supplementary file 9 — Functions associated with the top 2 molecular and cellular functions over-represented in the 412 differentially alternatively spliced genes. (PDF 113 kb) [file 12975_2015_407_MOESM5_ESM.pdf]

SUPPLEMENTARY TABLE 5. Functions Associated with the Top 2 Molecular and Cellular Functions Over-Represented in the 412 Differentially Alternatively Spliced Genes				
Categories	Diseases or Functions Annotation	p-Value	Molecules	# Molecules
Cell Death and Survival	cell death	1.32E-09	ACSL4,AIFM2,AKAP8,ANXA1,ANXA7,APAF1,APH1A,APIP,APOBEC3B,ARL6IP5,ARNTL,ATM,ATP2B4,ATP6V1G2,BAZ1A,BRAT1,C1QTNF9,CALM1 (includes others),CARD8,CCAR1,CCT8,CD164,CD244,CD36,CD46,CD47,CD53,CD74,CD86,CDC42,CDKN1C,CFLAR,CHMP3,CNPY2,CTSS,CYBB,CYLD,DAP3,DDX19A,DDX3X,DDX58,DNAJB6,DPYD,DUSP22,EGLN1,EIF2AK2,EIF2S1,EIF4G2,EPHB4,EPHX1,ERN1,FCER1A,FKBP1A,FNTA,GLRX,GLUD1,GMCL1,GPX1,GZMA,HEXB,HIPK3,HLA-DMA,HLA-DRB1,HMGB1,IFIH1,IFNAR1,IFNGR1,IFRD1,IGFBP7,ING4,IPMK,IQGAP2,ITGA4,JAK2,KLF13,LMO4,LRRK2,MC1R,MCL1,MICA,MOB1A, MTCH1,MTMR6,MTPN,MX1, NCOA4,NFE2L2, NR3C1,OAS3, PAFAH1B1,PARP14,PHB2,PHIP,PPP1CC,PPP1R15B,PPP2CA, PPP2R5A, PPP3CB, PPP3R1, PRMT2,PRNP, PSMB8, PSMD6,PTGER4, PTPRC,RAB1A,RAB32,RAD21,RAF1,RAP1A,RAP1B,RASSF3,RBBP4,RBL2,RECQL,RFWD2,RICTOR, RIT1,RNF13,RNF31, RNF5,ROCK1, S100A6,SCP2,SELL, SERINC3,SLC12A7,SLK,SMARCA5,SRI,ST8SIA4,STAT1,TAX1BP1,TDP2,TGFBR2,TMSB10/TMSB4X,TNFSF13B,TNKS2,TOPORS,TPM3,TUBB3,TXNRD1,UBE2B,VAMP3,VCP,YWHAE,ZEB2,ZFAND5	148
Cell Death and Survival	apoptosis	4.11E-09	ACSL4,AIFM2,AKAP8,ANXA1,ANXA7,APAF1,APH1A,APIP,APOBEC3B,ARL6IP5,ATM,ATP2B4,ATP6V1G2,BAZ1A,BRAT1,C1QTNF9,CARD8,CCAR1,CD164,CD36,CD47,CD53,CD74,CDC42,CDKN1C,CFLAR,CTSS,CYBB,CYLD,DAP3,DDX19A,DDX3X,DDX58,DUSP22,EIF2AK2,EIF2S1,EIF4G2,EPHB4,EPHX1,ERN1,FCER1A,FKBP1A,FNTA,GLRX,GLUD1,GMCL1,GPX1,GZMA,HEXB,HIPK3,HLA-DMA,HMGB1,IFIH1,IFNAR1,IFNGR1,IGFBP7, ING4,IQGAP2,ITGA4,JAK2,KLF13,LMO4,LRRK2,MC1R,MCL1,MOB1A,MTCH1,MTMR6,MTPN,MX1,NCOA4,NFE2L2,NR3C1,OAS3,PAFAH1B1,PARP14,PHB2,PHIP,PPP1CC,PPP2CA,PPP3CB,PPP3R1,PRMT2,PRNP,PSMB8,PSMD6,PTGER4,PTPRC,RAB32,RAD21,RAF1,RAP1A,RAP1B,RASSF3,RBBP4,RBL2,RFWD2,RICTOR,RIT1,RNF13,RNF31,RNF5,ROCK1,S100A6,SELL,SERINC3,SLK,SRI,ST8SIA4,STAT1,TAX1BP1,TDP2,TGFBR2,TMSB10/TMSB4X,TNFSF13B,TOPORS,TXNRD1,UBE2B,VCP,YWHAE,ZEB2,ZFAND5	122
Cell Death and Survival	necrosis	6.80E-05	ANXA1,APAF1,APIP,APOBEC3B,ATM,ATP2B4,ATP6V1G2,BAZ1A,BRAT1,C1QTNF9,CARD8,CCAR1,CCT8,CD36,CD47,CD74,CD86,CDC42,CDKN1C,CFLAR,CNPY2,CTSS,CYBB,CYLD,DAP3,DDX3X,DDX58,DPYD,EGLN1,EIF2AK2,EIF2S1,EIF4G2,EPHB4,EPHX1,ERN1,FCER1A,FKBP1A,FNTA,GLRX,GLUD1,GPX1,GZMA,HLA-DMA,HMGB1,IFIH1,IFNAR1,IFNGR1,IFRD1,IGFBP7,IPMK,IQGAP2,ITGA4,JAK2,KLF13,LMO4,LRRK2,MC1R,MCL1,MTMR6,MTPN,MX1,NFE2L2,NR3C1,OAS3,PAFAH1B1,PARP14,PHIP,PPP1CC,PPP2CA,PPP3CB,PPP3R1,PRMT2,PRNP,PSMB8,PSMD6,PTGER4,PTPRC,RAB32,RAD21,RAF1,RASSF3,RBBP4,RBL2,RECQL,RFWD2,RICTOR,RIT1,RNF13,RNF31,RNF5,ROCK1,S100A6,SCP2,SELL,SLK,SRI,STAT1,TAX1BP1,TDP2,TGFBR2,TMSB10/TMSB4X,TNFSF13B,TUBB3,TXNRD1,VCP,YWHAE	106
Cell Death and Survival	cell viability	2.26E-04	APAF1,APOBEC3A,ATM,C1QTNF9,CD47,CD74,CD86,CDC42,CDKL3,CDKN1C,CFLAR,CYBB,CYLD,DPYD,DUSP22,EPHB4,ERN1,FCER1A,GLUD1,GPX1,HMGB1,IFIH1,IGFBP7,IRF9,JAK2,LRRK2,MCL1,MGST1,MTMR1,MTMR6,MX1,NFE2L2,NR3C1,PARP14,PPP1CB,PPP1CC,PPP2CA,PPP2R5A,PPP4R1,PPP6C,PRNP,PSMA1,PTPRC,RAD21,RAF1,RBBP4,RICTOR,RIT1,RNF31,S100A6,SELE,SELL,SPDYA,STAT1,TDP2,TGFBR2,TNFSF13B,TUBB3,VCP	59
Cell Death and Survival, DNA Replication, Recombination, and Repair	fragmentation of DNA	3.36E-04	APAF1,APOBEC3B,CD53,EIF2AK2,GPX1,GZMA,MCL1,MTCH1,NR3C1,PPP1CC,PRNP,SERINC3,STAT1	13
Cell Death and Survival	cell death of tumor cell lines	3.43E-04	APAF1,ATM,BAZ1A,BRAT1,CARD8,CCAR1,CCT8,CD47,CDC42,CDKN1C,CFLAR,CNPY2,CYLD,DAP3,DDX3X,DDX58,DPYD,EGLN1,EIF2AK2,EIF2S1,EIF4G2,EPHB4,ERN1,FCER1A,FKBP1A,GLRX,GPX1,IFNAR1,IFRD1,IGFBP7,IPMK,IQGAP2,ITGA4,JAK2,LMO4,MCL1,MTMR6,NFE2L2,NR3C1,OAS3,PARP14,PHIP,PPP2CA,PRNP,PTPRC,RAB32,RAD21,RAF1,RASSF3,RBL2,RFWD2,RICTOR,RIT1,RNF13,RNF31,RNF5,S100A6,SRI,STAT1,TDP2,TGFBR2,TMSB10/TMSB4X,TNFSF13B,TUBB3,TXNRD1,VCP,YWHAE	67
Cell Death and Survival	cell survival	3.95E-04	APAF1,APOBEC3A,ATM,C1QTNF9,CD47,CD74,CD86,CDC42,CDKL3,CDKN1C,CFLAR,CYBB,CYLD,DDX3X,DPYD,DUSP22,EIF2S1,EPHB4,ERN1,FCER1A,GLRX,GLUD1,GPX1,HMGB1,IFIH1,IGFBP7,IRF9,JAK2,LRRK2,MCL1,MGST1,MTMR1,MTMR6,MX1,NFE2L2,NR3C1,PARP14,PPP1CB,PPP1CC,PPP2CA,PPP2R5A,PPP4R1,PPP6C,PRNP,PSMA1,PTPRC,RAD21,RAF1,RBBP4,RICTOR,RIT1,RNF31,S100A6,SELE,SELL,SPDYA,STAT1,TDP2,TGFBR2,TNFSF13B,TUBB3,VCP	62
Cell Death and Survival	cytolysis	4.21E-04	ANXA1,CD244,CD46,CD47,FCER1A,GZMA,IFNAR1,MICA,NFE2L2,NR3C1,PPP3CB,PPP3R1,PTPRC,TGFBR2	14
Cardiovascular System Development and Function, Cell Death and Survival, Cellular Development, Organ Morphology, Tissue Development, Tissue Morphology	regeneration of cardiomyocytes	4.91E-04	HMGB1,MTPN	2
Cell Death and Survival, Connective Tissue Disorders, Hematological Disease	hemolysis	5.26E-04	ANXA1,CD47,IFNAR1,NFE2L2,NR3C1,PPP3CB,PPP3R1	7
Cell Death and Survival	cell death of immune cells	5.41E-04	ANXA1,APAF1,ATM,CD47,CD74,CD86,CDC42,CFLAR,CYBB,CYLD,EIF2AK2,ERN1,FCER1A,GZMA,HMGB1,IFIH1,IFNAR1,IFNGR1,ITGA4,JAK2,KLF13,MCL1,MX1,NFE2L2,NR3C1,PARP14,PPP3CB,PTGER4,PTPRC,RAF1,STAT1,TGFBR2,TNFSF13B	33

Categories	Diseases or Functions Annotation	p-Value	Molecules	# Molecules
Cancer, Cell Death and Survival, Tumor Morphology	cell death of cancer cells	8.29E-04	ANXA1,APAF1,ATM,CD47,CD74,CFLAR,EIF2AK2,HMGB1,JAK2,LRRK2,MCL1,NFE2L2,NR3C1,PARP14,PRNP,RAF1,SELL,TNFSF13B	18
Cell Death and Survival	cell death of kidney cell lines	8.85E-04	APOBEC3B,CDC42,CFLAR,DAP3,EIF2AK2,ERN1,JAK2,MCL1,NFE2L2,PPP3R1,PRMT2,PRNP,RAD21,RAF1,RNF13,RNF31,SLK,VCP	18
Cell Death and Survival	cell viability of tumor cell lines	9.22E-04	ATM,CDKL3,CDKN1C,CFLAR,CYBB,DUSP22,EPHB4,GLUD1,GPX1,HMGB1,IGFBP7,MCL1,MTMR1,MTMR6,NFE2L2,NR3C1,PARP14,PPP1CC,PPP2CA,PPP2R5A,PPP4R1,PPP6C,PRNP,PSMA1, RAD21,RAF1,RBBP4,RICTOR,RIT1,RNF31,S100A6,TDP2,TGFBR2,TNFSF13B,TUBB3,VCP	36
Cell Death and Survival	apoptosis of fibroblast cell lines	9.87E-04	APAF1,ATM,ATP6V1G2,CFLAR,CYLD,DDX3X,EIF2AK2,EIF2S1,EPHX1,IFNAR1,MCL1,NFE2L2,NR3C1,PRNP,PSMD6,RBL2,RNF13,STAT1,TAX1BP1	19
Cell Death and Survival, Connective Tissue Disorders, Hematological Disease	hemolytic anemia	9.89E-04	ANXA1,CD47,IFNAR1,NR3C1,PPP3CB,PPP3R1	6
Cancer, Cell Death and Survival, Tumor Morphology	cell death of tumor cells	1.00E-03	ANXA1,APAF1,ATM,CARD8,CD47,CD74,CFLAR,EIF2AK2,HMGB1,JAK2,LRRK2,MCL1,NFE2L2,NR3C1,PARP14,PPP2CA,PRNP,RAF1,SELL,TGFBR2,TNFSF13B	21
Cell Death and Survival	cell death of connective tissue cells	1.27E-03	APAF1,ATM,ATP6V1G2,CDC42,CFLAR,CYLD,DDX3X,DDX58,EIF2AK2,EIF2S1,EPHX1,FNTA,GPX1,GZMA,IFNAR1,MCL1,NFE2L2,NR3C1,PRMT2,PRNP,PSMD6,RAF1,RBL2,RECQL,RNF13,SCP2, SLK,STAT1,TAX1BP1,VCP	30
Cell Death and Survival, Cellular Compromise, Neurological Disease, Tissue Morphology	degeneration of nerve ending	1.45E-03	ARNTL,ATM	2
Cell Death and Survival, Respiratory Disease	quantity of apoptotic pneumocytes	1.45E-03	CD36,NFE2L2	2
Cell Death and Survival	cell death of fibroblast cell lines	1.50E-03	APAF1,ATM,ATP6V1G2,CFLAR,CYLD,DDX3X,EIF2AK2,EIF2S1,EPHX1,FNTA,IFNAR1,MCL1,NFE2L2,NR3C1,PRNP,PSMD6,RBL2,RECQL,RNF13,SCP2,STAT1,TAX1BP1,VCP	23
Cell Death and Survival	cell death of thymocytes	1.50E-03	APAF1,ATM,CFLAR,CYLD,GZMA,KLF13,MCL1,NFE2L2,NR3C1,PTPRC	10
Cell Death and Survival	cell death of epithelial cells	1.52E-03	APAF1,APOBEC3B,ATM,CDC42,CFLAR,CTSS,CYLD,DAP3,EIF2AK2,ERN1,HMGB1,IFNGR1,JAK2,MC1R,MCL1,NFE2L2,NR3C1,PPP3R1,PRMT2,PRNP,RAD21,RAF1,RBL2,RNF31,TGFBR2	25
Cell Death and Survival	mitochondrial cell death of tumor cell lines	1.57E-03	DDX58,MCL1,SRI	3
Cell Death and Survival	apoptosis of leukocytes	1.61E-03	ANXA1,ATM,CD47,CDC42,CFLAR,CYBB,CYLD,EIF2AK2,FCER1A,GZMA,HMGB1,IFNGR1,JAK2,KLF13,MCL1,NFE2L2,NR3C1,PPP3CB,PTGER4,PTPRC,RAF1,STAT1,TNFSF13B	23
Cell Death and Survival	apoptosis of tumor cell lines	1.73E-03	APAF1,ATM,BAZ1A,BRAT1,CARD8,CCAR1,CD47,CDC42,CDKN1C,CFLAR,CYLD,DDX58,EIF2AK2,EIF4G2,EPHB4,ERN1,FCER1A,GLRX,GPX1,IGFBP7,JAK2,LMO4,MCL1,MTMR6,NFE2L2,NR3C1, OAS3,PARP14,PHIP,PPP2CA,PRNP,PTPRC,RAB32,RAD21,RAF1,RASSF3,RBL2,RICTOR,RIT1,RNF13,RNF31,RNF5,S100A6,SRI,STAT1,TDP2,TGFBR2,TMSB10/TMSB4X,TNFSF13B,TXNRD1,VCP ,YWHAE	52
Cell Death and Survival, Connective Tissue Disorders, Hematological Disease, Immunological Disease	autoimmune hemolytic anemia	1.78E-03	CD47,NR3C1,PPP3CB,PPP3R1	4
Cell Death and Survival	cell viability of cervical cancer cell lines	1.89E-03	ATM,CDKL3,DUSP22,MCL1,MTMR1,MTMR6,PPP1CC,PPP2CA,PPP2R5A,PPP4R1,PPP6C,RAD21,TGFBR2	13

Categories	Diseases or Functions Annotation	p-Value	Molecules	# Molecules
Cell Death and Survival	cell death of colon cancer cell lines	1.97E-03	APAF1,CCAR1,CDC42,CDKN1C,CFLAR,EPHB4,MCL1,RAF1,RASSF3,SRI,STAT1,TGFBR2,TMSB10/TMSB4X,TXNRD1,VCP,YWHAE	16
Cell Death and Survival	cell viability of leukocytes	2.26E-03	CD47,CD74,CD86,CYLD,ERN1,FCER1A,HMGB1,JAK2,MCL1,MX1,PARP14,PTPRC,RAF1,STAT1,TNFSF13B	15
Cell Death and Survival	killing of natural killer cells	2.42E-03	CD244,IFNAR1,IFNGR1,STAT1	4
Cell Death and Survival	cell death of myeloid cells	2.46E-03	ANXA1,CFLAR,CYBB,EIF2AK2,FCER1A,HMGB1,IFNAR1,JAK2,MCL1,NFE2L2,NR3C1,RAF1,STAT1	13
Cell Death and Survival	cell death of epithelial cell lines	2.68E-03	APOBEC3B,CDC42,CFLAR,DAP3,EIF2AK2,ERN1,JAK2,MCL1,NFE2L2,PPP3R1,PRMT2,PRNP,RAD21,RAF1,RNF31,TGFBR2	16
Cell Death and Survival	necrosis of epithelial tissue	2.81E-03	APAF1,APOBEC3B,ATM,CD36,CDC42,CFLAR,CTSS,CYLD,DAP3,EIF2AK2,ERN1,GPX1,HMGB1,IFNGR1,JAK2,MC1R,MCL1,NFE2L2,NR3C1,PPP3R1,PRMT2,PRNP,PSMB8,RAD21,RAF1,RBL2,RNF31,TGFBR2	28
Cell Death and Survival	cell viability of blood cells	2.85E-03	CD47,CD74,CD86,CYLD,ERN1,FCER1A,HMGB1,JAK2,MCL1,MX1,PARP14,PTPRC,RAF1,SELE,STAT1,TNFSF13B	16
Cell Death and Survival	cytotoxicity	2.87E-03	CALM1 (includes others),CD244,CD46,CD74,CFLAR,CYBB,FKBP1A,GZMA,HLA-DRB1,IFNAR1,MICA,PTPRC,STAT1,TGFBR2	14
Cell Death and Survival, Embryonic Development	cell death of embryonic cell lines	2.97E-03	APOBEC3B,CDC42,CFLAR,DAP3,EIF2AK2,ERN1,IFNAR1,MCL1,NFE2L2,PPP3R1,PRMT2,PRNP,RAD21,RNF31	14
Cancer, Cell Death and Survival, Tumor Morphology	cell death of leukemia cells	3.42E-03	ATM,CD47,JAK2,MCL1,RAF1,SELL,TNFSF13B	7
Cell Death and Survival, Cellular Compromise	cytotoxicity of cells	3.51E-03	CALM1 (includes others),CD244,CD46,CD74,CFLAR,CYBB,GZMA,HLA-DRB1,IFNAR1,MICA,PTPRC,STAT1,TGFBR2	13
Cell Death and Survival	killing of lymphocytes	3.74E-03	CD244,CD47,IFNAR1,IFNGR1,STAT1	5
Cell Death and Survival	apoptosis of fibroblasts	3.99E-03	APAF1,ATM,CDC42,CFLAR,DDX58,EIF2AK2,EIF2S1,GZMA,MCL1,PRMT2,RAF1,SLK	12
Cell Death and Survival	cell death of fibroblasts	4.36E-03	APAF1,ATM,CDC42,CFLAR,DDX58,EIF2AK2,EIF2S1,EPHX1,GPX1,GZMA,MCL1,PRMT2,RAF1,SLK	14
Cell Death and Survival, Cell Signaling	activation of caspase	4.56E-03	APAF1,CARD8,JAK2,MTCH1,STAT1,VCP	6
Cancer, Cell Death and Survival, Tumor Morphology	cell viability of cancer cells	4.64E-03	CD74,HMGB1,MCL1,NFE2L2,PARP14,SELL,TNFSF13B	7
Cancer, Cell Death and Survival, Tumor Morphology	cell death of chronic lymphocytic leukemia cells	4.68E-03	ATM,CD47,SELL,TNFSF13B	4
Cell Death and Survival	cytolysis of lymphoblastoid cell lines	4.70E-03	CD244,MICA	2
Cell Death and Survival	loss of B lymphocytes	4.70E-03	CD74,SPPL2A	2
Cell Death and Survival	cell death of T lymphocytes	5.03E-03	APAF1,ATM,CD47,CDC42,CFLAR,CYLD,GZMA,IFNGR1,KLF13,MCL1,NFE2L2,NR3C1,PPP3CB,PTPRC,STAT1,TGFBR2	16
Cancer, Cell Death and Survival, Tumor Morphology	cell viability of tumor cells	5.07E-03	CD74,HMGB1,LRRK2,MCL1,NFE2L2,PARP14,SELL,TNFSF13B	8

Categories	Diseases or Functions Annotation	p-Value	Molecules	# Molecules
Cell Death and Survival	cell death of mononuclear leukocytes	5.35E-03	APAF1,ATM,CD47,CDC42,CFLAR,CYLD,EIF2AK2,FCER1A,GZMA,IFNGR1,KLF13,MCL1,NFE2L2,NR3C1,PPP3CB,PTPRC,STAT1,TGFBR2,TNFSF13B	19
Cell Death and Survival	apoptosis of colon cancer cell lines	5.39E-03	APAF1,CCAR1,CDC42,CDKN1C,CFLAR,EPHB4,MCL1,RASSF3,SRI,STAT1,TMSB10/TMSB4X,VCP,YWHAE	13
Cell Death and Survival	cell death of phagocytes	5.46E-03	ANXA1,CFLAR,CYBB,EIF2AK2,FCER1A,HMGB1,IFNAR1,MCL1,NFE2L2,NR3C1,PTGER4,STAT1	12
Cell Death and Survival	apoptosis of myeloid cells	5.60E-03	ANXA1,CFLAR,CYBB,EIF2AK2,FCER1A,HMGB1,JAK2,MCL1,NFE2L2,RAF1,STAT1	11
Cell Death and Survival	apoptosis of thymocytes	6.47E-03	ATM,CFLAR,CYLD,GZMA,KLF13,NFE2L2,NR3C1,PTPRC	8
Cell Death and Survival	apoptosis of kidney cell lines	6.50E-03	CFLAR,DAP3,EIF2AK2,JAK2,MCL1,NFE2L2,PPP3R1,PRMT2,RAD21,RAF1,RNF13,RNF31,SLK	13
Cell Death and Survival	necroptosis of tumor cell lines	6.94E-03	CYLD,STAT1	2
Cell Death and Survival, DNA Replication, Recombination, and Repair	fragmentation of DNA fragment	7.93E-03	APOBEC3B,EIF2AK2,GZMA,MTCH1,PPP1CC	5
Cell Death and Survival	apoptosis of peritoneal macrophages	8.07E-03	CFLAR,MCL1,NFE2L2	3
Cancer, Cell Death and Survival, Tumor Morphology	apoptosis of leukemia cells	8.20E-03	ATM,JAK2,MCL1,RAF1,SELL,TNFSF13B	6
Cell Death and Survival	cell death of lymphocytes	8.22E-03	APAF1,ATM,CD47,CDC42,CFLAR,CYLD,EIF2AK2,GZMA,IFNGR1,KLF13,MCL1,NFE2L2,NR3C1,PPP3CB,PTPRC,STAT1,TGFBR2,TNFSF13B	18
Cancer, Cell Death and Survival, Tumor Morphology	cell viability of chronic lymphocytic leukemia cells	9.34E-03	CD74,SELL,TNFSF13B	3
Cell Death and Survival	cell death of hematopoietic cells	9.40E-03	APAF1,ATM,CFLAR,CYLD,EIF2AK2,GZMA,KLF13,MCL1,NFE2L2,NR3C1,PTPRC,RAF1	12
Cell Death and Survival, Cellular Compromise, Neurological Disease, Organismal Injury and Abnormalities, Tissue Morphology	neurodegeneration of granule cells	9.58E-03	ATM,PRNP	2
Cell Death and Survival	apoptosis of epithelial cell lines	9.98E-03	CFLAR,DAP3,EIF2AK2,JAK2,MCL1,NFE2L2,PPP3R1,PRMT2,RAD21,RAF1,RNF31,TGFBR2	12
Cell Death and Survival	apoptosis of lymphoma cell lines	1.05E-02	CARD8,CFLAR,EIF2AK2,ERN1,FCER1A,IGFBP7,JAK2,MCL1,PTPRC,TNFSF13B	10
Cell-To-Cell Signaling and Interaction, Hematological System Development and Function, Immune Cell Trafficking, Inflammatory Response	activation of leukocytes	6.10E-08	ANXA1,ATM,BLOC1S6,CD244,CD36,CD46,CD47,CD58,CD74,CD86,CLEC7A,CTSS,CYBB,DDX58,ERAP1,FKBP1A,GZMA,HBP1,HLA-DMA,HLA-DMB,HLA-DQA1,HLA-DRB1,HMGB1,IFNAR1,JAK2,MGST1,MICA,NBR1,NDFIP1,NFE2L2,PELI1,PPP3CB,PRNP,PSMB8,PTGER4,PTPRC,RAB10,RAB32,RAB6A,RAB8B,SELE,SELL,STAT1,TGFBR2,TNFSF13B	45

Categories	Diseases or Functions Annotation	p-Value	Molecules	# Molecules
Cell-To-Cell Signaling and Interaction, Hematological System Development and Function	activation of blood cells	1.61E-07	ANXA1,ATM,BLOC1S6,CD244,CD36,CD46,CD47,CD58,CD74,CD86,CLEC7A,CTSS,CYBB,DDX58,ERAP1,FKBP1A,GZMA,HBP1,HLA-DMA,HLA-DMB,HLA-DQA1,HLA-DRB1,HMGB1,IFNAR1,JAK2,MGST1,MICA,NBR1,NDFIP1,NFE2L2,PELI1,PLEK,PPP3CB,PRNP,PSMB8,PTGER4,PTPRC,RAB10,RAB32,RAB6A,RAB8B,SELE,SELL,STAT1,TGFBR2,TNFSF13B	46
Cell-To-Cell Signaling and Interaction	activation of cells	4.70E-07	ANXA1,ATM,BLOC1S6,CD244,CD36,CD46,CD47,CD58,CD74,CD86,CLEC7A,CTSS,CYBB,CYLD,DDX58,EIF2AK2,ERAP1,ERN1,FCER1A,FKBP1A,FNTA,GPX1,GZMA,HBP1,HLA-DMA,HLA-DMB,HLA-DQA1,HLA-DRB1,HMGB1,IFNAR1,JAK2,MGST1,MICA,MTCH1,NBR1,NDFIP1,NFE2L2,PELI1,PLEK,PPP2CA,PPP3CB,PRNP,PSMB8,PTGER4,PTPRC,RAB10,RAB32,RAB6A,RAB8B,RICTOR,SELE,SELL,STAT1,TGFBR2,TNFSF13B	55
Cell-To-Cell Signaling and Interaction, Hematological System Development and Function, Immune Cell Trafficking, Inflammatory Response	activation of antigen presenting cells	5.45E-07	ATM,CD36,CD47,CD74,CD86,CLEC7A,CTSS,CYBB,ERAP1,HBP1,HLA-DMA,HLA-DMB,HLA-DQA1,HMGB1,IFNAR1,JAK2,PELI1,PRNP,PSMB8,PTGER4,RAB10,RAB32,RAB6A,RAB8B,STAT1	25
Antimicrobial Response, Cell-To-Cell Signaling and Interaction, Connective Tissue Development and Function, Inflammatory Response	antiviral response of fibroblasts	1.08E-05	DDX58,IFNAR1,STAT1	3
Cell-To-Cell Signaling and Interaction, Cellular Movement, Hematological System Development and Function, Immune Cell Trafficking	recruitment of leukocytes	2.61E-05	ANXA1,CD36,CD47,CD74,CLEC7A,DDX58,FCER1A,GLRX,HDC,HMGB1,IFNAR1,ITGA4,LRRK2,NFE2L2,PELI1,PRMT2,RAP1A,ROCK1,SELE,SELL,TGFBR2	21
Cell-To-Cell Signaling and Interaction, Tissue Development	adhesion of blood cells	6.76E-05	ANXA1,ANXA7,CD36,CD46,CD47,CD58,CD74,CDC42,CYBB,GALNT1,GLRX,HMGB1,IFNGR1,ITGA4,JAK2,PAFAH1B1,PTGER4,PTPRC,RICTOR,ROCK1,SELE,SELL,STAT1,TGFBR2	24
Cell-To-Cell Signaling and Interaction, Hematological System Development and Function, Immune Cell Trafficking, Tissue Development	adhesion of immune cells	1.31E-04	ANXA1,CD36,CD46,CD47,CD58,CD74,CDC42,GALNT1,GLRX,HMGB1,IFNGR1,ITGA4,JAK2,PAFAH1B1,PTGER4,PTPRC,RICTOR,ROCK1,SELE,SELL,STAT1,TGFBR2	22
Cell-To-Cell Signaling and Interaction, Hematological System Development and Function, Immune Cell Trafficking, Inflammatory Response	activation of lymphocytes	1.39E-04	ANXA1,BLOC1S6,CD244,CD46,CD47,CD58,CD74,CD86,CLEC7A,DDX58,GZMA,HLA-DRB1,HMGB1,IFNAR1,MGST1,MICA,NBR1,NDFIP1,NFE2L2,PELI1,PPP3CB,PRNP,PTPRC,STAT1,TGFBR2,TNFSF13B	26
Cell-To-Cell Signaling and Interaction, Hematological System Development and Function, Immune Cell Trafficking, Tissue Development	adhesion of mononuclear leukocytes	2.18E-04	ANXA1,CD47,CD58,HMGB1,IFNGR1,ITGA4,JAK2,RICTOR,ROCK1,SELE,SELL,TGFBR2	12
Cell-To-Cell Signaling and Interaction, Hematological System Development and Function, Immune Cell Trafficking, Inflammatory Response	activation of T lymphocytes	2.25E-04	ANXA1,CD244,CD46,CD47,CD58,CD74,CD86,CLEC7A,DDX58,GZMA,HLA-DRB1,IFNAR1,MGST1,NBR1,NDFIP1,NFE2L2,PPP3CB,PRNP,PTPRC,STAT1,TGFBR2	21

Categories	Diseases or Functions Annotation	p-Value	Molecules	# Molecules
Cell-To-Cell Signaling and Interaction, Cell-mediated Immune Response, Cellular Movement, Hematological System Development and Function, Immune Cell Trafficking, Tissue Development	adhesion of T lymphocytes	3.06E-04	ANXA1,CD47,CD58,IFNGR1,ITGA4,JAK2,RICTOR,SELE,SELL	9
Cell-To-Cell Signaling and Interaction, Inflammatory Response	response of phagocytes	3.84E-04	ABCA7,ANXA1,CD36,CD47,CLEC7A,DDX58,ERN1,FCER1A,GLRX,HMGB1,IFNAR1,ITGA4,NR3C1,ZEB2	14
Cell-To-Cell Signaling and Interaction, Hematological System Development and Function, Immune Cell Trafficking, Tissue Development	adhesion of lymphocytes	4.20E-04	ANXA1,CD47,CD58,IFNGR1,ITGA4,JAK2,RICTOR,ROCK1,SELE,SELL	10
Cell-To-Cell Signaling and Interaction, Cellular Function and Maintenance, Hematological System Development and Function, Inflammatory Response	phagocytosis of granulocytes	4.51E-04	ANXA1,CD36,CD47,GLRX,HMGB1,IFNAR1	6
Cell-To-Cell Signaling and Interaction, Connective Tissue Development and Function	response of fibroblasts	4.52E-04	CD36,DDX58,IFNAR1,STAT1	4
Cell-To-Cell Signaling and Interaction, Cellular Function and Maintenance, Hematological System Development and Function, Inflammatory Response	phagocytosis of bone marrow-derived neutrophils	4.91E-04	CD47,HMGB1	2
Cardiovascular System Development and Function, Cell-To-Cell Signaling and Interaction, Tissue Development	adhesion of endothelial cells	5.03E-04	ANXA7,CD36,CDC42,HMGB1,IGFBP7,ITGA4,RICTOR,SELE,SELL,TGFBR2,TMSB10/TMSB4X	11
Cell-To-Cell Signaling and Interaction	response of myeloid cells	5.49E-04	ABCA7,ANXA1,CD36,CD47,DDX58,ERN1,FCER1A,GLRX,HMGB1,IFNAR1,ITGA4,NR3C1,PTPRC	13
Cell-To-Cell Signaling and Interaction, Hematological System Development and Function, Immune Cell Trafficking, Tissue Development	detachment of leukocytes	8.24E-04	ANXA1,PTPRC,SELL	3
Cell-To-Cell Signaling and Interaction, Inflammatory Response	immune response of leukocytes	8.55E-04	ABCA7,ANXA1,CD36,CD47,CD86,CTSS,DDX58,ERN1,FCER1A,GLRX,HMGB1,IFNAR1,IFNGR1,ITGA4,NR3C1,PTPRC,TGFBR2	17
Cardiovascular System Development and Function, Cell-To-Cell Signaling and Interaction, Tissue Development	adhesion of vascular endothelial cells	9.38E-04	ANXA7,CD36,HMGB1,IGFBP7,ITGA4,RICTOR,SELE,TMSB10/TMSB4X	8

Categories	Diseases or Functions Annotation	p-Value	Molecules	# Molecules
Cell-To-Cell Signaling and Interaction, Inflammatory Response	immune response of phagocytes	1.30E-03	ABCA7,ANXA1,CD36,CD47,DDX58,ERN1,FCER1A,GLRX,HMGB1,IFNAR1,ITGA4,NR3C1	12
Cell-To-Cell Signaling and Interaction	response of granulocytes	1.33E-03	ANXA1,CD36,CD47,FCER1A,GLRX,HMGB1,IFNAR1,ITGA4	8
Cell-To-Cell Signaling and Interaction, Hematological System Development and Function, Inflammatory Response	immune response of neutrophils	1.37E-03	CD36,CD47,FCER1A,GLRX,HMGB1,IFNAR1,ITGA4	7
Cell-To-Cell Signaling and Interaction	response of antigen presenting cells	1.42E-03	ABCA7,ANXA1,CD36,CD47,CLEC7A,CTSS,DDX58,ERN1,HMGB1,IFNAR1,NR3C1	11
Cell-To-Cell Signaling and Interaction, Cellular Movement, Hematological System Development and Function, Immune Cell Trafficking	recruitment of mononuclear leukocytes	1.80E-03	CD47,DDX58,FCER1A,HDC,HMGB1,ITGA4,PELI1,SELE,SELL	9
Cell-To-Cell Signaling and Interaction, Inflammatory Response	immune response of antigen presenting cells	2.03E-03	ABCA7,ANXA1,CD36,CD47,CTSS,DDX58,ERN1,HMGB1,IFNAR1,NR3C1	10
Cell-To-Cell Signaling and Interaction, Hematological System Development and Function	binding of B-lymphocyte derived cell lines	2.05E-03	CD47,CRKL,ITGA4	3
Cell Cycle, Cell-To-Cell Signaling and Interaction, Cellular Growth and Proliferation	contact growth inhibition of leukocytes	2.05E-03	HMGB1,IKZF1,PTPRC	3
Cell-To-Cell Signaling and Interaction, Cellular Function and Maintenance, Hematological System Development and Function, Inflammatory Response	phagocytosis of neutrophils	2.53E-03	CD36,CD47,GLRX,HMGB1,IFNAR1	5
Cell-To-Cell Signaling and Interaction, Hematological System Development and Function, Immune Cell Trafficking, Tissue Development	cell-cell adhesion of leukocytes	2.80E-03	ITGA4,PTPRC,ROCK1,SELE	4
Cardiovascular System Development and Function, Cell-To-Cell Signaling and Interaction, Tissue Development	adhesion of HCAEC cells	2.86E-03	SELE,SELL	2

Categories	Diseases or Functions Annotation	p-Value	Molecules	# Molecules
Cell-To-Cell Signaling and Interaction, Cellular Compromise, Tissue Development, Tumor Morphology	adhesion of acute myeloid leukemia blast cells	2.86E-03	SELE,SELL	2
Cell-To-Cell Signaling and Interaction, Nervous System Development and Function	afterhyperpolarization of central nervous system cells	2.86E-03	ATP2B4,LMO4	2
Cell-To-Cell Signaling and Interaction, Cellular Movement, Hematological System Development and Function, Immune Cell Trafficking, Tissue Development	detachment of phagocytes	2.86E-03	ANXA1,PTPRC	2
Cell-To-Cell Signaling and Interaction, Cell-mediated Immune Response, Cellular Movement, Hematological System Development and Function, Immune Cell Trafficking	recruitment of CD8+ T lymphocyte	2.86E-03	DDX58,PELI1	2
Cell Cycle, Cell-To-Cell Signaling and Interaction, Cellular Growth and Proliferation	contact growth inhibition	3.09E-03	CDC42,GBP2,HMGB1,IKZF1,ING4,JAK2,PTPRC,RAF1,RBL2,STAT1	10
Cell-To-Cell Signaling and Interaction, Hematological System Development and Function, Immune Cell Trafficking, Infectious Disease, Tissue Development	fusion of leukocytes	3.21E-03	CD36,CD46,CD47,STAT1	4
Cell-To-Cell Signaling and Interaction, Inflammatory Response	response of macrophages	3.89E-03	ABCA7,ANXA1,CD36,CD47,CLEC7A,DDX58,ERN1,HMGB1,NR3C1	9
Cell-To-Cell Signaling and Interaction, Cellular Function and Maintenance, Inflammatory Response	phagocytosis of cells	4.10E-03	ABCA7,ANXA1,CD302,CD36,CD47,CDC42,CLEC7A,CLTC,DNTTIP1,GLRX,HMGB1,IFNAR1,LY75,NR3C1	14
Cell-To-Cell Signaling and Interaction	binding of blood cells	4.16E-03	CD47,CD58,CD86,HLA-DMA,HMGB1,IFNGR1,ITGA4,JAK2,NFE2L2,PTPRC,RAP1B,SELE,SELL	13
Cell-To-Cell Signaling and Interaction, Hematological System Development and Function, Tissue Development	binding of T lymphocytes	4.25E-03	CD47,CD58,CD86,HLA-DMA,IFNGR1,ITGA4	6

Categories	Diseases or Functions Annotation	p-Value	Molecules	# Molecules
Cell-To-Cell Signaling and Interaction, Hematological System Development and Function, Immune Cell Trafficking, Inflammatory Response, Tissue Development	adhesion of monocytes	4.49E-03	HMGB1,ROCK1,SELE,SELL,TGFBR2	5
Cell-To-Cell Signaling and Interaction, Cell-mediated Immune Response, Cellular Movement, Hematological System Development and Function, Immune Cell Trafficking	recruitment of T lymphocytes	4.56E-03	CD47,DDX58,FCER1A,HDC,PELI1,SELE	6
Cell-To-Cell Signaling and Interaction, Nervous System Development and Function	afterhyperpolarization of neurons	4.70E-03	ATP2B4,LMO4	2
Cell Cycle, Cell-To-Cell Signaling and Interaction, Cellular Growth and Proliferation	contact growth inhibition of melanoma cell lines	4.70E-03	RAF1,STAT1	2
Cell-To-Cell Signaling and Interaction, Cellular Growth and Proliferation, Hematological System Development and Function	suppression of effector T lymphocytes	4.70E-03	CD46,CD86	2
Cell-To-Cell Signaling and Interaction, Hematological System Development and Function	binding of mononuclear leukocytes	5.07E-03	CD47,CD58,CD86,HLA-DMA,IFNGR1,ITGA4,SELE,SELL	8
Cell-To-Cell Signaling and Interaction, Hematological System Development and Function	binding of lymphocytes	5.22E-03	CD47,CD58,CD86,HLA-DMA,IFNGR1,ITGA4,SELL	7
Cell-To-Cell Signaling and Interaction, Cellular Movement, Hematological System Development and Function, Immune Cell Trafficking	recruitment of lymphocytes	5.52E-03	CD47,DDX58,FCER1A,HDC,PELI1,SELE,SELL	7
Cell-To-Cell Signaling and Interaction, Hematological System Development and Function, Immune Cell Trafficking, Inflammatory Response, Tissue Development	adhesion of phagocytes	5.85E-03	ANXA1,GLRX,HMGB1,ITGA4,PAFAH1B1,ROCK1,SELE,SELL,TGFBR2	9
Cell-To-Cell Signaling and Interaction, Hematological System Development and Function, Hematopoiesis	binding of hematopoietic progenitor cells	5.85E-03	CD47,ITGA4,RAP1B	3

Categories	Diseases or Functions Annotation	p-Value	Molecules	# Molecules
Cell-To-Cell Signaling and Interaction	binding of leukemia cell lines	6.81E-03	CD36,CD47,ITGA4,JAK2,SELL	5
Cell-To-Cell Signaling and Interaction	binding of bone marrow cell lines	6.90E-03	CRKL,ITGA4,RAF1	3
Cell-To-Cell Signaling and Interaction, Cell-mediated Immune Response, Cellular Movement, Hematological System Development and Function, Immune Cell Trafficking, Tissue Development	adhesion of Th1 cells	6.94E-03	ITGA4,SELE	2
Antigen Presentation, Cell-To-Cell Signaling and Interaction, Inflammatory Response	antigen presentation of antigen presenting cells	6.94E-03	CTSS,IFNAR1	2
Cell-To-Cell Signaling and Interaction, Hematological System Development and Function, Immune Cell Trafficking, Tissue Development	detachment of myeloid cells	6.94E-03	ANXA1,PTPRC	2
Cell-To-Cell Signaling and Interaction, Inflammatory Response	response of mast cells	6.94E-03	FCER1A,ZEB2	2
Cell-To-Cell Signaling and Interaction, Inflammatory Response	immune response of macrophages	7.11E-03	ABCA7,ANXA1,CD36,CD47,DDX58,ERN1,HMGB1,NR3C1	8
Cell-To-Cell Signaling and Interaction	recognition of cells	7.26E-03	CD36,CD47,CLEC7A,MICA	4
Cell-To-Cell Signaling and Interaction, Hematological System Development and Function, Immune Cell Trafficking, Inflammatory Response	activation of phagocytes	7.34E-03	ANXA1,ATM,CD36,CD47,CD86,CLEC7A,CYBB,FKBP1A,GZMA,HMGB1,IFNAR1,JAK2,PELI1,PRNP,PTGER4,SELE,SELL,STAT1	18
Cell-To-Cell Signaling and Interaction, Hematological System Development and Function, Humoral Immune Response	binding of B lymphocytes	8.07E-03	CD47,CD86,ITGA4	3
Cardiovascular System Development and Function, Cell-To-Cell Signaling and Interaction, Tissue Development	adhesion of postcapillary venule	9.58E-03	CD74,SELL	2
Cell Cycle, Cell-To-Cell Signaling and Interaction, Cellular Growth and Proliferation	contact growth inhibition of lymphocytes	9.58E-03	IKZF1,PTPRC	2

Categories	Diseases or Functions Annotation	p-Value	Molecules	# Molecules
Cell-To-Cell Signaling and Interaction, Cellular Function and Maintenance, Inflammatory Response	phagocytosis of blood cells	1.06E-02	ABCA7,ANXA1,CD36,CD47,CDC42,GLRX,HMGB1,IFNAR1	8
Cell-To-Cell Signaling and Interaction, Cellular Function and Maintenance, Inflammatory Response	phagocytosis of phagocytes	1.14E-02	ABCA7,ANXA1,CD36,CD47,GLRX,HMGB1,IFNAR1	7
Cell-To-Cell Signaling and Interaction	fusion of cells	1.15E-02	ANXA1,CD36,CD46,CD47,IFRD1,ROCK1,STAT1,UBE2B	8
