# Supplementary material for: Intracerebral Hemorrhage and Ischemic Stroke of Different Etiologies Have Distinct Alternatively Spliced mRNA Profiles in the Blood: a Pilot RNA-seq Study
Source: Transl Stroke Res. 2015 May 22;6(4):284–9. doi: 10.1007/s12975-015-0407-9 (PMC4485700; doi:10.1007/s12975-015-0407-9)
Supplement: Supplementary file 10 — Raw sequencing count for the 308 exons displaying differential exon usage for Ischemic Stroke (Cardioembolic, Large Vessel, Lacunar), ICH and Controls. (PDF 53 kb) [file 12975_2015_407_MOESM6_ESM.pdf]

Supplementary Table 6. Raw Sequencing Count for the 308 Exons Displaying Differential Exon Usage.

IS, Ischemic Stroke; CE, Cardioembolic; LV, Large Vessel; Lac, Lacunar; ICH, Intracerebral Hemorrhage

	IS_CE		IS_LV		IS_Lac		ICH		CONTROL	
MarkerID	Ave	SD	Ave	SD	Ave	SD	Ave	SD	Ave	SD
chr1.10509776-10510379>APITD1andCORT	6	4	10	2	5	5	9	2	3	1
chr1.112991564-112991794>CTTNBP2NL	9	6	3	2	7	3	16	2	6	7
chr1.114499947-114500540>wawleybo	8	2	1	1	2	2	10	4	5	4
chr1.145509166-145509612>RBM8A.1	621	222	709	157	871	252	1091	118	670	177
chr1.145790974-145791170>GPR89A	89	54	134	59	139	77	169	51	114	22
chr1.150778337-150778492>CTSK	72	33	99	29	106	39	134	12	61	11
chr1.150939858-150940190>LASS2	1110	520	1136	208	1273	225	1804	164	1206	445
chr1.154928545-154928780>SHC1andPYGO2andPBXIP1	428	207	428	206	453	90	573	95	449	120
chr1.155691308-155691471>DAP3	457	95	571	174	667	328	584	106	469	91
chr1.160580214-160580588>SLAMF1	209	100	332	129	371	208	455	139	250	85
chr1.161196029-161196394>TOMM40L	27	19	36	14	50	25	67	31	36	15
chr1.168262382-168262516>SFT2D2andTBX19	12	6	12	7	18	14	23	10	17	12
chr1.17056-17742>WASH7P	3016	638	3539	1337	4029	1435	3832	842	3175	389
chr1.180049625-180049796>CEP350	44	29	38	7	59	29	79	7	33	14
chr1.180049652-180049796>CEP350	44	29	38	7	59	29	78	7	33	14
chr1.19470474-19470585>UBR4	103	65	85	24	139	65	178	53	123	47
chr1.201780731-201780885>NAV1	5	6	7	5	15	12	17	8	10	5
chr1.214836934-214837426>CENPF	16	5	36	5	15	10	13	8	24	7
chr1.235956803-235956912>LYST	105	61	98	14	96	39	201	14	86	54
chr1.243419358-243419542>SDCCAG8	46	11	51	10	41	12	80	17	31	10
chr1.243652316-243652442>SDCCAG8	40	9	42	10	46	22	61	18	35	18
chr1.246729640-246730091>CNST	29	5	32	4	38	27	42	25	31	13
chr1.26799700-26800018>HMGN2	2811	610	3552	1108	4077	2183	3063	773	2961	621
chr1.27431807-27432578>SLC9A1	215	75	227	78	228	95	262	27	248	50
chr1.45987501-45987609>PRDX1	368	173	502	141	578	220	684	195	418	168
chr1.46467098-46468407>MAST2	5	2	2	3	5	3	14	3	4	4
chr1.46805848-46806591>NSUN4andFAAH	27	19	33	9	46	25	62	10	30	14
chr1.53416427-53416558>SCP2	44	31	49	23	61	13	85	20	75	22
chr1.63269390-63269533>ATG4C	51	19	59	18	53	23	100	23	50	18
chr1.78207302-78207433>USP33	76	46	85	46	63	21	170	15	66	38
chr1.85039599-85040103>CTBSandGNG5	348	150	317	214	299	80	383	104	393	66
chr1.85127881-85128058>SSX2IP	26	2	32	16	22	19	47	15	14	6
chr1.86861716-86861978>ODF2L	20	3	32	4	29	30	31	12	21	6

chr1.89271574-89271700>PKN2	152	65	96	35	127	33	190	32	91	34
chr2.101606718-101606908>NPAS2	8	5	2	3	5	3	6	2	10	5
chr2.101627502-101628002>TBC1D8	102	29	142	19	140	28	190	45	112	6
chr2.110584278-110584424>RGPD5	24	9	30	14	34	10	66	6	38	18
chr2.111302237-111302383>RGPD6	24	9	30	14	34	10	65	7	38	18
chr2.113175261-113175491>RGPD8	22	15	17	12	39	30	50	18	37	16
chr2.118864235-118864479>INSIG2	106	49	107	48	86	21	196	19	74	42
chr2.119988299-119988610>STEAP3	9	6	12	10	14	8	16	6	11	5
chr2.160143094-160143317>WDSUB1	20	6	12	4	17	6	15	6	17	7
chr2.172848099-172848599>HAT1	190	50	197	117	223	94	310	77	138	23
chr2.173420100-173420447>PDK1	2	3	9	4	1	2	4	2	3	3
chr2.179463448-179463831>CCDC141andTTN	20	9	8	5	6	2	9	3	3	3
chr2.17953901-17954051>GEN1	7	4	8	3	9	6	15	3	4	5
chr2.182339687-182340015>ITGA4	264	123	327	51	288	107	548	183	296	76
chr2.198175302-198175503>ANKRD44	178	74	147	36	213	125	245	8	147	27
chr2.20756227-20757428>dawgorbu	3	3	6	1	1	2	3	2	1	1
chr2.208446079-208446884>FAM119A	57	26	64	26	91	102	95	17	56	15
chr2.231663444-231663879>CAB39	722	395	519	131	655	244	1300	161	753	477
chr2.234112772-234113219>INPP5D	2551	1156	2598	1340	3182	1600	2276	256	2785	653
chr2.242282407-242282508>SEPT2	531	302	669	186	653	245	893	185	547	92
chr2.242611606-242612016>ATG4B	540	200	556	102	707	110	689	153	656	54
chr2.243168539-243168819>samemo	33	8	60	19	84	49	68	31	89	41
chr2.25258142-25260098>LOC729723	14	8	8	4	6	2	8	5	10	10
chr2.29258330-29258510>FAM179A	53	23	44	9	44	31	80	31	60	36
chr2.73957016-73957156>TPRKB	50	38	46	23	45	37	50	9	29	4
chr2.88336462-88336570>KRCC1	76	38	76	66	72	17	164	26	45	24
chr3.122283274-122283460>DTX3L	206	75	241	103	185	88	280	58	162	56
chr3.137963865-137964523>vusmyby	130	50	176	36	210	117	254	65	158	37
chr3.137963930-137964523>ARMC8	130	50	176	36	210	117	254	65	158	37
chr3.137963930-137964524>ARMC8	130	50	176	36	210	117	254	65	158	37
chr3.150280329-150280447>EIF2A	201	54	230	89	238	87	319	65	183	31
chr3.15778540-15778740>ANKRD28	30	13	33	13	32	19	56	4	18	11
chr3.167452594-167452717>PDCD10	48	21	84	69	51	15	138	50	41	33
chr3.188326949-188327339>LPP	85	43	55	27	97	60	70	14	72	19
chr3.20019802-20020396>RAB5A	375	93	393	125	380	96	478	54	391	72
chr3.23929058-23929280>UBE2E1	417	124	532	154	577	284	666	106	419	65
chr3.25637911-25639423>RARB	8	4	4	3	12	13	19	2	10	3
chr3.39162488-39162680>TTC21A	11	4	9	4	38	59	17	5	11	9

chr3.48456585-48456756>PLXNB1	7	4	3	1	4	2	3	2	3	3
chr3.49448633-49449166>myforbo	5	1	8	4	1	1	3	3	1	1
chr3.52385978-52386119>DNAH1	18	16	27	12	24	18	46	16	21	8
chr3.52561845-52561947>NT5DC2	24	30	32	12	46	34	48	27	29	20
chr3.69028819-69028938>C3orf64	22	7	22	7	32	19	36	4	28	20
chr3.81552424-81552865>chordybo	3	2	3	3	4	3	6	1	1	2
chr3.8606070-8609805>LMCD1	11	6	16	10	10	3	9	6	4	2
chr4.122723829-122723948>EXOSC9	86	40	117	54	97	30	124	19	87	15
chr4.122723829-122723983>EXOSC9	86	40	117	54	97	30	124	19	87	15
chr4.15570247-15570813>klawgu	16	8	9	5	4	2	9	5	2	3
chr4.157731989-157732169>PDGFC	24	9	17	11	19	13	40	12	26	5
chr4.175223190-175223337>KIAA1712	21	15	21	8	27	34	45	6	19	8
chr4.40800804-40800921>NSUN7	25	16	14	8	25	5	49	23	21	13
chr4.76874494-76874938>sporsmorby	5	5	6	4	6	2	9	5	2	2
chr5.134343647-134343829>PCBD2andCATSPER3	6	2	3	1	5	3	8	1	3	4
chr5.139929370-139930496>APBB3andSRA1	1091	310	1119	237	1146	350	1405	120	1064	201
chr5.140895496-140896575>DIAPH1	3753	2026	4227	848	4696	1358	5867	1160	5306	1588
chr5.140895875-140896575>DIAPH1	2563	1466	2812	453	3151	962	4181	856	3564	1132
chr5.14381239-14381361>TRIO	19	13	25	9	43	43	50	19	18	4
chr5.145493406-145493874>LARS	335	217	427	110	495	91	660	192	473	249
chr5.156821041-156822687>ADAM19	1699	819	2160	592	3147	2280	2632	918	2261	765
chr5.162902464-162902678>HMMR	4	4	10	2	4	4	4	1	2	2
chr5.176715528-176715926>NSD1	204	66	226	114	238	44	232	22	220	38
chr5.35053745-35054334>fugey	1	1	1	1	0	0	3	0	1	1
chr5.39274505-39274630>FYB	16	7	23	5	16	6	38	20	18	3
chr5.61688639-61688817>DIMT1L	92	34	130	48	123	40	128	30	94	25
chr5.70531277-70532281>goychyby	255	128	222	110	187	115	274	106	108	19
chr5.77656415-77656552>SCAMP1	44	10	50	22	69	55	69	8	49	18
chr6.100023529-100023947>RPS3P5	1	1	9	3	2	2	4	3	1	1
chr6.109248281-109249436>ARMC2	29	14	16	10	29	7	44	14	27	9
chr6.111619174-111619773>slyjey	3	4	11	2	1	1	6	4	4	2
chr6.122792844-122793050>SERINC1	416	239	284	143	298	115	791	55	312	209
chr6.144289727-144290115>PLAGL1andHYMAI	80	48	56	28	86	53	127	23	67	23
chr6.146285293-146285525>SHPRH	4	5	3	2	20	14	9	1	4	3
chr6.146285293-146285559>SHPRH	4	5	3	2	20	14	9	1	4	3
chr6.153291654-153292549>FBXO5	44	13	76	48	88	28	132	34	54	21
chr6.153291660-153292549>FBXO5	44	13	76	48	88	28	132	34	54	21
chr6.153291674-153292549>FBXO5	44	13	76	48	88	28	132	34	54	21

chr6.158088239-158089557>fyjaw	3	2	3	2	2	2	10	4	5	3
chr6.163984476-163984751>QKI	752	302	637	239	735	75	906	101	716	73
chr6.168370462-168372588>MLLT4	324	184	337	67	803	547	338	181	232	96
chr6.3021094-3022352>teyvybo	513	465	438	370	307	183	1015	287	217	106
chr6.30610545-30612432>C6orf134	105	33	110	33	221	169	140	40	127	48
chr6.32806430-32806547>TAP2andHLA-DOB	216	53	254	115	228	48	285	96	205	61
chr6.34360041-34360260>RPS10andNUDT3	267	57	291	128	322	155	281	47	296	94
chr6.37225553-37225749>TBC1D22B	69	21	76	21	65	10	72	14	46	14
chr6.41036580-41036692>C6orf130andUNC5CL	76	29	98	32	108	72	135	32	88	36
chr6.41751200-41751976>PRICKLE4andTOMM6	26	12	17	8	24	9	33	4	16	7
chr6.79664949-79665569>PHIPandTRNAF13P	56	20	29	18	64	72	77	8	35	13
chr7.101475858-101476865>snorkar	2	2	4	1	6	4	6	3	7	4
chr7.142630429-142630905>TRPV5	26	15	5	5	9	6	13	8	5	3
chr7.149598-152547>kehera	53	11	73	18	40	21	84	26	39	9
chr7.158334118-158334468>PTPRN2	7	5	3	5	0	1	1	1	0	1
chr7.2282560-2282683>NUDT1	23	12	31	16	33	13	26	7	27	9
chr7.22980878-22987334>FAM126A	643	157	769	304	904	578	1174	319	601	158
chr7.2635311-2636062>dochuby	6	2	10	4	13	4	23	11	5	4
chr7.29549802-29552165>klerky	3	1	9	4	11	3	15	4	6	3
chr7.45083306-45083697>CCM2	5	3	2	2	3	3	7	2	2	1
chr7.5938415-5938550>CCZ1	101	31	146	37	131	33	171	29	92	27
chr7.74166365-74166897>GTF2I	1391	869	1053	106	1338	433	2228	96	1285	487
chr7.76870183-76870364>CCDC146	4	4	5	4	5	5	8	2	5	5
chr7.99674926-99675056>ZNF3	68	28	86	14	117	30	130	30	83	24
chr8.10340434-10340741>LOC346702	18	7	9	6	11	4	19	7	9	7
chr8.104406853-104407319>shuskeebu	6	3	1	2	2	3	3	2	1	1
chr8.104455023-104455428>DCAF13	87	41	103	65	111	51	152	42	68	31
chr8.133984843-133984986>TG	7	8	6	6	8	10	9	4	4	2
chr8.24256387-24256553>ADAMDEC1	3	2	0	1	4	2	5	2	2	1
chr8.30948350-30948458>WRN	7	6	12	9	14	15	23	5	13	15
chr8.62438536-62438671>ASPH	45	10	45	11	26	16	97	47	34	15
chr8.74858684-74859055>TCEB1	320	106	311	178	331	70	499	71	262	95
chr8.90798887-90799401>RIPK2	82	24	132	28	100	34	154	25	103	26
chr9.131486273-131486409>ZDHHC12	290	90	323	77	298	70	336	74	304	58
chr9.140473077-140473340>WDR85	39	12	54	30	80	60	56	24	43	13
chr9.17135038-17135423>CNTLN	11	6	5	2	3	3	16	3	10	10
chr9.33264164-33264493>CHMP5	2546	991	2766	108	2095	468	1690	330	2570	480
chr9.35737655-35737936>GBA2	633	261	632	116	749	29	888	190	696	257

chr9.46687439-46688197>KGFLP1	42	13	93	48	152	228	66	42	37	21
chr9.95018962-95019082>IARS	87	31	113	43	139	104	132	42	122	33
chr9.96866557-96866667>PTPDC1	5	6	4	4	18	22	12	5	9	2
chr10.11272033-11272456>CELF2	4	1	2	2	2	3	7	2	2	1
chr10.32324818-32324922>KIF5B	105	65	85	42	109	46	230	9	99	52
chr10.38299602-38299711>ZNF33A	108	47	64	16	97	37	112	21	82	23
chr10.38299604-38299711>ZNF33A	108	47	64	16	97	37	112	21	82	23
chr10.46918169-46918362>FAM35BandRHEBP1	8	6	16	3	3	4	9	3	4	4
chr10.49253461-49254183>BMS1P7	12	4	13	4	20	33	12	4	8	6
chr10.51592090-51592619>LOC100287554	325	117	432	118	421	121	495	95	315	45
chr10.69828759-69829524>HERC4	39	6	38	12	47	10	68	11	44	22
chr10.75230828-75230967>PPP3CB	164	51	185	34	227	115	267	65	210	27
chr10.92500578-92502285>HTR7	7	3	11	4	13	4	25	8	16	4
chr10.99195666-99196308>EXOSC1	335	100	437	167	460	198	448	90	360	89
chr10.99433338-99433902>DHDPSLandPI4K2A	205	108	305	96	415	302	339	82	291	87
chr11.111889680-111893310>DIXDC1	15	7	40	20	25	17	59	16	30	8
chr11.111889680-111893374>DIXDC1	15	7	40	20	25	17	59	16	30	8
chr11.119039480-119040011>NLRX1	95	22	81	37	91	28	79	10	73	6
chr11.125490667-125490901>STT3AandCHEK1	262	92	304	129	358	125	332	104	319	85
chr11.47738539-47739064>FNBP4	730	390	907	353	1008	458	1412	384	916	366
chr11.61129205-61129720>CYBASC3	232	128	320	57	436	251	398	118	313	40
chr11.62105383-62105784>saroro	69	33	102	17	57	29	115	59	88	22
chr11.62389338-62389648>B3GAT3	127	21	191	107	192	84	152	38	172	45
chr11.62475067-62475387>GNG3	5	4	16	7	11	6	14	9	6	2
chr11.6523983-6524156>FXC1andDNHD1	19	6	17	15	42	34	38	13	19	11
chr11.7479027-7479174>veemee	11	8	10	10	10	15	21	10	9	6
chr11.836251-836525>CD151	483	184	659	162	424	221	1049	194	550	220
chr11.89933252-89935719>CHORDC1	482	127	520	234	618	270	823	164	453	160
chr12.10561988-10562183>KLRC4andKLRK1	94	39	48	30	49	30	146	100	50	38
chr12.111065735-111066029>TCTN1	6	5	10	10	21	14	8	1	8	6
chr12.123262038-123262230>CCDC62	5	3	6	5	12	10	9	4	4	3
chr12.2966630-2968829>FOXM1	415	73	541	240	693	448	535	268	474	83
chr12.40441853-40442012>SLC2A13	4	6	3	5	12	13	8	1	5	5
chr12.48094974-48095387>RPAP3	156	32	190	72	227	93	304	23	194	56
chr12.54645834-54646011>CBX5	50	21	51	14	60	54	70	12	43	25
chr12.54789679-54790160>ITGA5	3367	1273	2978	1467	3515	1725	3419	258	3883	1067
chr12.56334947-56335109>DGKA	495	226	725	314	618	235	651	188	535	184
chr12.58345541-58345678>XRCC6BP1	43	29	57	23	36	12	82	23	39	12

chr12.6761437-6761584>ING4	446	141	516	125	501	201	613	89	449	65
chr12.94914730-94915694>LOC400061	2	1	0	1	5	1	1	1	5	2
chr12.96258857-96259166>SNRPF	250	53	374	132	439	207	373	80	285	92
chr13.100543572-100543866>CLYBL	29	19	44	15	49	27	50	14	42	18
chr13.103506107-103506222>BIVMandERCC5	214	107	248	58	275	112	362	86	221	60
chr13.113864293-113864812>PCID2	142	38	162	53	149	73	133	36	134	23
chr13.41593364-41593568>ELF1	148	69	153	12	188	93	264	43	149	44
chr14.100743755-100744113>YY1	827	350	782	21	917	60	1337	157	891	307
chr14.105236090-105236707>AKT1	3049	1444	3088	682	3806	962	4290	828	3899	771
chr14.19683027-19683434>DUXAP10	4	2	2	2	2	2	8	7	12	6
chr14.20872770-20872931>TEP1	66	31	68	28	104	95	125	18	68	18
chr14.50246313-50246524>KLHDC2	239	90	245	103	249	42	417	46	183	74
chr14.52957557-52957723>TXNDC16	12	15	16	7	16	7	30	8	12	8
chr14.53248502-53248629>GNPNAT1	23	17	40	23	34	9	35	14	27	22
chr14.70242552-70243105>SLC10A1	4	1	1	1	1	1	3	3	0	0
chr14.76107075-76107403>FLVCR2andTTLL5andC14orf179	54	11	88	39	40	23	89	12	57	16
chr14.88431849-88431973>GALC	119	53	134	57	97	30	223	45	138	33
chr14.88452833-88452946>GALC	88	38	94	15	89	4	182	32	84	18
chr14.96795821-96795971>ATG2B	18	10	6	5	32	9	36	11	17	12
chr14.96997812-96999040>PAPOLA	740	296	683	147	770	88	1257	32	732	277
chr15.101847418-101849508>PCSK6	20	8	31	12	17	7	13	8	22	17
chr15.30711214-30711348>rukaru	8	4	7	2	14	10	19	10	5	4
chr15.38619054-38620016>koyzawbu	4	2	2	2	5	5	14	5	9	2
chr15.52970203-52970319>KIAA1370	53	31	39	12	60	39	88	21	39	27
chr15.57545460-57545666>stoyguby	74	50	96	26	128	63	145	47	94	29
chr15.59102429-59102587>FAM63B	13	7	10	4	17	16	26	9	9	7
chr15.59943710-59944525>GTF2A2	333	118	409	148	466	127	537	61	384	84
chr15.64017491-64017712>HERC1	23	18	23	6	35	16	62	9	24	22
chr15.66811217-66811416>ZWILCH	13	7	18	9	17	12	30	9	13	6
chr15.66811217-66811467>ZWILCH	13	7	18	8	17	12	30	9	13	6
chr15.75165540-75165688>SCAMP2	643	145	858	490	942	399	771	154	847	277
chr15.80191177-80191467>ST20andMTHFS	927	644	490	134	622	311	722	153	675	288
chr15.81584265-81585378>IL16	33	14	52	31	67	51	31	22	44	19
chr15.94774950-94775234>MCTP2	3	1	2	2	2	3	4	1	1	1
chr16.15013757-15013940>zoner	7	3	5	2	11	2	9	3	6	2
chr16.18799866-18800440>ARL6IP1andRPS15A	5723	3056	7577	6306	9709	8000	7089	3044	2677	709
chr16.22277711-22277845>EEF2K	18	10	21	9	26	20	35	16	19	5
chr16.30593851-30595166>syrar	133	45	169	61	264	268	179	49	146	56

chr16.3493611-3493837>ZNF174andNAT15andCLUAP1	38	12	35	10	32	10	52	9	39	15
chr16.4475881-4476093>DNAJA3	67	32	75	24	118	75	95	31	81	28
chr16.72146312-72146549>DHX38	324	114	407	195	461	153	386	100	399	76
chr17.18087711-18088067>jeeroy	408	148	499	202	588	313	527	91	478	64
chr17.18486655-18486837>CCDC144B	10	9	4	5	2	1	13	7	3	4
chr17.27581220-27581513>CRYBA1	3	3	5	4	4	5	5	1	4	2
chr17.34856670-34856799>MYO19	48	17	44	13	59	40	62	24	42	12
chr17.36351796-36351996>TBC1D3	544	205	447	151	512	131	595	46	400	66
chr17.40280569-40280818>RAB5C	3510	902	3641	1429	4034	1279	4306	571	4126	461
chr17.42982993-42984756>GFAP	5	7	5	2	8	10	8	3	12	7
chr17.43002077-43003867>KIF18B	9	6	20	8	10	9	13	7	5	3
chr17.57728564-57728677>CLTC	240	123	199	32	227	44	434	52	202	96
chr17.61473104-61473289>TANC2	19	11	6	5	5	4	24	4	10	2
chr17.62745780-62746126>LOC146880	477	195	345	103	370	156	699	39	356	194
chr17.73000302-73002233>CDR2L	13	8	10	4	12	6	6	6	10	4
chr17.77079383-77079672>ENGASE	218	53	259	137	302	110	358	50	231	54
chr18.28642978-28643439>DSC2	8	6	4	2	3	2	7	2	15	8
chr18.33077683-33077895>INO80C	107	45	119	36	192	56	198	74	139	36
chr18.43417478-43417850>SIGLEC15	8	4	8	6	5	3	6	3	9	6
chr18.48443613-48443878>ME2	387	179	453	96	399	76	696	64	402	167
chr18.54318248-54318824>TXNL1	8	7	15	1	15	10	26	6	15	11
chr18.67508480-67516323>DOK6	36	16	67	26	31	11	123	17	21	14
chr19.11411543-11411912>tojaw	844	366	823	525	814	464	978	288	805	569
chr19.13009896-13010199>SYCE2	11	5	10	3	22	11	17	11	11	7
chr19.1877203-1877424>FAM108A1	1180	268	1839	855	1729	826	1323	370	1873	1007
chr19.18959976-18960255>UPF1	12	6	1	1	7	3	14	1	6	3
chr19.1953385-1953505>C19orf34	8	8	4	4	3	4	12	5	7	5
chr19.35173682-35173954>ZNF302	35	12	56	32	75	68	68	25	46	22
chr19.36515246-36515534>CLIP3	12	5	7	6	15	8	11	8	5	4
chr19.39138368-39138547>ACTN4	999	475	927	316	910	195	1233	137	1066	168
chr19.44128266-44128394>CADM4	54	14	35	9	27	9	58	6	43	10
chr19.44619641-44619995>ZNF225	9	8	15	5	16	12	26	11	12	5
chr19.45543176-45543569>SFRS16	259	91	269	76	367	226	324	69	261	41
chr19.47646729-47646862>SAE1	180	88	250	79	257	51	334	108	226	66
chr19.47646751-47646862>SAE1	180	88	250	79	257	51	334	108	226	66
chr19.49314066-49314178>BCAT2	2	3	2	2	6	6	4	3	2	2
chr19.5208248-5208402>PTPRS	24	11	14	4	25	24	29	15	14	7
chr19.52207575-52207733>NCRNA00085	63	47	43	10	51	23	57	13	45	16

chr19.54610118-54610266>NDUFA3	702	105	970	403	1129	450	810	217	905	248
chr19.58423428-58423554>ZNF417andZNF814	48	25	40	10	59	45	69	15	36	16
chr19.58423428-58423557>ZNF417andZNF814	48	25	40	10	59	45	69	15	36	16
chr19.58427747-58427959>ZNF417andZNF814	19	7	16	4	23	10	26	10	18	8
chr19.58427747-58427960>ZNF417andZNF814	19	7	16	4	23	10	26	10	18	8
chr19.8441789-8441951>lyta	26	12	27	22	30	15	47	9	17	5
chr19.9720432-9722012>ZNF562andZNF561	317	120	418	116	570	354	612	142	347	110
chr20.18449588-18449705>POLR3F	16	5	23	13	29	16	39	11	18	9
chr20.23401942-23402097>NAPB	9	6	16	7	20	14	20	2	9	2
chr20.30720816-30720929>TM9SF4	212	56	215	78	276	88	283	67	209	46
chr20.32079185-32079982>spawvor	0	0	1	1	4	1	1	1	1	1
chr20.32880178-32880359>AHCY	215	44	263	111	300	95	260	79	256	55
chr20.33056659-33057236>vytaw	0	1	3	1	0	0	1	1	1	1
chr20.34487292-34487561>PHF20	157	66	150	26	182	24	243	55	159	37
chr20.416929-419485>TBC1D20	1681	616	1725	323	2155	451	2490	466	1947	447
chr20.43808628-43808775>rotora	268	129	180	114	252	47	370	93	242	82
chr20.43995515-43996064>SYS1-DBNDD2	737	222	853	168	907	200	976	122	800	164
chr20.52560545-52561535>BCAS1	36	34	37	32	21	11	41	15	27	11
chr21.40619627-40619758>BRWD1	22	10	21	8	24	17	36	5	10	7
chr21.47608408-47608855>klorley	18	9	19	10	23	7	33	9	25	8
chr21.47706315-47706712>C21orf57	47	11	51	14	82	76	46	17	60	26
chr22.18613610-18614498>PEX26andTUBA8	85	36	128	43	127	20	77	46	112	33
chr22.19115606-19115962>skatee	5	2	1	2	2	1	2	1	4	1
chr22.20093700-20093800>DGCR8	91	65	126	24	95	17	160	60	94	25
chr22.24316496-24316679>GSTTP1andDDT	376	125	543	255	713	435	400	58	442	185
chr22.29141852-29141989>HSCB	24	19	46	23	48	15	74	9	30	16
chr22.31733654-31734031>sneypoy	31	8	27	11	30	9	35	11	27	16
chr22.36892014-36892255>FOXRED2andTXN2	6	6	10	5	22	13	13	5	21	7
chr22.41175013-41175129>SLC25A17	68	31	76	25	99	50	102	24	70	18
chr22.41252435-41253036>ST13	976	160	1107	296	1319	543	940	222	1007	194
chr22.44514918-44515020>PARVB	246	120	351	72	300	59	304	124	286	79
chr22.45254869-45255776>PRR5-ARHGAP8	9	8	16	9	9	12	20	5	8	4
chr22.50320903-50321181>CRELD2	128	82	155	54	208	77	182	37	150	32
chr22.51221467-51221714>RABL2B	98	52	115	36	177	142	150	53	115	54
chrX.118985730-118985836>UPF3B	31	9	36	12	36	16	48	21	21	14
chrX.138864706-138864887>ATP11C	36	10	34	12	65	64	56	12	34	10
chrX.149924161-149924396>MTMR1	139	72	163	29	204	88	250	41	156	43
chrX.152226503-152227128>PNMA3	81	40	131	49	110	47	106	75	82	72

chrX.153744234-153744566>FAM3A	138	30	197	77	197	97	155	44	155	14
chrX.15862547-15863639>AP1S2	1099	341	1258	334	1260	655	1852	239	1006	206
chrX.16870674-16871149>RBBP7	400	208	560	107	616	249	699	255	520	148
chrX.2839944-2840065>ARSD	41	18	53	24	29	12	72	10	45	9
chrX.40495835-40495964>CXorf38	238	60	234	71	224	19	367	40	229	44
chrX.48367956-48368344>PORCN	63	20	83	37	84	59	90	30	94	15
chrX.74282163-74282417>ABCB7	99	35	94	18	136	66	134	48	97	35
chrX.76776266-76776394>ATRX	206	80	211	80	276	197	224	28	165	51
chrX.77303661-77305892>ATP7A	469	209	479	98	475	63	804	67	457	167
