# Supplementary material for: Intracerebral Hemorrhage and Ischemic Stroke of Different Etiologies Have Distinct Alternatively Spliced mRNA Profiles in the Blood: a Pilot RNA-seq Study
Source: Transl Stroke Res. 2015 May 22;6(4):284–9. doi: 10.1007/s12975-015-0407-9 (PMC4485700; doi:10.1007/s12975-015-0407-9)
Supplement: Supplementary file 11 — Differential exon usage for the 308 exons for Large Vessel IS, Cardioembolic IS, Lacunar IS, ICH and Controls (p < 0.0005, FC > |1.2|). (PDF 84 kb) [file 12975_2015_407_MOESM7_ESM.pdf]

SUPPLEMENTARY TABLE 7. Differential Exon Usage in the 5 Groups ($p < 0.005$, FC > |1.2|). FC-Fold Change										
Upregulated in CE IS									
Marker ID	Gene Symbol	CE Stroke vs. Controls		CE Stroke vs. ICH		CE Stroke vs. LV		CE Stroke vs. Lacunar	
		p-value	FC	p-value	FC	p-value	FC	p-value	FC
chr1.86861716-86861978>ODF2L	ODF2L	4.89E-04	32.54	8.33E-02	1.68	4.39E-04	52.79	4.15E-04	77.95
chr10.38299602-38299711>ZNF33A	ZNF33A	1.34E-03	8.75	4.81E-04	>500	3.54E-03	4.52	4.80E-04	Not Estimable
chr10.38299604-38299711>ZNF33A	ZNF33A	1.34E-03	8.75	4.81E-04	>500	3.54E-03	4.52	4.80E-04	Not Estimable
chr10.49253461-49254183>BMS1P7	BMS1P7	3.00E-04	11.83	1.29E-04	>500	2.16E-03	3.62	5.90E-04	6.62
chr14.70242552-70243105>SLC10A1	SLC10A1	3.87E-04	Not Estimable	1.52E-02	2.52	4.05E-03	3.93	7.65E-03	3.10
chr17.34856670-34856799>MYO19	MYO19	6.48E-04	7.89	2.45E-03	3.85	1.19E-03	5.32	4.55E-04	11.01
chr17.73000302-73002233>CDR2L	CDR2L	1.76E-05	10.40	1.03E-05	19.45	1.19E-05	15.70	2.54E-05	7.93
chr19.18959976-18960255>UPF1	UPF1	6.28E-04	4.27	8.00E-05	21.25	4.87E-05	Not Estimable	9.17E-05	16.73

chr19.58427747-58427959>ZNF417andZNF814	ZNF417 and ZNF814	4.40E-04	4.45	3.91E-02	1.64	5.11E-05	31.22	4.31E-05	62.20
chr19.58427747-58427960>ZNF417andZNF814	ZNF417 and ZNF814	4.40E-04	4.45	3.91E-02	1.64	5.11E-05	31.22	4.31E-05	62.20
chr2.119988299-119988610>STEAP3	STEAP3	4.66E-04	199.47	1.78E-03	6.53	4.46E-04	>500	5.08E-04	68.51
chr2.179463448-179463831>CCDC141andTTN	CCDC141 and TTN	4.17E-06	56.83	5.56E-05	4.49	3.38E-06	>500	3.38E-06	>500
chr2.25258142-25260098>LOC729723	LOC729723	1.67E-03	4.92	4.83E-04	13.26	6.86E-04	8.95	5.62E-04	10.97
chr20.43995515-43996064>SYS1-DBNDD2	SYS1-DBNDD2	1.17E-02	9.29	9.88E-02	2.21	2.68E-02	-1.76	3.16E-02	3.81
chr22.19115606-19115962>skatee	skatee	1.69E-02	1.83	6.02E-04	3.72	5.45E-05	16.63	2.53E-04	5.12
chr3.188326949-188327339>LPP	LPP	1.18E-03	9.73	6.73E-04	24.42	5.40E-04	61.39	4.89E-04	196.57
chr3.48456585-48456756>PLXNB1	PLXNB1	1.81E-05	Not Estimable	3.29E-04	4.01	1.81E-05	>500	7.19E-04	3.20

chr3.49448633-49449166>myforbo	myforbo	3.99E-04	5.56	2.06E-04	8.39	8.58E-02	1.50	1.01E-04	19.14
chr4.15570247-15570813>klawgu	klawgu	3.67E-04	7.05	8.78E-03	2.30	6.85E-02	1.58	5.41E-04	5.61
chr6.37225553-37225749>TBC1D22B	TBC1D22B	2.24E-01	1.22	2.12E-02	1.59	1.12E-01	-1.24	7.61E-04	2.56
chr7.142630429-142630905>TRPV5	TRPV5	1.01E-03	4.98	4.08E-03	2.99	1.34E-04	>500	1.68E-04	42.44
chr7.158334118-158334468>PTPRN2	PTPRN2	1.50E-04	Not Estimable	2.64E-04	17.40	3.29E-03	3.27	2.01E-04	33.34
chr8.104406853-104407319>shuskeebu	shuskeebu	9.62E-05	18.92	4.82E-03	2.47	2.53E-04	7.00	1.48E-03	3.32
**Upregulated in LV IS**									
**Marker ID**	**Gene Symbol**	**CE Stroke vs. LV**		**Controls vs. LV**		**ICH vs. LV**		**LV vs. Lacunar**	
		**p-value**	**FC**	**p-value**	**FC**	**p-value**	**FC**	**p-value**	**FC**
chr1.214836934-214837426>CENPF	CENPF	5.00E-04	-3.69	5.93E-02	-1.51	1.21E-03	-2.91	1.98E-04	5.17
chr10.46918169-46918362>FAM35BandRHEBP1	FAM35B and RHEBP1	1.26E-02	-2.69	2.31E-03	-5.33	4.16E-04	<-500	7.30E-04	16.07

chr11.62389338-62389648>B3GAT3	B3GAT3	2.98E-04	-184.49	1.74E-03	-5.23	1.16E-02	-2.57	3.51E-03	3.78
chr12.54645834-54646011>CBX5	CBX5	4.19E-04	-9.67	1.51E-04	<-500	1.53E-04	<-500	7.79E-04	6.06
chr12.56334947-56335109>DGKA	DGKA	3.08E-02	-2.03	3.68E-03	-3.73	4.70E-04	-19.19	9.49E-04	7.91
chr13.103506107-103506222>BIVMandERCC5	BIVM and ERCC5	3.55E-07	-8.46	1.47E-07	-18.25	7.87E-08	-119.86	1.72E-07	15.06
chr14.53248502-53248629>GNPNAT1	GNPNAT1	3.32E-03	-3.53	2.07E-04	<-500	4.12E-04	-13.92	4.11E-02	1.85
chr15.101847418-101849508>PCSK6	PCSK6	2.64E-05	-9.47	1.70E-04	-3.93	1.94E-03	-2.29	1.42E-05	18.68
chr15.81584265-81585378>IL16	IL16	4.03E-04	-7.21	1.56E-02	-2.08	1.94E-03	-3.49	1.93E-04	14.76
chr16.4475881-4476093>DNAJA3	DNAJA3	4.39E-04	-9.19	2.89E-01	-1.28	2.48E-02	-1.98	3.13E-03	3.32
chr16.72146312-72146549>DHX38	DHX38	3.43E-04	<-500	3.58E-04	-207.50	6.13E-04	-15.82	3.43E-04	>500
chr17.43002077-43003867>KIF18B	KIF18B	7.66E-04	-4.84	4.20E-04	-6.67	3.80E-02	-1.75	2.95E-03	3.02

chr2.173420100-173420447>PDK1	PDK1	5.32E-04	-8.27	6.79E-03	-2.69	2.85E-03	-3.49	3.74E-04	11.68
chr2.20756227-20757428>dawgorbu	dawgorbu	5.41E-04	-3.87	2.90E-05	Not Estimable	2.42E-04	-5.27	1.12E-04	8.19
chr20.33056659-33057236>vytaw	vytaw	3.05E-04	-11.22	2.33E-03	-3.50	7.30E-04	-5.73	1.25E-04	Not Estimable
chr20.43995515-43996064>SYS1-DBNDD2	SYS1-DBNDD2	2.68E-02	-1.76	8.54E-05	-16.38	7.51E-04	-3.90	2.23E-04	6.72
chr20.52560545-52561535>BCAS1	BCAS1	1.65E-04	-247.51	3.31E-03	-3.31	6.66E-04	-6.90	3.85E-04	11.11
chr22.18613610-18614498>PEX26andTUBA8	PEX26 and TUBA8	1.36E-02	-2.14	3.42E-04	-8.12	9.20E-04	-4.61	7.57E-02	1.57
chr22.29141852-29141989>HSCB	HSCB	4.31E-03	-3.84	9.81E-03	-2.87	4.12E-04	-167.51	2.88E-03	4.61
chr3.49448633-49449166>myforbo	myforbo	8.58E-02	1.50	1.68E-02	-3.71	8.50E-03	-5.60	3.98E-03	12.77
chr3.8606070-8609805>LMCD1	LMCD1	6.35E-03	-3.28	2.63E-03	-4.75	1.48E-03	-6.74	3.77E-04	Not Estimable

chr4.15570247-15570813>klawgu	klawgu	6.85E-02	1.58	1.98E-02	-4.45	3.12E-01	-1.45	2.90E-02	3.54
chr5.162902464-162902678>HMMR	HMMR	2.07E-04	<-500	1.05E-03	-5.96	1.57E-03	-4.80	4.74E-03	3.13
chr5.176715528-176715926>NSD1	NSD1	3.78E-04	-8.90	1.22E-04	Not Estimable	1.01E-03	-4.81	3.10E-04	10.77
chr5.61688639-61688817>DIMT1L	DIMT1L	1.15E-03	-4.55	2.66E-04	-12.85	5.61E-03	-2.70	1.79E-04	26.01
chr6.100023529-100023947>RPS3P5	RPS3P5	6.84E-05	-7.70	2.96E-05	-17.42	1.92E-04	-4.63	4.47E-05	10.72
chr6.111619174-111619773>slyjey	slyjey	6.80E-05	-6.99	4.07E-04	-3.47	1.65E-02	-1.74	2.30E-05	19.83
chr6.37225553-37225749>TBC1D22B	TBC1D22B	1.12E-01	-1.24	9.78E-03	-1.52	6.80E-04	-1.98	2.94E-05	3.18
chr7.2282560-2282683>NUDT1	NUDT1	5.87E-04	-3.95	3.45E-05	Not Estimable	3.70E-05	-150.96	1.65E-04	7.00
chr8.90798887-90799401>RIPK2	RIPK2	1.00E-04	-18.86	1.65E-04	-10.01	3.30E-02	-1.74	4.32E-04	5.31

chr9.131486273-131486409>ZDHHC12	ZDHHC12	1.41E-05	-6.54	2.13E-05	-5.43	5.90E-06	-11.68	8.83E-06	8.53
chrX.152226503-152227128>PNMA3	PNMA3	3.13E-03	-4.43	4.20E-04	-126.98	5.52E-04	-26.32	1.74E-03	6.14

Upregulated in Lacunar IS									
Marker ID	Gene Symbol	CE Stroke vs. Lacunar		Controls vs. Lacunar		ICH vs. Lacunar		LV vs. Lacunar	
		p-value	FC	p-value	FC	p-value	FC	p-value	FC
chr1.155691308-155691471>DAP3	DAP3	1.44E-03	-5.83	1.51E-02	-2.40	4.78E-04	-18.04	9.64E-04	-7.72
chr1.160580214-160580588>SLAMF1	SLAMF1	9.00E-05	-50.28	6.08E-04	-4.99	8.40E-05	-75.82	1.30E-04	-17.92
chr1.246729640-246730091>CNST	CNST	1.49E-04	<-500	1.49E-04	<-500	5.30E-04	-7.83	1.12E-02	-2.35
chr11.119039480-119040011>NLRX1	NLRX1	2.47E-03	-4.13	8.08E-04	-7.84	5.40E-04	-11.67	3.97E-04	-18.66
chr11.62475067-62475387>GNG3	GNG3	2.61E-02	-2.07	2.51E-04	<-500	8.27E-03	-2.76	6.18E-04	-10.50
chr12.111065735-111066029>TCTN1	TCTN1	2.36E-04	<-500	5.53E-04	-11.15	2.41E-04	-407.21	2.36E-04	<-500
chr12.123262038-123262230>CCDC62	CCDC62	7.34E-03	-3.02	6.85E-04	-12.40	1.61E-03	-5.83	4.55E-04	-27.30

chr12.2966630-2968829>FOXM1	FOXM1	4.11E-04	-4.71	9.88E-04	-3.47	2.51E-03	-2.72	1.60E-04	-7.77
chr12.94914730-94915694>LOC400061	LOC400061	8.26E-04	-2.46	2.57E-01	-1.20	1.49E-05	-9.38	4.11E-06	Not Estimable
chr14.96795821-96795971>ATG2B	ATG2B	1.42E-04	-4.39	5.92E-04	-2.96	1.69E-02	-1.70	6.23E-05	-6.19
chr15.52970203-52970319>KIAA1370	KIAA1370	7.48E-02	-1.68	2.15E-02	-2.19	2.45E-03	-4.32	2.70E-04	<-500
chr16.15013757-15013940>zoner	zoner	8.81E-03	-2.19	3.72E-02	-1.70	2.46E-03	-2.90	5.61E-05	Not Estimable
chr18.33077683-33077895>INO80C	INO80C	1.26E-03	-4.39	3.20E-04	-10.51	6.38E-02	-1.64	2.82E-03	-3.28
chr19.35173682-35173954>ZNF302	ZNF302	5.42E-04	-24.66	7.08E-04	-14.32	4.23E-04	-74.20	4.45E-04	-52.73
chr2.101627502-101628002>TBC1D8	TBC1D8	2.60E-04	-13.65	1.09E-03	-4.69	1.25E-04	<-500	3.07E-04	-11.16
chr2.160143094-160143317>WDSUB1	WDSUB1	1.02E-03	-8.14	3.24E-04	Not Estimable	6.23E-03	-3.18	2.45E-03	-4.63

chr2.29258330-29258510>FAM179A	FAM179A	2.28E-05	<-500	7.40E-05	-9.36	5.57E-05	-12.23	4.93E-03	-2.20
chr20.32079185-32079982>spawvor	spawvor	1.44E-05	Not Estimable	1.47E-04	-5.01	3.41E-04	-3.73	6.92E-05	-7.31
chr21.47706315-47706712>C21orf57	C21orf57	3.23E-05	-21.91	9.74E-05	-7.03	1.42E-04	-5.74	1.96E-05	-2028.81
chr22.24316496-24316679>GSTTP1andDDT	GSTTP1 and DDT	4.42E-03	-3.29	7.91E-04	-7.77	9.06E-02	-1.60	4.00E-04	-17.20
chr22.44514918-44515020>PARVB	PARVB	3.20E-03	-3.27	6.91E-04	-6.35	4.81E-02	-1.74	2.99E-04	-13.44
chr5.156821041-156822687>ADAM19	ADAM19	3.01E-04	-16.01	2.15E-02	-2.06	1.13E-03	-5.14	2.42E-03	-3.71
chr6.146285293-146285525>SHPRH	SHPRH	8.44E-04	-4.84	3.55E-04	-8.05	4.01E-02	-1.75	2.61E-04	-10.55
chr6.146285293-146285559>SHPRH	SHPRH	8.44E-04	-4.84	3.55E-04	-8.05	4.01E-02	-1.75	2.61E-04	-10.55
chr6.163984476-163984751>QKI	QKI	3.99E-07	-5.23	6.22E-08	-15.32	7.43E-07	-4.34	2.09E-07	-6.72

chr6.168370462-168372588>MLLT4	MLLT4	7.37E-03	-2.24	9.05E-04	-3.79	2.40E-03	-2.86	4.22E-04	-5.10
chr6.30610545-30612432>C6orf134	C6orf134	3.16E-04	-5.69	1.56E-04	-8.92	5.98E-04	-4.30	1.39E-02	-1.97
chr7.99674926-99675056>ZNF3	ZNF3	6.70E-04	-6.08	4.74E-04	-7.65	9.91E-03	-2.37	2.97E-04	-11.79
chr8.10340434-10340741>LOC346702	LOC346702	5.43E-05	-6.06	1.95E-05	-12.38	7.60E-06	<-500	1.33E-05	-20.57
chr9.46687439-46688197>KGFLP1	KGFLP1	1.22E-05	<-500	1.22E-05	<-500	1.22E-05	<-500	3.46E-05	-10.97
chrX.40495835-40495964>CXorf38	CXorf38	1.50E-03	-7.97	4.89E-04	Not Estimable	5.85E-04	-49.12	5.41E-04	-86.98
**Upregulated in ICH**									
**Marker ID**	**Gene Symbol**	**CE Stroke vs. ICH**		**Controls vs. ICH**		**ICH vs. LV**		**ICH vs. Lacunar**	
		**p-value**	**FC**	**p-value**	**FC**	**p-value**	**FC**	**p-value**	**FC**
chr1.10509776-10510379>APITD1andCORT	APITD1 and CORT	7.04E-04	-4.22	4.26E-04	-5.20	1.17E-04	13.61	8.89E-05	20.93
chr1.112991564-112991794>CTTNBP2NL	CTTNBP2NL	2.49E-02	-1.96	7.67E-03	-2.53	4.28E-04	8.64	3.22E-03	3.21

chr1.114499947-114500540>wawleybo	wawleybo	1.41E-01	-1.44	1.46E-03	-4.21	3.22E-04	11.02	8.68E-04	5.33
chr1.145509166-145509612>RBM8A.1	RBM8A.1	1.52E-03	-4.05	2.57E-03	-3.37	4.91E-04	7.24	3.48E-04	9.57
chr1.145790974-145791170>GPR89A	GPR89A	2.08E-04	-9.88	1.17E-02	-2.13	3.99E-02	1.71	1.49E-03	3.53
chr1.150778337-150778492>CTSK	CTSK	6.96E-06	-2.75	1.17E-06	-3.85	5.52E-03	1.44	1.17E-04	1.96
chr1.150939858-150940190>LASS2	LASS2	3.59E-05	-8.01	6.54E-05	-5.76	9.12E-04	2.65	1.47E-04	4.20
chr1.154928545-154928780>SHC1andPYGO2andPBXIP1	SHC1 and PYGO2 and PBXIP1	3.65E-04	Not Estimable	1.55E-03	-6.39	4.42E-03	3.72	1.44E-02	2.53
chr1.161196029-161196394>TOMM40L	TOMM40L	1.18E-02	-2.53	9.42E-04	-7.43	2.63E-04	>500	6.66E-04	10.15
chr1.168262382-168262516>SFT2D2andTBX19	SFT2D2 and TBX19	1.10E-03	-3.16	5.90E-03	-2.19	4.08E-04	4.29	1.13E-04	8.20

chr1.17056-17742>WASH7P	WASH7P	7.18E-04	-10.55	1.11E-03	-7.10	4.84E-04	18.86	2.00E-03	4.94
chr1.180049625-180049796>CEP350	CEP350	1.98E-02	-1.72	9.32E-04	-2.93	2.29E-04	4.37	2.51E-03	2.39
chr1.180049652-180049796>CEP350	CEP350	1.98E-02	-1.72	9.32E-04	-2.93	2.29E-04	4.37	2.51E-03	2.39
chr1.19470474-19470585>UBR4	UBR4	4.12E-04	-2.86	8.91E-03	-1.76	2.07E-05	8.19	1.23E-04	3.84
chr1.201780731-201780885>NAV1	NAV1	6.28E-05	-6.82	1.22E-04	-4.98	4.14E-05	8.94	1.12E-01	1.36
chr1.235956803-235956912>LYST	LYST	4.09E-04	-7.06	9.57E-05	Not Estimable	1.31E-01	1.44	1.86E-03	3.52
chr1.243419358-243419542>SDCCAG8	SDCCAG8	3.06E-04	-87.36	2.16E-03	-4.61	1.69E-02	2.32	7.16E-03	2.94
chr1.243652316-243652442>SDCCAG8	SDCCAG8	1.83E-03	-2.98	1.58E-04	-8.34	6.05E-03	2.28	6.33E-04	4.10
chr1.26799700-26800018>HMGN2	HMGN2	1.04E-03	-2.73	3.52E-05	-10.65	7.29E-05	6.45	1.74E-04	4.42

chr1.27431807-27432578>SLC9A1	SLC9A1	4.90E-02	-1.58	3.25E-04	-4.86	1.19E-02	1.96	3.45E-03	2.47
chr1.45987501-45987609>PRDX1	PRDX1	3.62E-06	<-500	1.49E-05	-8.59	9.31E-05	3.89	4.92E-05	4.78
chr1.46467098-46468407>MAST2	MAST2	3.48E-04	-3.59	1.16E-04	-5.26	5.31E-05	8.00	1.14E-02	1.83
chr1.46805848-46806591>NSUN4andFAAH	NSUN4 and FAAH	6.05E-05	<-500	1.71E-04	-10.12	1.51E-02	1.99	6.05E-05	>500
chr1.63269390-63269533>ATG4C	ATG4C	3.01E-05	-7.06	1.05E-05	-18.05	6.94E-05	4.82	1.23E-05	14.55
chr1.78207302-78207433>USP33	USP33	1.91E-03	-3.35	4.75E-03	-2.62	1.69E-02	2.01	4.67E-04	5.92
chr1.85039599-85040103>CTBSandGNG5	CTBS and GNG5	1.53E-03	-6.62	2.47E-03	-4.94	4.75E-04	42.06	5.65E-04	23.30
chr1.85127881-85128058>SSX2IP	SSX2IP	1.86E-01	-1.31	6.50E-04	-3.62	1.03E-02	1.98	1.57E-04	6.44
chr1.89271574-89271700>PKN2	PKN2	6.12E-04	-6.28	1.13E-02	-2.29	1.87E-04	23.55	1.20E-03	4.46

chr10.11272033-11272456>CELF2	CELF2	6.92E-04	-2.68	1.36E-04	-3.96	1.47E-05	13.37	6.22E-05	5.22
chr10.32324818-32324922>KIF5B	KIF5B	2.07E-03	-2.30	1.93E-01	-1.26	2.91E-04	3.49	8.95E-03	1.84
chr10.51592090-51592619>LOC100287554	LOC100287554	9.51E-03	-2.27	4.56E-04	-6.21	1.87E-03	3.42	9.11E-04	4.43
chr10.69828759-69829524>HERC4	HERC4	9.83E-04	-5.89	1.21E-02	-2.38	5.78E-04	8.60	1.86E-04	>500
chr10.75230828-75230967>PPP3CB	PPP3CB	8.45E-04	-4.23	4.34E-03	-2.61	2.62E-04	7.78	4.88E-02	1.65
chr10.92500578-92502285>HTR7	HTR7	8.97E-05	-3.59	5.98E-02	-1.38	3.65E-04	2.65	4.87E-03	1.82
chr10.99195666-99196308>EXOSC1	EXOSC1	2.77E-04	-36.28	1.45E-02	-2.32	5.19E-04	10.77	2.56E-03	3.92
chr10.99433338-99433902>DHDPSLandPI4K2A	DHDPSL and PI4K2A	9.07E-05	-29.65	7.99E-05	-46.47	2.70E-04	7.33	1.53E-04	11.94
chr11.111889680-111893310>DIXDC1	DIXDC1	1.93E-04	-8.50	6.36E-03	-2.33	1.87E-02	1.90	3.94E-04	5.46

chr11.111889680-111893374>DIXDC1	DIXDC1	1.93E-04	-8.50	6.36E-03	-2.33	1.87E-02	1.90	3.94E-04	5.46
chr11.125490667-125490901>STT3AandCHEK1	STT3A and CHEK1	6.31E-08	<-500	7.17E-08	-95.82	6.83E-08	148.17	1.54E-07	14.95
chr11.47738539-47739064>FNBP4	FNBP4	3.24E-04	-10.00	2.29E-03	-3.47	2.00E-04	19.17	1.66E-04	29.34
chr11.61129205-61129720>CYBASC3	CYBASC3	2.94E-04	-16.63	2.29E-04	-28.81	7.91E-04	6.27	1.34E-02	2.28
chr11.62105383-62105784>saroro	saroro	2.68E-04	<-500	2.68E-04	<-500	2.68E-04	>500	2.68E-04	>500
chr11.6523983-6524156>FXC1andDNHD1	FXC1 and DNHD1	2.16E-04	-20.50	2.28E-03	-3.58	7.96E-04	5.63	1.59E-02	2.15
chr11.7479027-7479174>veemee	veemee	5.89E-05	<-500	1.17E-03	-3.62	3.17E-03	2.75	3.03E-04	6.49
chr11.836251-836525>CD151	CD151	4.18E-05	-7.43	6.65E-05	-5.80	1.16E-05	38.09	1.80E-05	15.64
chr11.89933252-89935719>CHORDC1	CHORDC1	8.39E-05	-12.95	3.53E-04	-4.87	4.83E-03	2.33	1.37E-04	8.21

chr12.10561988-10562183>KLRC4andKLRK1	KLRC4 and KLRK1	2.87E-03	-3.91	8.17E-03	-2.76	1.16E-03	6.12	3.22E-04	33.90
chr12.40441853-40442012>SLC2A13	SLC2A13	6.39E-05	-5.50	3.03E-05	-8.23	7.21E-06	>500	1.36E-05	18.27
chr12.48094974-48095387>RPAP3	RPAP3	6.56E-03	-2.64	7.99E-02	-1.59	2.33E-03	3.59	4.90E-04	7.84
chr12.54789679-54790160>ITGA5	ITGA5	9.53E-03	-2.85	1.58E-03	-6.27	5.00E-04	28.60	5.65E-04	20.66
chr12.58345541-58345678>XRCC6BP1	XRCC6BP1	4.42E-05	-135.95	1.80E-04	-7.31	1.49E-03	3.11	8.16E-05	15.47
chr12.6761437-6761584>ING4	ING4	1.90E-05	-4.46	2.22E-04	-2.56	1.61E-03	1.94	4.32E-05	3.55
chr12.96258857-96259166>SNRPF	SNRPF	6.80E-06	-15.03	3.06E-06	<-500	1.16E-05	9.15	3.05E-06	Not Estimable
chr13.100543572-100543866>CLYBL	CLYBL	1.02E-02	-2.73	8.81E-04	-9.28	3.93E-04	47.46	7.38E-04	11.24
chr13.113864293-113864812>PCID2	PCID2	4.40E-04	-10.60	6.02E-04	-7.93	1.27E-03	4.99	1.01E-02	2.47

chr13.41593364-41593568>ELF1	ELF1	4.35E-04	<-500	9.67E-04	-11.29	4.35E-04	>500	2.23E-03	5.56
chr14.100743755-100744113>YY1	YY1	4.40E-03	-3.15	1.05E-02	-2.47	2.73E-04	26.26	3.87E-04	13.52
chr14.105236090-105236707>AKT1	AKT1	4.84E-05	-8.22	2.03E-04	-4.18	2.23E-05	18.04	8.26E-05	6.02
chr14.20872770-20872931>TEP1	TEP1	1.47E-03	-2.28	8.24E-05	-4.38	3.09E-04	3.06	1.40E-02	1.67
chr14.50246313-50246524>KLHDC2	KLHDC2	3.77E-02	-1.71	4.58E-04	-5.34	6.75E-03	2.34	1.83E-03	3.20
chr14.52957557-52957723>TXNDC16	TXNDC16	1.34E-04	-4.79	3.06E-03	-2.21	3.24E-04	3.59	7.61E-04	2.90
chr14.76107075-76107403>FLVCR2andTTLL5andC14orf179	FLVCR2 and TTLL5 and C14orf179	1.92E-03	-2.58	6.71E-05	-9.03	1.22E-01	1.36	6.13E-04	3.38
chr14.88431849-88431973>GALC	GALC	5.67E-04	-3.67	2.77E-03	-2.48	1.18E-03	3.01	1.19E-04	7.15
chr14.88452833-88452946>GALC	GALC	3.97E-04	-3.47	2.38E-02	-1.65	8.56E-05	6.09	1.30E-03	2.63

chr14.96997812-96999040>PAPOLA	PAPOLA	1.96E-03	-2.78	6.03E-04	-3.85	3.36E-04	4.77	8.69E-02	1.46
chr15.30711214-30711348>rukaru	rukaru	3.01E-04	-11.05	9.53E-04	-4.94	2.39E-03	3.45	5.73E-03	2.68
chr15.38619054-38620016>koyzawbu	koyzawbu	2.03E-03	-2.87	5.39E-02	-1.58	2.95E-04	5.53	9.82E-04	3.50
chr15.57545460-57545666>stoyguby	stoyguby	3.39E-05	-5.43	8.44E-04	-2.40	9.44E-05	3.83	3.07E-03	1.98
chr15.59102429-59102587>FAM63B	FAM63B	3.05E-03	-3.01	8.90E-04	-4.60	2.53E-04	10.19	5.47E-04	5.82
chr15.59943710-59944525>GTF2A2	GTF2A2	2.05E-02	-2.18	4.44E-04	-15.25	9.30E-04	7.02	8.26E-03	2.74
chr15.64017491-64017712>HERC1	HERC1	3.68E-04	-3.52	1.84E-04	-4.37	1.36E-03	2.59	6.20E-03	1.99
chr15.66811217-66811416>ZWILCH	ZWILCH	1.42E-06	-15.75	4.47E-06	-6.69	1.61E-05	4.16	8.06E-05	2.88
chr15.66811217-66811467>ZWILCH	ZWILCH	1.42E-06	-15.75	4.47E-06	-6.69	1.61E-05	4.16	8.06E-05	2.88
chr15.75165540-75165688>SCAMP2	SCAMP2	4.19E-04	-4.48	6.10E-03	-2.22	1.95E-03	2.82	5.15E-02	1.57

chr15.80191177-80191467>ST20andMTHFS	ST20 and MTHFS	7.04E-04	-5.57	2.00E-03	-3.59	2.33E-04	13.85	3.18E-04	9.74
chr15.94774950-94775234>MCTP2	MCTP2	4.31E-04	Not Estimable	9.26E-04	-11.80	1.11E-03	9.54	4.31E-04	Not Estimable
chr16.18799866-18800440>ARL6IP1andRPS15A	ARL6IP1 and RPS15A	2.45E-04	-6.56	2.34E-03	-2.85	1.10E-02	2.06	8.95E-04	3.74
chr16.22277711-22277845>EEF2K	EEF2K	3.16E-05	-114.30	1.08E-04	-8.32	2.29E-02	1.75	3.03E-05	207.60
chr16.30593851-30595166>syrar	syrar	4.87E-05	<-500	6.03E-04	-4.33	4.87E-05	>500	4.87E-05	>500
chr16.3493611-3493837>ZNF174andNAT15andCLUAP1	ZNF174 and NAT15 and CLUAP1	3.54E-04	<-500	5.17E-04	-24.09	3.54E-04	>500	3.54E-04	>500
chr17.18087711-18088067>jeeroy	jeeroy	1.76E-04	-13.21	6.59E-04	-4.99	1.02E-04	43.74	1.19E-04	26.41
chr17.18486655-18486837>CCDC144B	CCDC144B	1.37E-02	-2.53	3.39E-04	<-500	3.82E-04	76.21	1.16E-03	7.53
chr17.27581220-27581513>CRYBA1	CRYBA1	1.03E-04	-21.86	1.74E-03	-3.27	1.66E-04	11.01	2.63E-04	7.47

chr17.36351796-36351996>TBC1D3	TBC1D3	3.07E-04	-28.30	5.13E-03	-3.10	4.66E-04	12.72	6.73E-04	8.57
chr17.40280569-40280818>RAB5C	RAB5C	2.04E-01	-1.25	8.87E-05	-4.79	2.28E-02	1.61	4.01E-03	2.03
chr17.57728564-57728677>CLTC	CLTC	3.52E-03	-2.77	3.21E-04	-7.06	8.42E-04	4.33	1.79E-03	3.34
chr17.61473104-61473289>TANC2	TANC2	3.52E-03	-2.04	7.45E-04	-2.65	4.20E-05	6.30	1.10E-05	20.53
chr17.62745780-62746126>LOC146880	LOC146880	3.51E-03	-2.39	1.24E-03	-3.00	4.23E-04	4.10	2.41E-04	5.09
chr17.77079383-77079672>ENGASE	ENGASE	3.74E-04	Not Estimable	2.87E-03	-4.54	2.80E-02	2.14	5.58E-03	3.43
chr18.48443613-48443878>ME2	ME2	1.24E-01	-1.52	1.43E-03	-5.39	4.98E-03	3.20	4.15E-04	17.42
chr18.54318248-54318824>TXNL1	TXNL1	3.27E-04	-6.40	1.59E-04	-11.19	1.61E-03	3.33	1.10E-04	18.33
chr18.67508480-67516323>DOK6	DOK6	2.50E-06	-3.44	3.70E-07	-5.86	1.18E-04	2.00	1.02E-06	4.24
chr19.11411543-11411912>tojaw	tojaw	1.12E-03	-5.75	3.37E-04	-19.59	4.08E-04	14.07	2.24E-03	4.10

chr19.13009896-13010199>SYCE2	SYCE2	5.66E-02	-1.66	1.16E-04	-220.29	3.22E-04	9.45	1.70E-04	23.14
chr19.1877203-1877424>FAM108A1	FAM108A1	9.46E-06	-12.34	3.12E-05	-5.70	4.87E-06	38.02	5.84E-06	24.14
chr19.1953385-1953505>C19orf34	C19orf34	4.17E-03	-3.77	1.47E-03	-6.50	3.53E-04	Not Estimable	9.95E-04	8.89
chr19.36515246-36515534>CLIP3	CLIP3	5.36E-02	-1.78	2.47E-04	<-500	2.47E-04	>500	2.48E-04	>500
chr19.44128266-44128394>CADM4	CADM4	1.35E-01	-1.38	7.22E-04	-3.71	2.08E-02	1.80	2.51E-04	5.61
chr19.44619641-44619995>ZNF225	ZNF225	1.90E-04	-12.35	3.11E-04	-7.83	1.23E-02	2.14	4.26E-04	6.35
chr19.47646729-47646862>SAE1	SAE1	3.77E-03	-2.25	4.22E-04	-3.71	1.10E-03	2.88	8.17E-02	1.43
chr19.47646751-47646862>SAE1	SAE1	3.77E-03	-2.25	4.22E-04	-3.71	1.10E-03	2.88	8.17E-02	1.43
chr19.49314066-49314178>BCAT2	BCAT2	4.79E-03	-3.77	3.15E-03	-4.55	1.48E-03	7.25	4.22E-04	Not Estimable
chr19.5208248-5208402>PTPRS	PTPRS	2.10E-03	-4.21	4.27E-04	-13.18	8.85E-04	6.65	5.17E-03	3.05

chr19.52207575-52207733>NCRNA00085	NCRNA00085	6.15E-05	-5.27	1.16E-05	-18.46	8.15E-06	41.81	1.56E-05	12.72
chr19.54610118-54610266>NDUFA3	NDUFA3	1.16E-04	-6.91	9.25E-03	-1.98	3.81E-05	21.56	4.72E-04	3.80
chr19.58423428-58423554>ZNF417andZNF814	ZNF417 and ZNF814	2.95E-03	-3.70	3.90E-04	-15.56	2.11E-04	>500	1.83E-02	2.20
chr19.58423428-58423557>ZNF417andZNF814	ZNF417 and ZNF814	2.95E-03	-3.70	3.90E-04	-15.56	2.11E-04	Not Estimable	1.83E-02	2.20
chr19.8441789-8441951>lyta	lyta	5.40E-04	-8.82	3.08E-04	-17.86	1.76E-03	4.33	3.90E-03	3.23
chr19.9720432-9722012>ZNF562andZNF561	ZNF562 and ZNF561	6.18E-04	-29.45	4.65E-04	-469.78	4.39E-03	4.01	5.13E-04	75.31
chr2.110584278-110584424>RGPD5	RGPD5	5.04E-05	-11.19	9.25E-04	-3.01	3.59E-04	3.92	1.20E-02	1.87
chr2.111302237-111302383>RGPD6	RGPD6	5.04E-05	-11.19	9.25E-04	-3.01	3.59E-04	3.92	1.20E-02	1.87
chr2.113175261-113175491>RGPD8	RGPD8	7.54E-05	-5.13	3.91E-05	-7.04	7.22E-06	>500	1.04E-05	31.99

chr2.118864235-118864479>INSIG2	INSIG2	5.60E-02	-1.54	1.23E-04	-7.34	2.27E-03	2.62	9.07E-04	3.27
chr2.172848099-172848599>HAT1	HAT1	1.42E-04	-4.52	4.48E-05	-7.86	8.43E-05	5.58	1.49E-05	29.53
chr2.17953901-17954051>GEN1	GEN1	9.89E-05	-25.31	6.70E-05	<-500	6.92E-05	199.20	1.58E-03	3.38
chr2.182339687-182340015>ITGA4	ITGA4	1.90E-05	-7.51	4.08E-03	-1.92	1.03E-04	3.82	1.08E-05	11.34
chr2.198175302-198175503>ANKRD44	ANKRD44	7.11E-04	-5.58	3.26E-04	-9.66	1.03E-02	2.31	2.81E-02	1.89
chr2.208446079-208446884>FAM119A	FAM119A	3.71E-05	-9.30	3.05E-04	-3.59	4.39E-03	2.08	5.29E-05	7.28
chr2.231663444-231663879>CAB39	CAB39	6.76E-03	-2.27	1.48E-03	-3.25	3.64E-02	1.69	3.63E-04	5.44
chr2.234112772-234113219>INPP5D	INPP5D	1.03E-03	-3.44	4.25E-04	-4.68	1.22E-04	9.78	2.17E-03	2.82
chr2.242282407-242282508>SEPT2	SEPT2	4.07E-03	-2.55	1.80E-03	-3.12	1.84E-04	8.63	9.02E-04	3.85

chr2.243168539-243168819>samemo	samemo	4.94E-03	-2.98	1.72E-02	-2.18	2.23E-04	37.87	5.29E-04	8.73
chr2.73957016-73957156>TPRKB	TPRKB	2.11E-05	-8.51	8.41E-06	-23.94	1.24E-05	13.51	6.75E-06	43.24
chr2.88336462-88336570>KRCC1	KRCC1	1.75E-03	-3.55	5.15E-03	-2.62	2.74E-02	1.86	1.75E-04	15.65
chr20.18449588-18449705>POLR3F	POLR3F	4.95E-05	-10.82	1.16E-03	-2.83	2.93E-04	4.13	1.75E-05	Not Estimable
chr20.23401942-23402097>NAPB	NAPB	6.00E-05	<-500	4.50E-04	-5.29	5.97E-05	>500	1.09E-04	17.28
chr20.30720816-30720929>TM9SF4	TM9SF4	4.31E-04	-2.58	7.10E-02	-1.36	1.55E-03	2.11	8.38E-03	1.71
chr20.34487292-34487561>PHF20	PHF20	1.17E-03	-5.87	7.20E-04	-8.27	4.74E-04	12.86	2.59E-03	3.97
chr20.416929-419485>TBC1D20	TBC1D20	1.36E-03	-3.68	3.76E-04	-6.52	7.71E-04	4.55	7.72E-03	2.33
chr20.43808628-43808775>rotora	rotora	2.81E-04	-11.96	1.75E-03	-3.84	4.47E-04	7.75	3.40E-03	3.09
chr21.40619627-40619758>BRWD1	BRWD1	7.54E-05	-4.31	7.25E-06	-21.38	1.26E-05	10.81	3.76E-05	5.59

chr21.47608408-47608855>klorley	klorley	1.80E-02	-2.14	1.56E-03	-4.41	4.26E-04	10.24	6.69E-03	2.71
chr22.20093700-20093800>DGCR8	DGCR8	6.95E-03	-2.58	8.09E-04	-5.52	2.17E-04	19.30	2.11E-03	3.65
chr22.31733654-31734031>sneypoy	sneypoy	5.37E-02	-1.82	3.99E-04	-39.22	1.49E-03	6.03	1.08E-02	2.67
chr22.41175013-41175129>SLC25A17	SLC25A17	7.16E-04	-7.40	3.66E-04	-14.98	6.05E-03	2.87	9.34E-02	1.58
chr22.45254869-45255776>PRR5-ARHGAP8	PRR5-ARHGAP8	1.71E-02	-2.04	9.61E-05	<-500	3.03E-04	8.85	1.52E-03	3.77
chr22.50320903-50321181>CRELD2	CRELD2	1.98E-03	-4.87	3.50E-02	-1.97	5.27E-04	14.94	4.59E-04	19.20
chr22.51221467-51221714>RABL2B	RABL2B	4.84E-06	<-500	9.09E-06	-18.75	5.55E-06	85.86	2.67E-05	7.08
chr3.122283274-122283460>DTX3L	DTX3L	3.86E-03	-2.45	8.96E-04	-3.51	1.54E-01	1.35	4.78E-04	4.34
chr3.137963865-137964523>vusmyby	vusmyby	4.47E-04	-6.75	1.85E-02	-2.01	7.96E-04	4.93	2.47E-04	10.96

chr3.137963930-137964523>ARMC8	ARMC8	4.47E-04	-6.75	1.85E-02	-2.01	7.96E-04	4.93	2.47E-04	10.96
chr3.137963930-137964524>ARMC8	ARMC8	4.47E-04	-6.75	1.85E-02	-2.01	7.96E-04	4.93	2.47E-04	10.96
chr3.150280329-150280447>EIF2A	EIF2A	1.05E-02	-1.55	4.23E-05	-3.25	1.90E-04	2.48	1.23E-03	1.93
chr3.15778540-15778740>ANKRD28	ANKRD28	1.71E-04	-3.90	1.21E-05	-23.79	1.60E-03	2.36	2.11E-05	11.26
chr3.167452594-167452717>PDCD10	PDCD10	4.97E-03	-3.83	4.68E-04	Not Estimable	2.62E-03	5.24	1.60E-03	7.32
chr3.20019802-20020396>RAB5A	RAB5A	4.94E-02	-1.54	3.15E-04	-4.25	8.07E-04	3.20	1.83E-03	2.64
chr3.23929058-23929280>UBE2E1	UBE2E1	1.32E-03	-3.40	3.40E-04	-5.78	6.07E-02	1.57	2.24E-04	7.41
chr3.25637911-25639423>RARB	RARB	6.84E-03	-2.86	4.19E-02	-1.86	4.93E-04	12.19	2.09E-03	4.35
chr3.39162488-39162680>TTC21A	TTC21A	2.31E-02	-1.64	3.37E-04	-3.48	5.93E-05	6.73	3.03E-05	10.81
chr3.52385978-52386119>DNAH1	DNAH1	2.86E-05	-25.66	1.71E-04	-5.17	1.52E-02	1.80	3.60E-04	3.92

chr3.52561845-52561947>NT5DC2	NT5DC2	5.07E-05	Not Estimable	5.07E-05	<-500	6.23E-05	50.64	5.07E-05	Not Estimable
chr3.69028819-69028938>C3orf64	C3orf64	4.34E-04	-276.88	1.44E-03	-7.40	6.00E-04	25.32	9.40E-04	11.26
chr3.81552424-81552865>chordybo	chordybo	2.18E-04	-6.44	4.03E-05	Not Estimable	4.30E-04	4.65	7.72E-03	2.16
chr4.122723829-122723948>EXOSC9	EXOSC9	1.45E-03	-2.35	3.00E-04	-3.21	1.36E-02	1.70	9.05E-05	4.55
chr4.122723829-122723983>EXOSC9	EXOSC9	1.45E-03	-2.35	3.00E-04	-3.21	1.36E-02	1.70	9.05E-05	4.55
chr4.157731989-157732169>PDGFC	PDGFC	7.75E-04	-2.40	6.25E-03	-1.79	4.00E-05	4.91	9.27E-05	3.76
chr4.175223190-175223337>KIAA1712	KIAA1712	3.59E-04	-10.22	9.07E-02	-1.55	6.69E-03	2.62	1.66E-03	4.04
chr4.40800804-40800921>NSUN7	NSUN7	6.85E-02	-1.65	4.03E-04	-10.82	2.93E-04	16.64	1.48E-03	4.51
chr4.76874494-76874938>sporsmorby	sporsmorby	8.09E-03	-2.96	4.72E-04	-28.20	2.56E-02	2.16	2.57E-01	1.34

chr5.134343647-134343829>PCBD2andCATSPER3	PCBD2 and CATSPER3	1.80E-02	-2.37	6.68E-03	-3.17	3.53E-04	Not Estimable	5.92E-04	17.76
chr5.139929370-139930496>APBB3andSRA1	APBB3 and SRA1	2.46E-01	-1.21	2.97E-04	-3.06	1.24E-02	1.69	1.95E-03	2.17
chr5.140895496-140896575>DIAPH1	DIAPH1	2.42E-03	-3.84	3.56E-04	-14.80	2.77E-04	23.85	9.58E-04	5.96
chr5.140895875-140896575>DIAPH1	DIAPH1	5.18E-05	-5.97	9.76E-06	-34.42	1.45E-05	16.01	1.20E-05	21.48
chr5.14381239-14381361>TRIO	TRIO	7.18E-04	-3.91	1.44E-02	-1.95	2.22E-04	6.59	1.32E-03	3.25
chr5.145493406-145493874>LARS	LARS	4.03E-04	-11.34	3.32E-03	-3.36	8.40E-04	6.17	5.02E-04	9.06
chr5.35053745-35054334>fugey	fugey	4.00E-04	-9.01	8.14E-04	-5.54	3.29E-04	10.88	1.32E-04	Not Estimable
chr5.39274505-39274630>FYB	FYB	1.63E-02	-2.33	3.89E-04	-24.35	1.29E-03	6.00	4.56E-02	1.85
chr5.70531277-70532281>goychyby	goychyby	1.10E-02	-2.03	2.29E-04	-6.29	6.59E-04	3.97	2.19E-03	2.81

chr5.77656415-77656552>SCAMP1	SCAMP1	1.07E-03	-4.46	2.97E-04	-9.88	4.46E-04	7.11	3.67E-02	1.79
chr6.109248281-109249436>ARMC2	ARMC2	2.72E-03	-2.24	1.30E-02	-1.77	2.08E-04	4.04	8.43E-02	1.40
chr6.122792844-122793050>SERINC1	SERINC1	1.45E-02	-2.14	5.74E-03	-2.63	2.50E-03	3.31	3.03E-04	9.93
chr6.144289727-144290115>PLAGL1andHYMAI	PLAGL1 and HYMAI	1.74E-01	-1.44	4.00E-04	-32.52	4.43E-04	23.92	4.81E-03	3.42
chr6.153291654-153292549>FBXO5	FBXO5	3.31E-04	-3.25	1.32E-03	-2.43	4.35E-03	2.01	2.30E-02	1.61
chr6.153291660-153292549>FBXO5	FBXO5	3.31E-04	-3.25	1.32E-03	-2.43	4.35E-03	2.01	2.30E-02	1.61
chr6.153291674-153292549>FBXO5	FBXO5	3.31E-04	-3.25	1.32E-03	-2.43	4.35E-03	2.01	2.30E-02	1.61
chr6.158088239-158089557>fyjaw	fyjaw	8.63E-04	-4.25	2.52E-02	-1.85	1.77E-03	3.33	2.99E-04	7.26
chr6.3021094-3022352>teyvybo	teyvybo	3.80E-05	-140.84	4.06E-05	-74.72	9.11E-04	3.45	1.27E-04	8.48

chr6.34360041-34360260>RPS10andNUDT3	RPS10 and NUDT3	5.81E-04	-4.24	8.57E-05	-15.64	7.24E-05	20.73	1.60E-04	8.25
chr6.41036580-41036692>C6orf130andUNC5CL	C6orf130 and UNC5CL	3.57E-04	-5.13	1.22E-04	-10.16	7.37E-04	3.87	1.49E-03	3.13
chr6.41751200-41751976>PRICKLE4andTOMM6	PRICKLE4 and TOMM6	4.62E-03	-2.80	2.51E-04	-12.58	4.03E-04	7.96	9.63E-04	4.78
chr6.79664949-79665569>PHIPandTRNAF13P	PHIP and TRNAF13P	1.35E-03	-2.92	2.55E-05	Not Estimable	1.30E-04	6.84	5.14E-05	15.62
chr7.149598-152547>kehera	kehera	1.33E-02	-1.71	1.29E-03	-2.42	9.72E-02	1.36	4.40E-04	2.99
chr7.22980878-22987334>FAM126A	FAM126A	5.13E-04	-2.56	4.24E-05	-4.72	8.77E-03	1.71	1.84E-03	2.09
chr7.2635311-2636062>dochuby	dochuby	3.67E-04	-4.19	1.69E-04	-5.80	4.01E-03	2.30	2.63E-02	1.70
chr7.29549802-29552165>klerky	klerky	1.88E-04	-6.48	3.73E-03	-2.44	2.29E-02	1.77	8.77E-02	1.46

chr7.45083306-45083697>CCM2	CCM2	2.52E-02	-1.90	8.39E-04	-4.83	1.36E-03	3.96	3.71E-04	7.72
chr7.5938415-5938550>CCZ1	CCZ1	5.04E-07	-252.68	2.41E-06	-8.08	7.48E-06	4.89	1.37E-06	12.27
chr7.74166365-74166897>GTF2I	GTF2I	2.27E-03	-3.76	5.26E-04	-8.20	7.50E-04	6.36	2.45E-04	22.31
chr7.76870183-76870364>CCDC146	CCDC146	1.69E-05	-12.37	4.46E-05	-6.24	4.43E-04	2.97	6.52E-06	Not Estimable
chr8.104455023-104455428>DCAF13	DCAF13	4.47E-02	-1.81	1.12E-02	-2.44	5.04E-03	3.04	4.85E-04	10.67
chr8.133984843-133984986>TG	TG	3.27E-02	-1.99	2.69E-04	<-500	2.69E-04	Not Estimable	2.69E-04	>500
chr8.24256387-24256553>ADAMDEC1	ADAMDEC1	1.89E-02	-2.37	1.55E-03	-6.56	3.81E-04	Not Estimable	5.79E-02	1.82
chr8.30948350-30948458>WRN	WRN	9.84E-05	-8.13	4.33E-05	-19.99	6.54E-04	3.52	1.58E-04	6.10
chr8.62438536-62438671>ASPH	ASPH	1.08E-03	-5.20	5.24E-03	-2.88	2.06E-02	2.07	3.63E-04	11.88
chr8.74858684-74859055>TCEB1	TCEB1	2.36E-04	-11.51	1.83E-04	-16.10	3.38E-04	8.24	1.05E-03	4.38

chr9.17135038-17135423>CNTLN	CNTLN	3.03E-02	-1.91	1.90E-03	-3.97	8.64E-04	5.73	4.80E-04	8.60
chr9.33264164-33264493>CHMP5	CHMP5	1.44E-04	-11.82	8.33E-04	-4.08	1.94E-04	8.91	4.09E-04	5.53
chr9.35737655-35737936>GBA2	GBA2	6.33E-03	-3.43	4.89E-04	-96.76	1.37E-03	8.06	5.25E-04	55.35
chrX.118985730-118985836>UPF3B	UPF3B	1.13E-04	<-500	1.55E-03	-3.93	3.89E-03	2.93	8.72E-04	4.99
chrX.138864706-138864887>ATP11C	ATP11C	3.68E-03	-3.31	2.76E-04	-23.71	1.59E-02	2.23	9.95E-04	5.80
chrX.149924161-149924396>MTMR1	MTMR1	1.84E-03	-6.49	4.58E-04	<-500	2.92E-03	4.89	6.99E-03	3.33
chrX.153744234-153744566>FAM3A	FAM3A	1.07E-04	-54.24	3.85E-04	-7.03	2.63E-04	9.43	8.88E-04	4.53
chrX.15862547-15863639>AP1S2	AP1S2	9.09E-05	-5.62	1.65E-04	-4.42	4.01E-03	2.12	6.08E-02	1.46
chrX.16870674-16871149>RBBP7	RBBP7	6.02E-03	-2.99	2.65E-03	-3.99	8.60E-04	7.37	3.25E-04	29.04
chrX.2839944-2840065>ARSD	ARSD	1.44E-03	-3.53	4.55E-02	-1.67	3.00E-03	2.86	3.95E-04	6.06

chrX.74282163-74282417>ABCB7	ABCB7	1.09E-03	-5.81	8.03E-04	-7.10	1.32E-02	2.36	3.38E-04	19.17
chrX.76776266-76776394>ATRX	ATRX	3.40E-06	-5.28	1.40E-04	-2.35	1.89E-03	1.75	6.84E-06	4.23
chrX.77303661-77305892>ATP7A	ATP7A	1.55E-04	-10.33	2.03E-03	-3.04	3.29E-01	1.22	1.05E-03	3.70

Upregulated in Controls									
**Marker ID**	**Gene Symbol**	**CE Stroke vs. Controls**		**Controls vs. ICH**		**Controls vs. LV**		**Controls vs. Lacunar**	
		**p-value**	**FC**	**p-value**	**FC**	**p-value**	**FC**	**p-value**	**FC**
chr1.53416427-53416558>SCP2	SCP2	4.14E-04	-3.65	3.66E-02	1.59	1.42E-03	2.69	6.07E-03	2.06
chr14.19683027-19683434>DUXAP10	DUXAP10	3.62E-05	-29.94	1.14E-04	7.36	2.52E-05	>500	4.66E-05	17.76
chr17.42982993-42984756>GFAP	GFAP	6.98E-04	-5.06	1.25E-02	2.15	4.86E-03	2.65	2.89E-04	8.76
chr18.28642978-28643439>DSC2	DSC2	2.44E-02	-1.84	5.93E-03	2.41	1.09E-03	3.79	1.88E-04	9.74
chr18.43417478-43417850>SIGLEC15	SIGLEC15	3.26E-04	<-500	2.04E-03	5.10	1.43E-02	2.49	4.84E-02	1.87
chr19.39138368-39138547>ACTN4	ACTN4	1.33E-05	-8.92	1.30E-04	3.51	5.83E-06	22.30	3.57E-05	5.30

chr19.45543176-45543569>SFRS16	SFRS16	1.43E-04	<-500	4.87E-03	2.87	3.81E-04	9.97	2.38E-04	18.83
chr2.101606718-101606908>NPAS2	NPAS2	2.57E-04	-6.61	5.03E-04	4.72	7.16E-05	30.24	3.23E-03	2.67
chr2.242611606-242612016>ATG4B	ATG4B	8.42E-05	-8.04	3.53E-05	20.98	1.89E-04	5.17	1.07E-03	2.97
chr20.32880178-32880359>AHCY	AHCY	2.22E-03	-4.21	5.50E-03	3.04	3.66E-04	18.76	1.14E-02	2.48
chr22.36892014-36892255>FOXRED2andTXN2	FOXRED2 and TXN2	2.48E-04	-5.82	1.19E-02	1.99	4.04E-03	2.43	7.88E-02	1.49
chr22.41252435-41253036>ST13	ST13	1.56E-03	-7.62	3.49E-03	4.55	4.81E-04	>500	4.81E-04	>500
chr6.32806430-32806547>TAP2andHLA-DOB	TAP2 and HLA-DOB	9.55E-05	<-500	2.92E-03	3.07	2.70E-04	9.81	7.48E-04	5.03
chr7.101475858-101476865>snorkar	snorkar	2.78E-04	-23.06	2.33E-02	2.05	2.70E-03	3.68	6.83E-03	2.75
chr9.140473077-140473340>WDR85	WDR85	3.68E-05	-31.77	4.74E-04	3.93	1.31E-04	6.90	6.73E-05	11.64

chr9.95018962-95019082>IARS	IARS	1.52E-05	-7.76	2.30E-04	3.02	7.17E-05	4.05	2.90E-05	5.60
chr9.96866557-96866667>PTPDC1	PTPDC1	3.10E-07	Not Estimable	7.27E-04	1.95	8.40E-07	13.03	3.13E-07	>500
chrX.48367956-48368344>PORCN	PORCN	4.63E-05	-86.44	4.10E-05	>500	4.91E-05	58.47	7.98E-05	16.02
