# Supplementary material for: Intracerebral Hemorrhage and Ischemic Stroke of Different Etiologies Have Distinct Alternatively Spliced mRNA Profiles in the Blood: a Pilot RNA-seq Study
Source: Transl Stroke Res. 2015 May 22;6(4):284–9. doi: 10.1007/s12975-015-0407-9 (PMC4485700; doi:10.1007/s12975-015-0407-9)
Supplement: Supplementary file 12 — Over-represented pathways and gene ontology for the 308 exons that showed differential exon usage between the five groups (Large Vessel IS, Cardioembolic IS, Lacunar IS, ICH, Controls). (PDF 65 kb) [file 12975_2015_407_MOESM8_ESM.pdf]

SUPPLEMENTARY TABLE 8. Over-represented Pathways and Gene Ontology for the Upregulated Exons

Cardioembolic Ischemic Stroke

SIGNIFICANTLY ENRICHED CANONICAL PATHWAYS OF CARDIOEMBOLIC ISCHEMIC STROKE

Ingenuity Canonical Pathways	-log(p-value)	B-H p-value	Ratio	Molecules
Semaphorin Signaling in Neurons	1.41E00	6.9E-03-7.47E-02	1.89E-02	PLXNB1

GENE ONTOLOGY OF CARDIOEMBOLIC ISCHEMIC STROKE

Annotation Cluster 1 **Enrichment Score: 1.5478345985149118**

Category	Term	Count	%	PValue	Genes	List Total	Pop Hits	Pop Total	Fold Enrichment	Benjamini	FDR
GOTERM_MF_FAT	GO:0046872~metal ion binding	9	47.37	0.03	STEAP3, ZNF33A, UPF1, LPP, ZNF417, ZNF814, TRPV5, TTN, SLC10A1	14	4140	12983	2.02	0.89	24.97
GOTERM_MF_FAT	GO:0043169~cation binding	9	47.37	0.03	STEAP3, ZNF33A, UPF1, LPP, ZNF417, ZNF814, TRPV5, TTN, SLC10A1	14	4179	12983	2.00	0.69	26.24
GOTERM_MF_FAT	GO:0043167~ion binding	9	47.37	0.03	STEAP3, ZNF33A, UPF1, LPP, ZNF417, ZNF814, TRPV5, TTN, SLC10A1	14	4241	12983	1.97	0.58	28.33

Annotation Cluster 2 **Enrichment Score: 1.4272404656779223**

Category	Term	Count	%	PValue	Genes	List Total	Pop Hits	Pop Total	Fold Enrichment	Benjamini	FDR
GOTERM_CC_FAT	GO:0005887~integral to plasma membrane	4	21.05	0.03	PLXNB1, PTPRN2, TRPV5, SLC10A1	9	1188	12782	4.78	0.75	25.81
GOTERM_CC_FAT	GO:0031226~intrinsic to plasma membrane	4	21.05	0.03	PLXNB1, PTPRN2, TRPV5, SLC10A1	9	1215	12782	4.68	0.53	27.17
GOTERM_CC_FAT	GO:0044459~plasma membrane part	5	26.32	0.03	PLXNB1, LPP, PTPRN2, TRPV5, SLC10A1	9	2203	12782	3.22	0.40	27.86
GOTERM_CC_FAT	GO:0005886~plasma membrane	6	31.58	0.05	STEAP3, PLXNB1, LPP, PTPRN2, TRPV5, SLC10A1	9	3777	12782	2.26	0.46	40.79

Annotation Cluster 3 **Enrichment Score: 1.228190007997564**

Category	Term	Count	%	PValue	Genes	List Total	Pop Hits	Pop Total	Fold Enrichment	Benjamini	FDR
GOTERM_BP_FAT	GO:0030001~metal ion transport	3	15.79	0.05	STEAP3, TRPV5, SLC10A1	12	465	13528	7.27	1.00	48.17
GOTERM_CC_FAT	GO:0005886~plasma membrane	6	31.58	0.05	STEAP3, PLXNB1, LPP, PTPRN2, TRPV5, SLC10A1	9	3777	12782	2.26	0.46	40.79
GOTERM_BP_FAT	GO:0006812~cation transport	3	15.79	0.07	STEAP3, TRPV5, SLC10A1	12	553	13528	6.12	1.00	59.48

Large Vessel Ischemic Stroke

SIGNIFICANTLY ENRICHED CANONICAL PATHWAYS OF LARGE VESSEL ISCHEMIC STROKE

Ingenuity Canonical Pathways	-log(p-value)	-log(B-H p-value)	Ratio	Molecules
Oxidized GTP and dGTP Detoxification	2.44E00	1E00	3.33E-01	NUDT1

UDP-N-acetyl-D-glucosamine Biosynthesis II	2.14E00	1E00	1.67E-01	GNPNAT1
Glycoaminoglycan-protein Linkage Region Biosynthesis	2.08E00	1E00	1.43E-01	B3GAT3
UDP-N-acetyl-D-galactosamine Biosynthesis II	1.97E00	1E00	1.11E-01	GNPNAT1
Thyroid Hormone Metabolism II (via Conjugation and/or Degradation)	1.44E00	7.92E-01	3.23E-02	B3GAT3
Nucleotide Excision Repair Pathway	1.38E00	7.92E-01	2.86E-02	ERCC5

GENE ONTOLOGY OF LARGE VESSEL ISCHEMIC STROKE

Annotation Cluster 1 **Enrichment Score: 1.9116473785151364**

Category	Term	Count	%	PValue	Genes	List Total	Pop Hits	Pop Total	Fold Enrichment	Benjamini	FDR
GOTERM_CC_FAT	GO:0070013~intracellular organelle lumen	8	26.667	0.01	PDK1, ERCC5, PNMA3, CENPF, PCSK6, DIMT1L, DNAJA3, CBX5	20	1779	12782	2.87	0.64	11.29
GOTERM_CC_FAT	GO:0043233~organelle lumen	8	26.667	0.01	PDK1, ERCC5, PNMA3, CENPF, PCSK6, DIMT1L, DNAJA3, CBX5	20	1820	12782	2.81	0.43	12.68
GOTERM_CC_FAT	GO:0031974~membrane-enclosed lumen	8	26.667	0.01	PDK1, ERCC5, PNMA3, CENPF, PCSK6, DIMT1L, DNAJA3, CBX5	20	1856	12782	2.75	0.34	13.98

Annotation Cluster 2 **Enrichment Score: 1.6656724740255078**

Category	Term	Count	%	PValue	Genes	List Total	Pop Hits	Pop Total	Fold Enrichment	Benjamini	FDR
GOTERM_BP_FAT	GO:0043066~negative regulation of apoptosis	4	13.333	0.01	ERCC5, RIPK2, PCSK6, DNAJA3	20	354	13528	7.64	1.00	16.49
GOTERM_BP_FAT	GO:0043069~negative regulation of programmed cell death	4	13.333	0.01	ERCC5, RIPK2, PCSK6, DNAJA3	20	359	13528	7.54	0.95	17.07
GOTERM_BP_FAT	GO:0060548~negative regulation of cell death	4	13.333	0.01	ERCC5, RIPK2, PCSK6, DNAJA3	20	360	13528	7.52	0.87	17.19
GOTERM_BP_FAT	GO:0042981~regulation of apoptosis	4	13.333	0.10	ERCC5, RIPK2, PCSK6, DNAJA3	20	804	13528	3.37	0.96	77.51

Annotation Cluster 3 **Enrichment Score: 1.3439751095199926**

Category	Term	Count	%	PValue	Genes	List Total	Pop Hits	Pop Total	Fold Enrichment	Benjamini	FDR
GOTERM_BP_FAT	GO:0016481~negative regulation of transcription	4	13.333	0.03	LMCD1, CENPF, NSD1, CBX5	20	459	13528	5.89	0.94	30.30
GOTERM_MF_FAT	GO:0003682~chromatin binding	3	10	0.03	CENPF, NSD1, CBX5	24	150	12983	10.82	0.98	28.76

GOTERM_BP_FAT	GO:0010629~negative regulation of gene expression	4	13.333	0.03	LMCD1, CENPF, NSD1, CBX5	20	504	13528	5.37	0.95	36.94
GOTERM_BP_FAT	GO:0045934~negative regulation of nucleobase, nucleoside, nucleotide and nucleic acid metabolic process	4	13.333	0.03	LMCD1, CENPF, NSD1, CBX5	20	512	13528	5.28	0.89	38.15
GOTERM_BP_FAT	GO:0051172~negative regulation of nitrogen compound metabolic process	4	13.333	0.03	LMCD1, CENPF, NSD1, CBX5	20	519	13528	5.21	0.86	39.20
GOTERM_BP_FAT	GO:0010558~negative regulation of macromolecule biosynthetic process	4	13.333	0.04	LMCD1, CENPF, NSD1, CBX5	20	547	13528	4.95	0.87	43.44
GOTERM_BP_FAT	GO:0031327~negative regulation of cellular biosynthetic process	4	13.333	0.04	LMCD1, CENPF, NSD1, CBX5	20	561	13528	4.82	0.86	45.56
GOTERM_BP_FAT	GO:0009890~negative regulation of biosynthetic process	4	13.333	0.04	LMCD1, CENPF, NSD1, CBX5	20	573	13528	4.72	0.84	47.38
GOTERM_MF_FAT	GO:0008134~transcription factor binding	4	13.333	0.06	LMCD1, CENPF, NSD1, DNAJA3	24	513	12983	4.22	0.98	51.71
GOTERM_BP_FAT	GO:0010605~negative regulation of macromolecule metabolic process	4	13.333	0.08	LMCD1, CENPF, NSD1, CBX5	20	734	13528	3.69	0.96	69.72
GOTERM_BP_FAT	GO:0045892~negative regulation of transcription, DNA-dependent	3	10	0.09	LMCD1, NSD1, CBX5	20	356	13528	5.70	0.96	72.98
GOTERM_BP_FAT	GO:0051253~negative regulation of RNA metabolic process	3	10	0.09	LMCD1, NSD1, CBX5	20	362	13528	5.61	0.95	74.03

Lacunar Ischemic Stroke

SIGNIFICANTLY ENRICHED CANONICAL PATHWAYS OF LACUNAR ISCHEMIC STROKE

Ingenuity Canonical Pathways	-log(p-value)	-log(B-H p-value)	Ratio	Molecules
Eumelanin Biosynthesis	2.34E00	7.03E-01	2.5E-01	DDT
G Protein Signaling Mediated by Tubby	1.43E00	6.15E-01	3.03E-02	GNG3

GENE ONTOLOGY OF LACUNAR ISCHEMIC STROKE

Not Available with 0.1 Ease

Intracerebral Hemorrhagic Stroke

SIGNIFICANTLY ENRICHED CANONICAL PATHWAYS OF INTRACEREBRAL HEMORRHAGIC STROKE

Ingenuity Canonical Pathways	-log(p-value)	-log(B-H p-value)	Ratio	Molecules
Regulation of eIF4 and p70S6K Signaling	3.54E00	1.14E00	4.79E-02	SHC1,AKT1,RPS10,ITGA5,RPS15A,EIF2A,ITGA4
PTEN Signaling	3.24E00	1.14E00	5.08E-02	MAST2,SHC1,AKT1,ITGA5,INPP5D,ITGA4
Actin Cytoskeleton Signaling	2.53E00	8.12E-01	3.23E-02	SHC1,DIAPH1,ITGA5,TRIO,SLC9A1,PDGFC,ITGA4

IL-3 Signaling	2.47E00	8.12E-01	5.63E-02	SHC1,AKT1,PPP3CB,INPP5D
Caveolar-mediated Endocytosis Signaling	2.45E00	8.12E-01	5.56E-02	RAB5A,RAB5C,ITGA5,ITGA4
Ephrin Receptor Signaling	2.38E00	8.12E-01	3.45E-02	SHC1,AKT1,ITGA5,GNG5,PDGFC,ITGA4
PI3K/AKT Signaling	2.36E00	8.12E-01	4.07E-02	SHC1,AKT1,ITGA5,INPP5D,ITGA4
FcγRIIB Signaling in B Lymphocytes	2.27E00	8.12E-01	7.32E-02	SHC1,AKT1,INPP5D
Clathrin-mediated Endocytosis Signaling	2.25E00	8.12E-01	3.24E-02	RAB5A,RAB5C,PPP3CB,CLTC,ITGA5,PDGFC
DNA Double-Strand Break Repair by Homologous Recombination	2.2E00	8.08E-01	1.43E-01	GEN1,ATRX
Neuregulin Signaling	2.14E00	8.08E-01	4.55E-02	SHC1,AKT1,ITGA5,ITGA4
PAK Signaling	2.12E00	8.08E-01	4.49E-02	SHC1,ITGA5,PDGFC,ITGA4
Telomerase Signaling	1.96E00	6.84E-01	4.04E-02	SHC1,AKT1,TEP1,ELF1
HGF Signaling	1.88E00	6.48E-01	3.81E-02	AKT1,ITGA5,ELF1,ITGA4
iCOS-iCOSL Signaling in T Helper Cells	1.83E00	6.48E-01	3.7E-02	SHC1,AKT1,PPP3CB,INPP5D
Natural Killer Cell Signaling	1.81E00	6.48E-01	3.64E-02	SHC1,AKT1,KLRC4-KLRK1/KLRK1,INPP5D
GM-CSF Signaling	1.78E00	6.48E-01	4.84E-02	SHC1,AKT1,PPP3CB
4-hydroxyproline Degradation I	1.76E00	6.48E-01	5E-01	HOGA1
Anandamide Degradation	1.76E00	6.48E-01	5E-01	FAAH
Macropinocytosis Signaling	1.67E00	5.8E-01	4.41E-02	RAB5A,ITGA5,PDGFC
EIF2 Signaling	1.64E00	5.8E-01	2.7E-02	SHC1,AKT1,RPS10,RPS15A,EIF2A
mTOR Signaling	1.61E00	5.8E-01	2.66E-02	AKT1,RPS10,RPS15A,PDGFC,ARHGAP8/PRR5-ARHGAP8
NF-κB Activation by Viruses	1.59E00	5.8E-01	4.11E-02	AKT1,ITGA5,ITGA4
Tyrosine Biosynthesis IV	1.59E00	5.8E-01	3.33E-01	PCBD2

FLT3 Signaling in Hematopoietic Progenitor Cells	1.57E00	5.8E-01	4.05E-02	SHC1,AKT1,INPP5D
IL-4 Signaling	1.55E00	5.69E-01	3.95E-02	SHC1,AKT1,INPP5D
PDGF Signaling	1.53E00	5.69E-01	3.9E-02	SHC1,PDGFC,INPP5D
Reelin Signaling in Neurons	1.5E00	5.57E-01	3.8E-02	AKT1,ITGA5,ITGA4
Phenylalanine Degradation I (Aerobic)	1.46E00	5.35E-01	2.5E-01	PCBD2
FAK Signaling	1.4E00	5E-01	3.45E-02	AKT1,ITGA5,ITGA4
CTLA4 Signaling in Cytotoxic T Lymphocytes	1.38E00	5E-01	3.41E-02	AKT1,AP1S2,CLTC
G Beta Gamma Signaling	1.38E00	5E-01	3.41E-02	SHC1,AKT1,GNG5
Virus Entry via Endocytic Pathways	1.37E00	5E-01	3.37E-02	CLTC,ITGA5,ITGA4
VEGF Signaling	1.35E00	5E-01	3.3E-02	SHC1,AKT1,PDGFC
Fcγ Receptor-mediated Phagocytosis in Macrophages and Monocytes	1.32E00	5E-01	3.23E-02	AKT1,FYB,INPP5D
PPAR Signaling	1.31E00	5E-01	3.19E-02	SHC1,SRA1,PDGFC
Tec Kinase Signaling	1.31E00	5E-01	2.53E-02	GTF2I,ITGA5,GNG5,ITGA4
Glioma Signaling	1.3E00	5E-01	3.16E-02	SHC1,AKT1,PDGFC
Huntington's Disease Signaling	1.3E00	5E-01	2.17E-02	SHC1,AKT1,CLTC,GNG5,NAPB

GENE ONTOLOGY OF INTRACEREBRAL HEMORRHAGIC STROKE

Annotation Cluster 1 **Enrichment Score: 2.0008035747785273**

Category	Term	Count	%	PValue	Genes	List Total	Pop Hits	Pop Total	Fold Enrichment	Benjamini	FDR
GOTERM_BP_FAT	GO:0015031~protein transport	19	10.053	6.74E-04	RGPD6, SCAMP1, ARL6IP1, FYB, RGPD5, RGPD8, SCAMP2, RAB5C, CHMP5, NAPB, CLTC, AKT1, SFT2D2, AP1S2, TOMM6, ATG4C, LYST, FXC1, TOMM40L, RAB5A, PPP3CB	138	762	13528	2.44	0.57	1.09

GOTERM_BP_FAT	GO:0045184~establishment of protein localization	19	10.053	7.50E-04	RGPD6, SCAMP1, ARL6IP1, FYB, RGPD5, RGPD8, SCAMP2, RAB5C, CHMP5, NAPB, CLTC, AKT1, SFT2D2, AP1S2, TOMM6, ATG4C, LYST, FXC1, TOMM40L, RAB5A, PPP3CB	138	769	13528	2.42	0.37	1.21
GOTERM_BP_FAT	GO:0008104~protein localization	19	10.053	3.43E-03	RGPD6, SCAMP1, ARL6IP1, FYB, RGPD5, RGPD8, SCAMP2, RAB5C, CHMP5, NAPB, CLTC, AKT1, SFT2D2, AP1S2, TOMM6, ATG4C, LYST, FXC1, TOMM40L, RAB5A, PPP3CB	138	882	13528	2.11	0.76	5.43
GOTERM_BP_FAT	GO:0046907~intracellular transport	15	7.9365	6.75E-03	RGPD6, ARL6IP1, FYB, SCAMP1, RGPD5, RGPD8, SCAMP2, KIF5B, CHMP5, NAPB, CLTC, AKT1, AP1S2, ATG4C, LYST, FXC1, PPP3CB	138	657	13528	2.24	0.88	10.42
GOTERM_BP_FAT	GO:0006886~intracellular protein transport	9	4.7619	3.64E-02	FYB, ARL6IP1, AKT1, AP1S2, ATG4C, FXC1, PPP3CB, NAPB, CLTC	138	374	13528	2.36	1.00	45.30
GOTERM_BP_FAT	GO:0034613~cellular protein localization	9	4.7619	5.75E-02	FYB, ARL6IP1, AKT1, AP1S2, ATG4C, FXC1, PPP3CB, NAPB, CLTC	138	411	13528	2.15	1.00	61.82
GOTERM_BP_FAT	GO:0070727~cellular macromolecule localization	9	4.7619	5.95E-02	FYB, ARL6IP1, AKT1, AP1S2, ATG4C, FXC1, PPP3CB, NAPB, CLTC	138	414	13528	2.13	1.00	63.10
GOTERM_BP_FAT	GO:0006605~protein targeting	6	3.1746	6.75E-02	FYB, ARL6IP1, AKT1, ATG4C, FXC1, PPP3CB	138	215	13528	2.74	0.99	67.90

Annotation Cluster 2 **Enrichment Score: 1.946654262059368**

Category	Term	Count	%	PValue	Genes	List Total	Pop Hits	Pop Total	Fold Enrichment	Benjamini	FDR
GOTERM_CC_FAT	GO:0015630~microtubule cytoskeleton	16	8.4656	3.54E-04	SDCCAG8, SEPT2, KIF5B, TTLL5, DNHD1, CHEK1, DNAH1, WRN, AKT1, PBXIP1, CEP350, ATG4C, NAV1, LYST, FBXO5, CNTLN	128	549	12782	2.91	0.09	0.46
GOTERM_CC_FAT	GO:0005856~cytoskeleton	23	12.169	1.70E-02	FYB, CTTNBP2NL, SDCCAG8, SEPT2, KIF5B, DIAPH1, UBR4, DNHD1, TTLL5, DNAH1, CHEK1, WRN, ZNF174, AKT1, MAST2, PBXIP1, NAV1, CEP350, ATG4C, LYST, FAAH, FBXO5, CNTLN	128	1381	12782	1.66	0.58	20.05
GOTERM_CC_FAT	GO:0043232~intracellular non-membrane-bounded organelle	37	19.577	1.85E-02	CTTNBP2NL, SDCCAG8, SEPT2, HMGN2, DIAPH1, RPS15A, TTLL5, DNAH1, CHEK1, ZNF174, AKT1, DCAF13, PBXIP1, DGCR8, FBXO5, CNTLN, ZWILCH, FYB, EXOSC9, KIF5B, UBR4, SYCE2, DNHD1, WRN, EXOSC1, ATRX, PAPOLA, MAST2, NAV1, APITD1, CEP350, ATG4C, LYST, FAAH, TEP1, RPS10, CORT, TBX19	128	2596	12782	1.42	0.54	21.53
GOTERM_CC_FAT	GO:0043228~non-membrane-bounded organelle	37	19.577	1.85E-02	CTTNBP2NL, SDCCAG8, SEPT2, HMGN2, DIAPH1, RPS15A, TTLL5, DNAH1, CHEK1, ZNF174, AKT1, DCAF13, PBXIP1, DGCR8, FBXO5, CNTLN, ZWILCH, FYB, EXOSC9, KIF5B, UBR4, SYCE2, DNHD1, WRN, EXOSC1, ATRX, PAPOLA, MAST2, NAV1, APITD1, CEP350, ATG4C, LYST, FAAH, TEP1, RPS10, CORT, TBX19	128	2596	12782	1.42	0.54	21.53
GOTERM_CC_FAT	GO:0044430~cytoskeletal part	15	7.9365	9.00E-02	SDCCAG8, SEPT2, KIF5B, TTLL5, DNHD1, CHEK1, DNAH1, WRN, AKT1, PBXIP1, CEP350, ATG4C, NAV1, FBXO5, CNTLN	128	952	12782	1.57	0.68	70.68

Annotation Cluster 3 **Enrichment Score: 1.4567585294932377**

Category	Term	Count	%	PValue	Genes	List Total	Pop Hits	Pop Total	Fold Enrichment	Benjamini	FDR
GOTERM_CC_FAT	GO:0030136~clathrin-coated vesicle	6	3.1746	0.01	SCAMP1, ATP7A, AP1S2, PI4K2A, RAB5A, CLTC	128	132	12782	4.54	0.58	12.44
GOTERM_CC_FAT	GO:0030665~clathrin coated vesicle membrane	4	2.1164	0.02	SCAMP1, AP1S2, PI4K2A, CLTC	128	53	12782	7.54	0.63	18.55
GOTERM_BP_FAT	GO:0006892~post-Golgi vesicle-mediated transport	4	2.1164	0.02	SCAMP1, AP1S2, SCAMP2, CLTC	138	58	13528	6.76	0.99	29.07
GOTERM_CC_FAT	GO:0030135~coated vesicle	6	3.1746	0.02	SCAMP1, ATP7A, AP1S2, PI4K2A, RAB5A, CLTC	128	159	12782	3.77	0.54	24.29

GOTERM_CC_FAT	GO:0030140~trans-Golgi network transport vesicle	3	1.5873	0.02	ATP7A, AP1S2, CLTC	128	24	12782	12.48	0.49	26.56
GOTERM_CC_FAT	GO:0030133~transport vesicle	4	2.1164	0.03	ATP7A, AP1S2, PI4K2A, CLTC	128	66	12782	6.05	0.51	30.76
GOTERM_CC_FAT	GO:0030662~coated vesicle membrane	4	2.1164	0.04	SCAMP1, AP1S2, PI4K2A, CLTC	128	73	12782	5.47	0.54	37.96
GOTERM_CC_FAT	GO:0005768~endosome	8	4.2328	0.04	SCAMP1, ATP7A, SCAMP2, RAB5C, CHMP5, PI4K2A, RAB5A, CYBASC3	128	315	12782	2.54	0.53	39.32
GOTERM_CC_FAT	GO:0030659~cytoplasmic vesicle membrane	5	2.6455	0.05	SCAMP1, AP1S2, PI4K2A, RAB5A, CLTC	128	139	12782	3.59	0.60	48.53
GOTERM_CC_FAT	GO:0012506~vesicle membrane	5	2.6455	0.06	SCAMP1, AP1S2, PI4K2A, RAB5A, CLTC	128	151	12782	3.31	0.65	57.50
GOTERM_CC_FAT	GO:0044431~Golgi apparatus part	7	3.7037	0.07	SCAMP1, ATP7A, AP1S2, SCAMP2, PDGFC, CLIP3, CLTC	128	294	12782	2.38	0.68	62.65
GOTERM_CC_FAT	GO:0012505~endomembrane system	13	6.8783	0.09	ARL6IP1, SCAMP1, RGPD6, RGPD5, RGPD8, SCAMP2, CLTC, AP1S2, STT3A, INSIG2, SERINC1, PI4K2A, RAB5A, PDGFC, ASPH	128	782	12782	1.66	0.69	69.95
GOTERM_CC_FAT	GO:0005798~Golgi-associated vesicle	3	1.5873	0.09	ATP7A, AP1S2, CLTC	128	51	12782	5.87	0.67	71.19

Annotation Cluster 4 **Enrichment Score: 1.2483614903873126**

Category	Term	Count	%	PValue	Genes	List Total	Pop Hits	Pop Total	Fold Enrichment	Benjamini	FDR
GOTERM_BP_FAT	GO:0006665~sphingolipid metabolic process	4	2.1164	0.04	GBA2, SERINC1, LASS2, GALC	138	75	13528	5.23	1.00	48.90
GOTERM_BP_FAT	GO:0006643~membrane lipid metabolic process	4	2.1164	0.05	GBA2, SERINC1, LASS2, GALC	138	81	13528	4.84	1.00	55.78
GOTERM_BP_FAT	GO:0006672~ceramide metabolic process	3	1.5873	0.07	GBA2, LASS2, GALC	138	42	13528	7.00	1.00	67.83
GOTERM_BP_FAT	GO:0046519~sphingoid metabolic process	3	1.5873	0.08	GBA2, LASS2, GALC	138	45	13528	6.54	1.00	72.33

Annotation Cluster 5 **Enrichment Score: 1.1612816039563372**

Category	Term	Count	%	PValue	Genes	List Total	Pop Hits	Pop Total	Fold Enrichment	Benjamini	FDR
GOTERM_CC_FAT	GO:0042470~melanosome	4	2.1164	0.06	RAB5C, RAB5A, CLTC, PRDX1	128	89	12782	4.49	0.64	54.53
GOTERM_CC_FAT	GO:0048770~pigment granule	4	2.1164	0.06	RAB5C, RAB5A, CLTC, PRDX1	128	89	12782	4.49	0.64	54.53
GOTERM_CC_FAT	GO:0009898~internal side of plasma membrane	7	3.7037	0.10	AP1S2, MTMR1, MAST2, RAB5C, RAB5A, CLTC, GNG5	128	316	12782	2.21	0.67	72.75

Control Subjects

SIGNIFICANTLY ENRICHED CANONICAL PATHWAYS OF CONTROL SUBJECTS

Ingenuity Canonical Pathways	-log(p-value)	-log(B-H p-value)	Ratio	Molecules
Antigen Presentation Pathway	3.35E00	1.71E00	5.41E-02	HLA-DOB,TAP2
Bile Acid Biosynthesis, Neutral Pathway	1.96E00	8.31E-01	7.69E-02	SCP2

Methionine Degradation I (to Homocysteine)	1.87E00	8.31E-01	6.25E-02	AHCY
Cysteine Biosynthesis III (mammalia)	1.82E00	8.31E-01	5.56E-02	AHCY
Superpathway of Methionine Degradation	1.58E00	8.31E-01	3.23E-02	AHCY
B Cell Development	1.54E00	8.31E-01	2.94E-02	HLA-DOB
tRNA Charging	1.48E00	8.31E-01	2.56E-02	IARS
Graft-versus-Host Disease Signaling	1.4E00	8.31E-01	2.08E-02	HLA-DOB
Autoimmune Thyroid Disease Signaling	1.39E00	8.31E-01	2.04E-02	HLA-DOB
Primary Immunodeficiency Signaling	1.36E00	8.31E-01	1.92E-02	TAP2
Nur77 Signaling in T Lymphocytes	1.32E00	8.31E-01	1.75E-02	HLA-DOB
Regulation of Cellular Mechanics by Calpain Protease	1.32E00	8.31E-01	1.75E-02	ACTN4

GENE ONTOLOGY OF CONTROL SUBJECTS

Annotation Cluster 1 **Enrichment Score: 1.556431804209894**

Category	Term	Count	%	PValue	Genes	List Total	Pop Hits	Pop Total	Fold Enrichment	Benjamini	FDR
GOTERM_CC_FAT	GO:0044432~endoplasmic reticulum part	4	21.053	0.00	TAP2, FOXRED2, HLA-DOB, PORCN	14	347	12782	10.52	0.30	4.79
GOTERM_MF_FAT	GO:0001882~nucleoside binding	5	26.316	0.07	IARS, ACTN4, TAP2, FOXRED2, HLA-DOB	14	1612	12983	2.88	1.00	52.67
GOTERM_CC_FAT	GO:0005783~endoplasmic reticulum	4	21.053	0.07	TAP2, FOXRED2, HLA-DOB, PORCN	14	960	12782	3.80	0.94	52.80
